# A recyclable stereoauxiliary aminocatalyzed strategy for one-pot synthesis of indolizine-2-carbaldehydes

**DOI:** 10.1038/s42004-023-00828-2

**Published:** 2023-02-23

**Authors:** Kui Zeng, Ruhuai Mei, Sebastian Dechert, Lutz Ackermann, Kai Zhang

**Affiliations:** 1 Sustainable Materials and Chemistry, Dept. Wood Technology and Wood-based Composites, University of Göttingen, Göttingen, Germany; 2 Institute of Inorganic Chemistry, University of Göttingen, Göttingen, Germany; 3 Institute for Organic and Biomolecular Chemistry, University of Göttingen, Göttingen, Germany

**Keywords:** Catalyst synthesis, Sustainability, Organocatalysis

## Abstract

Indolizine-carbaldehydes with the easily modifiable carbaldehyde group are important synthetic targets as versatile precursors for distinct indolizines. However, the efficient one-pot construction of trisubstituted indolizine-2-carbaldehydes represents a long-standing challenge. Herein, we report an unprecedented recyclable stereoauxiliary aminocatalytic approach *via* aminosugars derived from biomass, which enable the efficient one-pot synthesis of desired trisubstituted indolizine-2-carbaldehydes *via* [3+2] annulations of acyl pyridines and *α*,*β*-unsaturated aldehyde. Compared to the steric shielding effect from *α*-anomer, a stereoauxiliary effect favored by *β*-anomer of D-glucosamine is supported by control experiments. Furthermore, polymeric chitosan containing predominantly *β*-D-anhydroglucosamine units also shows excellent catalytic performance in aqueous solutions for the conversion of various substrates, large-scale synthesis and catalytic cycling experiments. Thus, our approach advances the existing methodologies by providing a rich library of indolizine-2-aldehydes. In addition, it delivers an efficient protocol for a set of late-stage diversification and targeted modifications of bioactive molecules or drugs, as showcased with 1,2,3-trisubstituted indolizine-2-carbaldehydes.

## Abstract (web summary)

Indolizine-2-carbaldehydes are important precursors for indolizine synthesis, however, their efficient construction remains challenging. Here, a recyclable stereoauxiliary aminocatalyzed strategy for one-pot synthesis of 1,2,3-trisubstituted indolizine-2-carbaldehydes is reported, via [3+2] annulations of acyl pyridines and α,β-unsaturated aldehydes.

## Introduction

**Fig. 1:**
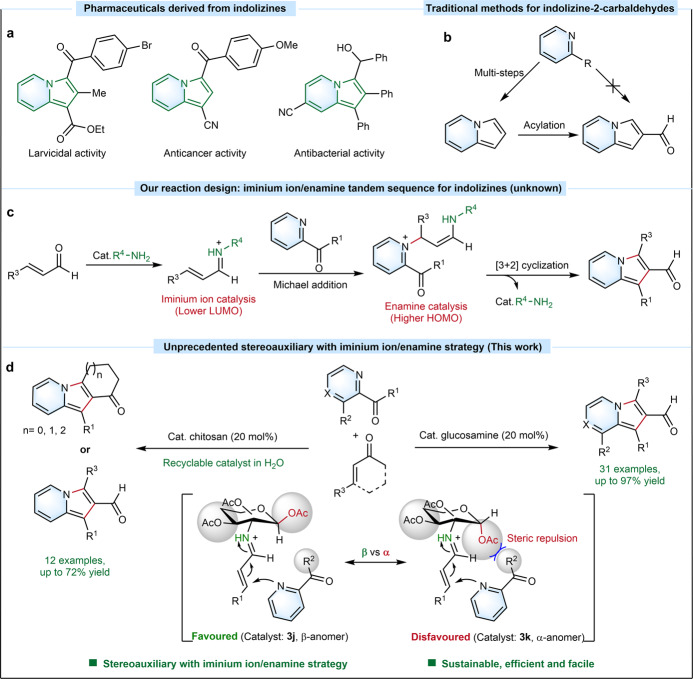
Indolizine. **a** Pharmaceuticals derived from indolizine. **b** Traditional approaches for indolizine-carbaldehydes. **c** Our design: iminium ion/enamine tandem sequence strategy for trisubstituted indolizine-2-carbaldehydes. **d** This work: Unprecedented stereoauxiliary aminocatalysis with iminium ion/enamine strategy for the preparation of 1,2,3-trisubstituted indolizine-2-carbaldehydes *via* one-pot reaction.

Indolizines, an important group of *N*-heterocyclic compounds^1^, play a pivotal role in various fields ranging from pharmaceutics (Fig. 1a)^2–4^ to material science^5^ and chemical synthesis^6–10^. Thus, significant efforts have been made and remarkable progress has been achieved in the synthesis of such type of scaffolds^11^. Four representative strategies are known for the efficient preparation of indolizines, which include Scholtz reaction^12,13^, Tschitschibabin reaction^14,15^, pyridinium *N*-methylides^16,17^, and cyclization of alkynes with heteroaromatic compounds^18–20^. Recently, multi-step synthesis strategies for the preparation of indolizine-carbaldehydes have been reported^21–23^ and the easily modifiable aldehyde group in pyrrole ring makes indolizine-2-carbaldehydes versatile building blocks (Fig. 1b)^22,23^. One-pot synthesis and synthetic modifications of indolizine-2-carbaldehydes, however, were rarely studied, probably due to the lack of efficient synthetic strategies (Fig. 1b). In particular, such an one-pot synthetic strategy would be highly attractive and desired among synthetic and medicinal chemists^22,23^.

The [3+2] annulations of *α*,*β*-unsaturated aldehydes and 2-acetylpyridine is a pivotal step for the one-pot construction of indolizine-2-carbaldehydes. Generally, 2-acetylpyridine easily reacts with the carbonyl group of *α*,*β*-unsaturated aldehydes^24^, and 2-acetylpyridine activated by metal-based Lewis acid could attack the *β*-position of *α*,*β*-unsaturated aldehydes with the presence of a secondary aminocatalyst^25^. These challenges have hampered the development of [3+2] cyclization of 2-acetylpyridine and *α*,*β*-unsaturated aldehydes. Inspired by the two-component Baylis–Hillmann reaction^26,27^, an acetic acid-catalyzed method for the one-pot preparation of desired indolizine-2-carbaldehydes was first time reported in 2021 as a state-of-the-art method^28^. This is the only one-pot synthesis of indolizine-2-carbaldehydes reported to date, and the reaction was carried out in acetic acid as catalyst and solvent to improve the efficiency^29^. A generalized strategy to overcome the harsh reaction conditions for broader scope of indolizine-2-carbaldehydes with even higher efficiency *via* [3+2] cyclization is still highly desired.

During the last decades, aminocatalysis *via* iminium ion or enamine has emerged as an important approach for the construction of various C–C bonds^30–36^. Herein, we propose an aminocatalysis mode *via* iminium ion/enamine tandem sequence that could efficiently overcome the reaction energy barriers for Michael reaction and aldol reaction for the construction of 1,2,3-trisubstituted indolizine-2-carbaldehydes (Fig. 1c). In particular, carbohydrates as the most abundant and renewable biomass with native chiral backbones have been widely utilized as carbohydrate-derived ligands for enantioselective reactions^37–39^, whereas aminocatalyst derived from amino sugars has received less attention so far^40,41^. Inspired by our recently work on anomeric stereoauxiliary cleavage of the C−N bond of glucosamine for the efficient preparation of imidazo[1,5-a]pyridines^42^, we discovered a novel sustainable aminocatalysis strategy *via* recyclable stereoauxiliary combined with iminium ion/enamine tandem sequence as potential synthesis strategy (Fig. 1d). D-glucosamine and even the polymeric chitosan containing mostly *β*-D-anhydroglucosamine units as building blocks representing one of the most abundant and renewable biobased compounds^43^, were first time utilized as attractive stereoauxiliary aminocatalysts for the one-pot efficient synthesis of 1,2,3-trisubstituted indolizine-2-carbaldehydes *via* [3+2] cyclization. This new approach largely expands the scope of readily accessible indolizine-2-carbaldehydes relative to existing state-of-the-art methods.

## Results and discussion

### Reaction development

**Fig. 2:**
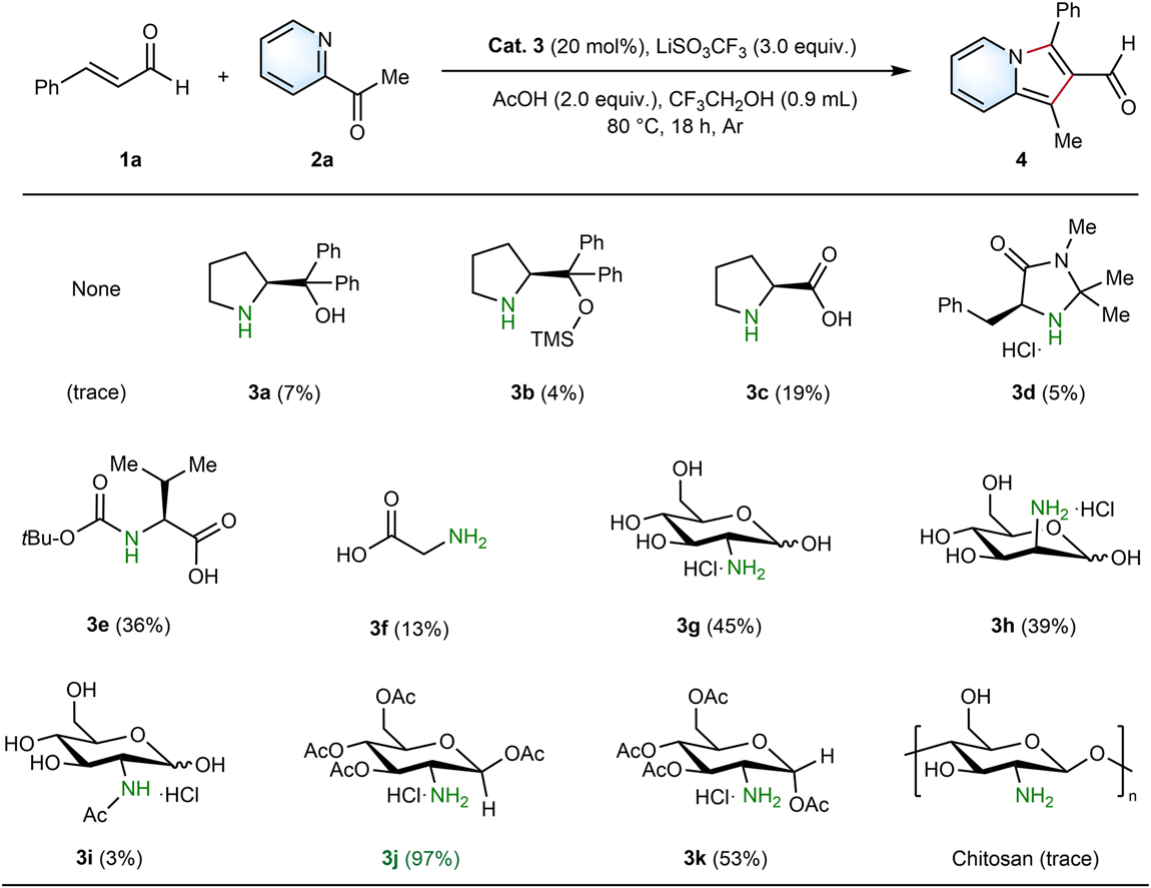
Optimization of the aminocatalyzed [3+2] annulations for indolizine-2-aldehyde. ^a^**1a** (0.2 mmol), **2a** (2.5 equiv.), aminocatalyst (20 mol%), LiSO_3_CF_3_ (3.0 equiv.), AcOH (2.0 equiv.), CF_3_CH_2_OH (0.9 mL), Ar, 18 h, 80 °C. ^b^Yields were determined by ^1^H-NMR analysis with CH_2_Br_2_ as internal standard. Chitosan has a degree of deacetylation of 97.96%.

We initiated our studies using cinnamaldehyde (**1a**) and 2-acetylpyridine (**2a**) as substrates to evaluate the envisioned aminocatalyzed [3+2] cyclization reaction for the synthesis of desired 1-methyl-3-phenylindolizine-2-carbaldehyde (**4**) (see Supplementary Note 1 and Supplementary Method 1, 2). In addition, Brønsted acid (2 equiv.) was used to hinder the deprotonation of the methyl group of **2a** (Supplementary Tables 1, 2)^29^, while lithium cations were used to improve the catalytic performance of the cyclization reaction (Supplementary Table 3)^25,44^. Bronsted acid, e.g., Li^+^, could help to activate the carbonyl group in the iminium formation and/or in the intramolecular cyclization, with the release of water. At the outset without catalyst, the reaction was tested with a trace yield of product **4** with a mixture of **1a** (0.20 mmol), **2a** (2.5 equiv.), LiSO_3_CF_3_ (3.0 equiv.) and acetic acid (2.0 equiv.) in CF_3_CH_2_OH (0.90 mL) for 18 h under Ar atmosphere (Fig. 2) (Supplementary Table 4, 5). We also examined various widely-used representative aminocatalysts and ligands derived from amino acids (Fig. 2). By using (*S*)-(-)-α, α-Diphenyl-2-pyrrolidinemethanol (**3a**)^45^, (*S*)-(-)-α, α-Diphenylprolinoltrimethylsilyl ether (**3b**)^45^, *L*-proline (**3c**)^30^, (5*S*)-(-)-5-Benzyl-2,2,3-trimethylimidazolidin-4-one (**3d**)^31^, *N*-(*tert*-butoxycarbonyl)-*L*-valine (**3e**)^46^ and glycine (**3f**)^47^, as catalysts, only low yields of product **4** were achieved.

Various sustainable amino sugars and their derivatives, including D-glucosamine hydrochloride (**3g**), D-mannosamine hydrochloride (**3h**), *N*-acetyl-D-glucosamine hydrochloride (**3i**), 1,3,4,6-*tetra*-*O*-acetyl-2-amino-2-deoxy-*β*-D-glucopyranose hydrochloride (**3j**), 1,3,4,6-*tetra*-*O*-acetyl-2-amino-2-deoxy-*α*-D-glucopyranose hydrochloride (**3k**) and chitosan were used as aminocatalysts under the same conditions (Fig. 2). Surprisingly, 97% yield of **4** was achieved by using catalyst **3j** (see Supplementary Note 2, Supplementary Fig. 1), while **3k** only achieved 53% yield of **4**. In comparison, lower yields of **4** were obtained with **3g**-**3i**, **3k** and chitosan. Based on all these results, **3j** was taken as the optimal aminocatalyst for further synthesis. In addition to amine-containing catalysts showing the central function for the efficient reaction, acetic acid plays an important role. Without acetic acid (Supplementary Table 2), the yield of **4** decreased obviously from 97 to 44%^48^. As well, the amount of LiSO_3_CF_3_ (2 equiv.) and 2-acetylpyridine (1.5 equiv.), reaction time (12 h) and reaction temperature (25 and 50 °C) also affected the yields (Supplementary Table 1, entries 13-17). Furthermore, in order to exclude the Lewis-acid catalytic pathway through acetic acid^29^, a mixture of **1a** (0.2 mmol), **2a** (2.5 equiv.) and NaOAc (3.0 equiv.) in acetic acid (0.9 mL) was tested (Supplementary Table 4, entries 8). As a result, only 2% of **4** was obtained, which further demonstrates the higher catalytic activity of our aminocatalysis protocol.

### Substrate scope

**Fig. 3:**
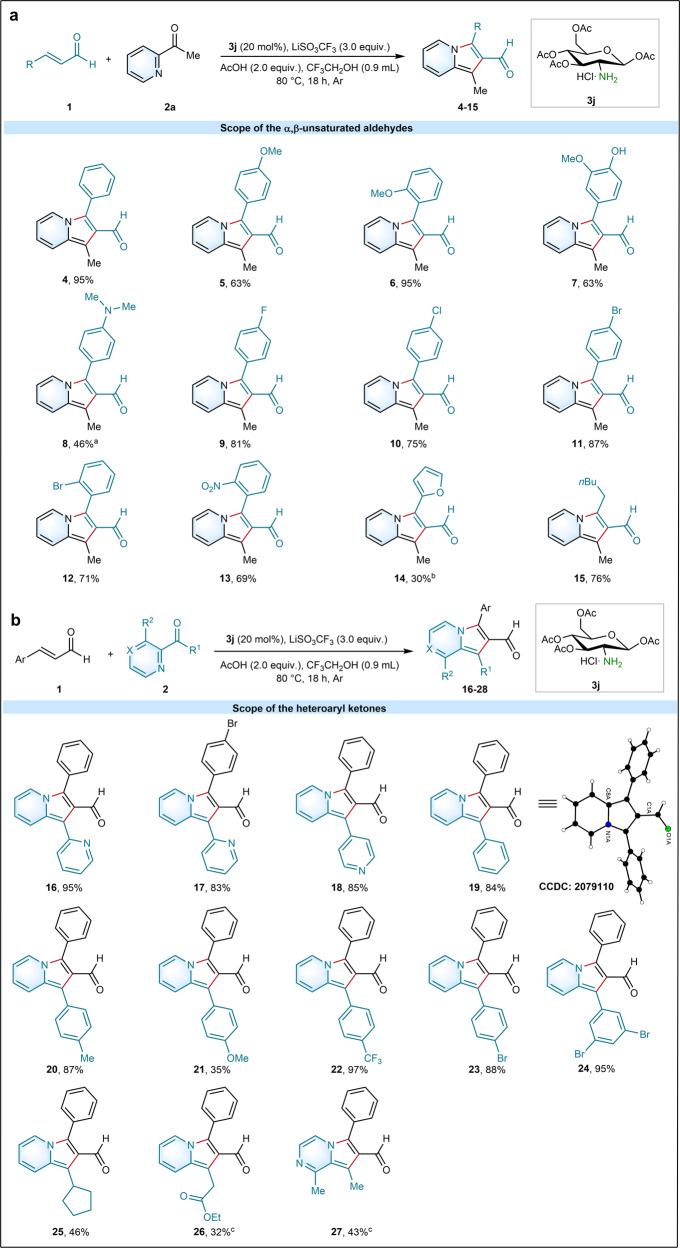
Scope of substrates for the synthesis of indolizine-2-carbaldehydes. **a** Scope of aldehydes. **b** Scope of the heteroaryl ketones. Unless otherwise specified, all products were prepared with **General procedure A**: *α*,*β*-unsaturated aldehyde (0.2 mmol), heteroaryl ketones (2.5 equiv.), catalyst **3j** (20 mol%), AcOH (2.0 equiv.) and LiSO_3_CF_3_ in CF_3_CH_2_OH (0.9 mL) for 18 h at 80 °C under Ar atmosphere. ^**a**^**General procedure B**: *α*,*β*-unsaturated aldehyde (0.2 mmol), heteroaryl ketones (2.5 equiv.), catalyst **3j** (20 mol%), and LiSO_3_CF_3_ in AcOH : CF_3_CH_2_OH (0.4 : 0.5 mL) for 36 h at 80 °C under Ar atmosphere. ^**b**^**General procedure C**: *α*,*β*-unsaturated aldehyde (0.2 mmol), heteroaryl ketones (2.5 equiv.), catalyst **3j** (20 mol%), AcOH (4.0 equiv.) and LiSO_3_CF_3_ in CF_3_CH_2_OH (0.9 mL) for 42 h at r.t.. ^c^**General procedure D**: *α*,*β*-unsaturated aldehyde (0.2 mmol), heteroaryl ketones (2.5 equiv.), catalyst **3j** (20 mol%), AcOH (2.0 equiv.) and LiSO_3_CF_3_ in CF_3_CH_2_OH (0.9 mL) for 36 h at 80 °C under Ar atmosphere. Yields are those of isolated products.

With the optimized reaction conditions in hand, we next probed the scope of various *α*,*β*-unsaturated aldehydes using 2-acetylpyridine as a representative heteroaryl ketone (Fig. 3a) (see Supplementary Method 3 and Supplementary Note 3). A series of *α*,*β*-unsaturated aldehydes, including those with electron-donating or -withdrawing groups at different positions (*ortho*, *meta* or *para*), delivered the corresponding products **4**-**13** under **General procedure A**. An array of valuable products **4**-**8** were efficiently accessed with this stereoauxiliary aminocatalyzed protocol. Notably, in our system, a substrate with an electron-donating methoxy group at *ortho* position (**6**, 95%) could even achieve a higher yield than those at *para* position (**5**, 63%). Surprisingly, a native valuable substrate from *Gliricidia sepium* with a hydroxyl group and a methoxy group was smoothly transformed into a value-added indolizine-2-aldehyde with a moderate yield (**7**, 63%). As well, an important substrate for the detection of catechins was also tolerant under this method with a 46% yield (**8**) under **General procedure B**. In addition, a variety of valuable functional groups at diverse positions, such as fluoro (**9**), chloro (**10**), bromo (**11**, **12**), and nitro moiety (**13**), were well compatible with the standard conditions. Particularly, the sensitive (*E*)-3-(furan-2-yl)acrylaldehyde was also tolerated in our protocol under **General procedure C** and was successfully transformed into the desired product (**14**). Moreover, aliphatic *α*,*β*-unsaturated aldehyde was also well compatible under the optimal conditions (**15**).

We further explored various heteroaryl ketones in combination with cinnamaldehyde under **General procedure A** (Fig. 3b). Di(pyridin-2-yl)methanone and pyridin-2-yl(pyridin-4-yl)methanone were well compatible under the conditions and smoothly achieved yields of 95% (**16**), 83% (**17**) and 85% (**18**), respectively. Diverse aromatic pyridine ketones, including those having electron-donating or -withdrawing groups at distinct positions (*ortho*, *meta*, or *para*) were efficiently transformed into corresponding products (**19**-**24**). Various valuable functional groups at distinct positions (*meta* or *para*), including methoxy (**21**), trifluoromethyl (**22**), bromo (**23**) and dibromo (**24**), were well tolerated under the optimized condition. Cyclopentyl(pyridin-2-yl)methanone efficiently delivered desired product (**25**). The structure of **19** was further confirmed by single-crystal X-ray crystallographic analysis (see Supplementary Fig. 2, Supplementary Table 6), and those of other products in Fig. 3 were assigned by analogy. It is worth noting that ethyl 3-oxo-3-(pyridin-2-yl)propanoate (**26**) and also 1-(3-methylpyrazin-2-yl)ethan-1-one (**27**) were successfully transformed into desired products under **General procedure D**.

### Late-stage synthetic applications

**Fig. 4:**
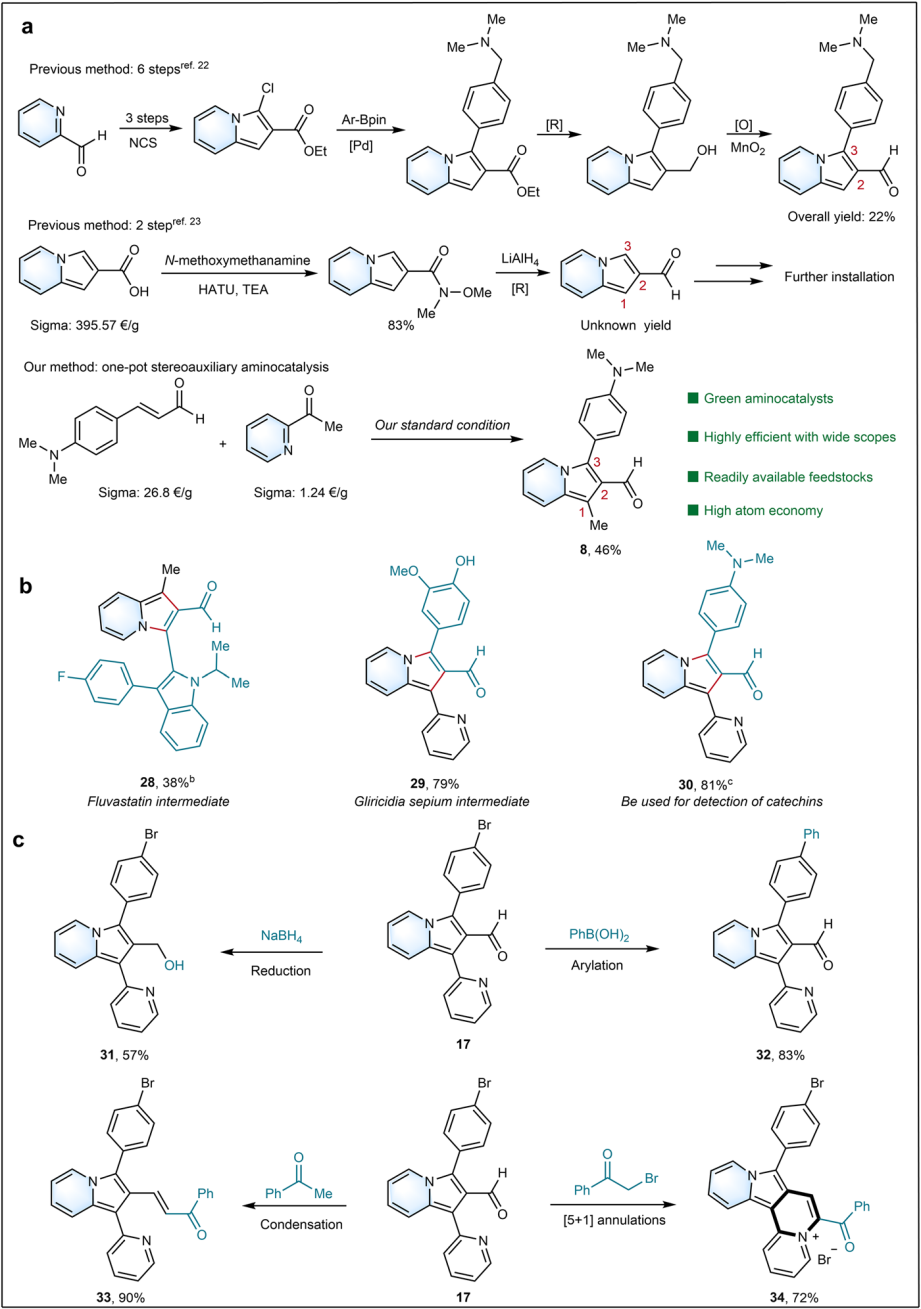
Synthetic applications. **a** Representative previous methods for 3-dimethylaminoindolizine-2-aldehyde. **b** Late-stage selective modifications of bioactive molecules and drugs. **c** Late-stage diversification. ^a^Yields are those of isolated products. ^b^Reaction for 42 h in AcOH : CF_3_CH_2_OH (0.45 : 0.45 mL). ^c^Reaction for 42 h in AcOH : CF_3_CH_2_OH (0.4 : 0.5 mL).

On indolizines with important biological activities, the modifiable aldehyde group on the backbone is attractive for late-stage transformations into versatile value-added products. Until recently, such valuable indolizine-2-carbaldehydes were obtianed in 6-step reaction sequences with complex conditions or 2-step reaction sequences with rare and expensive feedstocks (Fig. 4a)^22,23^. Compared with these previous protocols *via* carboxylation and reduction for the desired products, we efficiently achieved the synthesis of a group of value-added 1,2,3-trisubstituted indolizine-2-carbaldehydes in a one-pot reaction *via* aminocatalyzed [3+2] cyclization reaction. A group of important bioactive molecules or drugs was used for our protocol (Fig. 4b) (see Supplementary Method 4). Surprisingly, an important fluvastatin intermediate was first time accessed by our protocol for the preparation of value-added indolizine-2-carbaldehyde (**28**). As well, (*E*)-3-(4-hydroxy-3-methoxyphenyl)acrylaldehyde from *Gliricidia sepium* was also tolerant under the optimal conditions, which led to 3-(4-hydroxy-3-methoxyphenyl)-1-(pyridin-2-yl)indolizine-2-carbaldehyde (**29**) with 79% yield. Interestingly, (*E*)-3-(4-(dimethylamino)phenyl) acrylaldehyde that is often used to detect catechins^49^ was also smoothly transformed into 3-(4-(dimethylamino)phenyl)-1-(pyridin-2-yl)indolizine-2-carbaldehyde (**30**, 81%). Furthermore, obtained indolizine-2-carbaldehydes could be readily diversified during late-stage modifications, thus providing more complex molecules in an efficient manner (Fig. 4c) (see Supplementary Method 5). For example, 3-(4-bromophenyl)-1-(pyridin-4-yl)indolizine-2-carbaldehyde (**17**) underwent successful reduction (**31**), arylation (**32**), condensation (**33**) or dehydration [5+1] annulations (**34**), to showcase the synthetic diversifications on 1,2,3-trisubstituted indolizine-2-carbaldehydes.

**Fig. 5:**
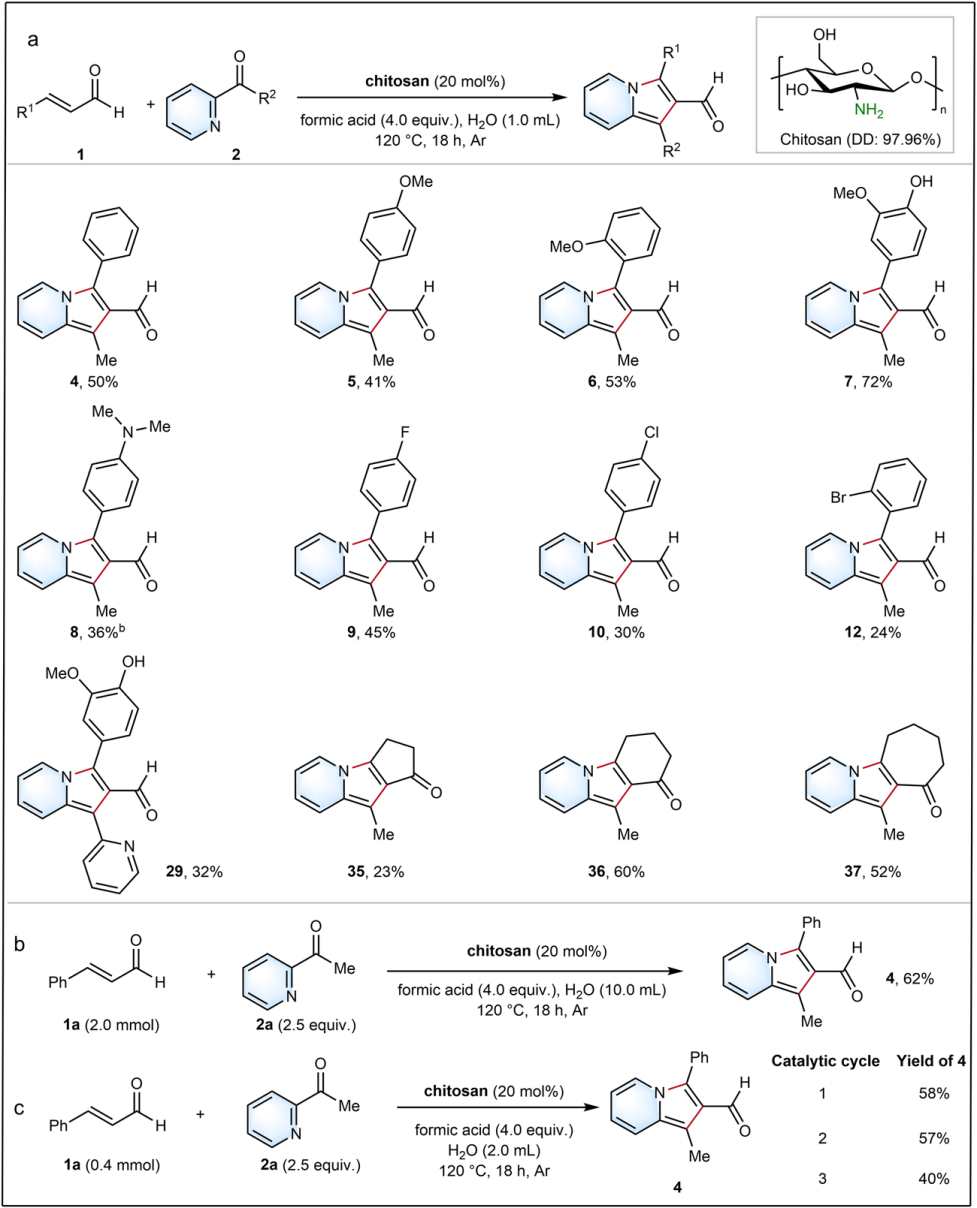
Chitosan as stereoauxiliary aminocatalyst for indolizine-2-carbaldehydes *via* [3+2] annulation. **a** Scope of substrates. ^a^**General procedure E**: *α*,*β*-unsaturated aldehydes/ketones (0.2 mmol), heteroaryl ketones (2.5 equiv.), chitosan (20 mol%) and formic acid (4.0 equiv.) in H_2_O (1.0 mL) for 18 h at 120 °C under Ar atmosphere. ^b^**General procedure F**: *α*,*β*-unsaturated aldehydes/ketones (0.2 mmol), heteroaryl ketones (2.5 equiv.), and chitosan (20 mol%) in Formic acid : H_2_O (0.5 : 0.5 mL) for 36 h at 120 °C under Ar atmosphere. **b** Larger scale synthesis of indolizine-2-carbaldehyde. **c** Cycling catalytic experiments for the synthesis of indolizine-2-carbaldehyde.

Many organocatalyzed reactions still require high catalyst loadings (20-30 mol%), while organocatalysts are difficult to separate, recycle and reuse^32^. Therefore, a recyclable aminocatalyst for desired indolizine-2-aldehydes is in high demand. Notably, our anomeric stereoauxiliary aminocatalyst was efficiently expanded beyond the low molecular weight D-glucosamine to the biopolymer chitosan containing *β*-D-glucosamine as building blocks^50^ (Fig. 5a) (see Supplementary Method 6 and Supplementary Table 7). Interestingly, the use of chitosan demonstrates a recyclable aminocatalysis strategy and the reaction is highly efficient in H_2_O, while lithium salts are not required. As a result, various indolizine-aldehydes were obtained under the use of chitosan as sustainable aminocatalyst, such as products **4** (50%), **5** (41%), **6** (53%), **7** (72%), **8** (36%), **9** (45%), **10** (30%), **12** (24%), and **29** (32%). Even three cyclic *α*,*β*-unsaturated ketones efficiently delivered the corresponding products **35** (23%), **36** (60%), and **37** (52%). Although the use of chitosan for transforming halogenated aromatic *α*,*β*-unsaturated aldehydes led to lower yields compared to glucosamine (Fig. 5a), e.g., for **9** (81%), **10** (75%), and **12** (71%), chitosan as aminocatalyst resulted in higher yields for products **4**-**7** and **35**-**37**.

Our strategy was compared with the state-of-the-art method^28^. For example, products with sensitive groups can be smoothly prepared with our protocol (**7**: 63%, **8**: 46%, **14**: 30%, **26**: 32%, **27**: 43% and **36**: 60%), while only 2% NMR yield or even no products were obtained using the reaction condition as in the ref. ^28^ (**7**: not detected, **8**: 2%, **14**: not detected, **26**: not detected, **27**: 2% and **36**: 8%). These results clearly demonstrated the robustness of our aminocatalysis protocol compared with ref. ^28^. Furthermore, product **4** can be successfully prepared by a one-pot method on a larger scale (2.0 mmol) with up to 62% yield (Fig. 5b) (see Supplementary Method 6). Chitosan can be used for multiple cycles as an aminocatalyst in the aqueous solution, and exhibited excellent catalytic performance even after 3 catalytic cycles under the standard conditions (see Supplementary Fig. 3). During the cycling catalytic reactions, product **4** can be easily isolated by organic solvent extraction, and the remaining aqueous phase can be directly used in the next catalytic cycle after adding **1a** and **2** (Fig. 5c).

### Mechanistic considerations

**Fig. 6:**
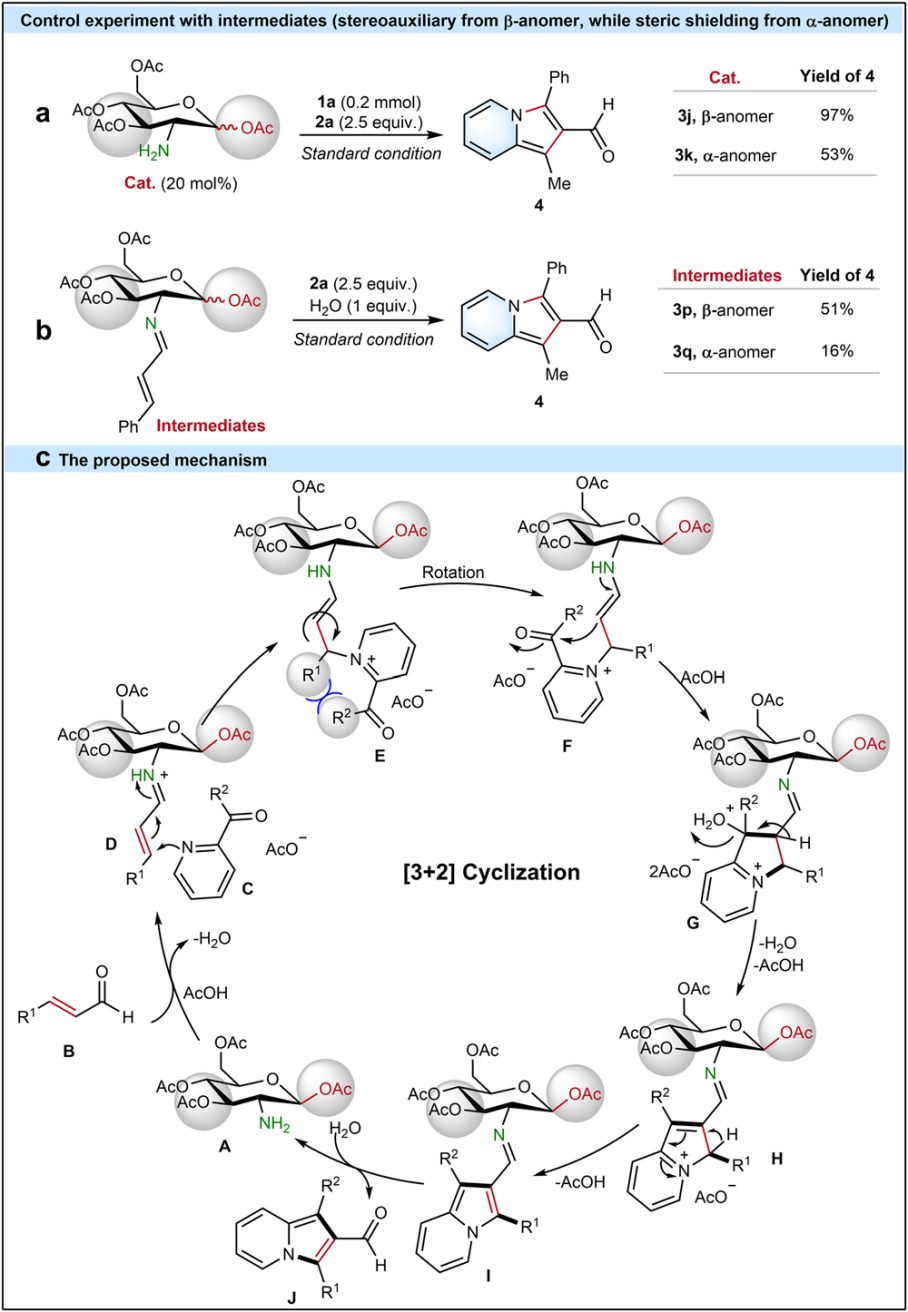
Stereoauxiliary control experiments. **a** Control experiment with **3j** (β-anomer) and **3k** (α-anomer). **b** Control experiment with intermediate **3p** (β-anomer) and intermediate **3q** (α-anomer). **c** Proposed mechanism.

Under the standard condition, catalyst **3j** with *β*-anomer smoothly achieved 97% yield of **4**, while catalyst **3k** with *α*-anomer only yielded 53% of **4** (Fig. 6a). This lower reactivity using **3k** demonstrates the presence of a strong steric shielding from *α*-anomer that affects the efficient conversion to the desired product **4**. To gain more insight into the reaction mechanism, imine intermedates of acetylated D-glucosamine, **3p** as *β*-anomer and **3q** as *α*-anomer, were synthesized, separated and tested under the standard conditions (Fig. 6b) (see Supplementary Method 7). Interestingly, product **4** with 51% yield was obtained using **3p** (*β*-anomer), while **3q** (*α*-anomer) could only deliver 16% yield of **4**. Thus, the imine reaction pathway *via* aminocatalyst preferentially reacting with *α*,*β*-unsaturated aldehydes is verified by these control experiments. Besides, the lower yield of **4** with **3q** (*α*-anomer) further provides a strong support for the existing steric hinderance from acetyl group at C_1_-position in **3q**. In comparison, the stereoauxiliary effect from **3p** (*β*-anomer) promoted the yield of **4**. Therefore, a stereoauxiliary effect favored by *β*-anomer as well as a steric shielding effect from *α*-anomer were clearly verified by control experiments.

Combining all results, a plausible mechanism is proposed (Fig. 6c). First, aminocatalyst **A** reacts with *α*,*β*-unsaturated aldehyde **B** to form iminium ion **D**^32^. Then, 2-acetylpyridine attacks the iminium ion **D** *via* Michael addition reaction to generate an enamine **E**^32,51^. Enamine **F** can be simply converted from **E** *via* the rotation, which will overcome the bulky steric hindrance between R^1^ and R^2^. Thereafter, an intermediate **G** forms *via* the intramolecular cyclization reaction in the enamine **F**. Then, an intermediate **H** generates from the intermediate **G** through a dehydration reaction, which leads to an intermediate **I** after deprotonation. Finally, the desired indolizine-2-aldehyde **J** forms *via* the hydrolysis reaction of intermedidate **I** and the catalyst **A** is regenerated (ESI-HRMS: m/z calcd. for C_14_H_22_NO_9_^+^ [M]: 348.1289, found 348.1297. see Supplementary Fig. 4; the conformation stability of catalyst **3j** was proved with ^1^H NMR in Supplementary Fig. 5) for the next catalysis cycle. Computational investigations of the mechanistic and stereochemical aspects of this study are underway in the Houk lab at UCLA.

## Conclusion

We have developed an unprecedented recyclable anomeric stereoauxiliary aminocatalytic approach using glucosamine/chitosan from biomass for the efficient one-pot preparation of versatilely decorated indolizine-2-carbaldehydes *via* [3+2] annulations of acyl pyridines and *α*,*β*-unsaturated aldehyde. This approach *via* an aminocatalysis pathway under mild conditions efficiently expands the scope of readily accessible trisubstituted indolizine-2-carbaldehydes relative to existing state-of-the-art methods. Mechanistic control studies provided strong support for the anomeric stereoauxiliary catalysis. Furthermore, a plethora of late-stage diversification and targeted modifications of bioactive molecules or drugs showcased the synthetic power of 1,2,3-trisubstituted indolizine-2-carbaldehydes that were assembled *via* this robust stereoaxuliary aminocatalysis approach. Moreover, biopolymer chitosan consisting of *β*-D-anhydroglucosamine units showed excellent catalytic performance in aqueous solution for various substrate diversifications, large-scale synthesis and recycling experiments. Overall, our anomeric stereoauxiliary catalytic approach provides a promising solution and an efficient green synthesis strategy towards addressing the challenges associated with the assembly of indolizine-2-aldehydes with versatile functional moieties, on which ongoing work is targeted to apply this strategy towards developing a wider range of catalytic applications.

## Methods

### Preparation of indolizine-2-carbaldehydes derivatives

#### General procedure A

A mixture of *α*,*β*-unsaturated aldehyde (0.2 mmol), heteroaryl ketone, catalyst **3j** (0.04 mmol), AcOH (2.0 equiv.) and LiSO_3_CF_3_ (3.0 equiv.) in the CF_3_CH_2_OH (0.9 mL) were stirred at 80 °C under Ar atmosphere for 18 h.

#### General procedure B

A mixture of *α*,*β*-unsaturated aldehyde (0.2 mmol), heteroaryl ketone, catalyst **3j** (0.04 mmol) and LiSO_3_CF_3_ (3.0 equiv.) in the CF_3_CH_2_OH : AcOH (0.5 : 0.4 mL) were stirred at 80 °C under Ar atmosphere for 36 h.

#### General procedure C

A mixture of *α*,*β*-unsaturated aldehyde (0.2 mmol), heteroaryl ketone, catalyst **3j** (0.04 mmol), AcOH (4.0 equiv.) and LiSO_3_CF_3_ (3.0 equiv.) in the CF_3_CH_2_OH (0.9 mL) were stirred at room temperature under Ar atmosphere for 42 h.

#### General procedure D

A mixture of *α*,*β*-unsaturated aldehyde (0.2 mmol), heteroaryl ketone, catalyst **3j** (0.04 mmol), AcOH (2.0 equiv.) and LiSO_3_CF_3_ (3.0 equiv.) in the CF_3_CH_2_OH (0.9 mL) were stirred at 80 °C under Ar atmosphere for 36 h.

#### Workup General procedure A–D

The reaction temperature was directly read from temperature detector of IKA apparatus that was calibrated with thermometer. After cooling to room temperature, the reaction mixture was basified up to pH 7 *via* stad. Na_2_CO_3_ aqueous solution, then extracted by diether (3 × 3 mL) and dried over anhydrous Na_2_SO_4_. After filtration and concentration on rotary evaporator, the crude product was purified with flash chromatography on silica gel (ethyl acetate : *n*-hexane) to give products.

#### General procedure E

A mixture of *α*,*β*-unsaturated aldehyde/*α*,*β*-unsaturated ketone (0.2 mmol), heteroaryl ketone (2.5 equiv.), catalyst chitosan (0.04 mmol), formic acid (4.0 equiv.) in H_2_O (1.0 mL) were stirred at 120 °C under Ar atmosphere for 18 h.

#### General procedure F

A mixture of *α*,*β*-unsaturated aldehyde/*α*,*β*-unsaturated ketone (0.2 mmol), heteroaryl ketone (2.5 equiv.), catalyst chitosan (0.04 mmol) in formic acid : H_2_O (0.5 : 0.5 mL) were stirred at 120 °C under Ar atmosphere for 36 h.

#### Workup for General procedure E–F

The reactions were conducted in a sealed Schlenk tube and heated by an IKA magnetic heating agitator with heating block. The reaction temperature was directly read from temperature detector of IKA apparatus that was calibrated with thermometer. After cooling to room temperature, the reaction mixture was extracted by diether (3 × 3 mL) and dried over anhydrous Na_2_SO_4_. After filtration and concentration on rotary evaporator, the crude product was purified with flash chromatography on silica gel (ethyl acetate : *n*-hexane) to give products.

## Supplementary information

Supplementary Information

Description of Additional Supplementary Files

Supplementary Data 1

Supplementary Data 2

## Data Availability

The data that support the findings of this study are available in the Supplementary Information (experimental procedures and characterization data). The NMR spectra of all compounds are available in Supplementary Data 1. The X-ray crystallographic coordinates for structures **19**, reported in this study have been deposited at the Cambridge Crystallographic Data Center (CCDC), under CCDC 2079110 (**19**, Supplementary Data 2). These data can be obtained free of charge from The Cambridge Crystallographic Data Centre via www.ccdc.cam.ac.uk/structures.
