# Supplementary material for: A recyclable stereoauxiliary aminocatalyzed strategy for one-pot synthesis of indolizine-2-carbaldehydes
Source: Commun Chem. 2023 Feb 23;6:40. doi: 10.1038/s42004-023-00828-2 (PMC9950359; doi:10.1038/s42004-023-00828-2)
Supplement: Supplementary file 1 — Supplementary Information [file 42004_2023_828_MOESM1_ESM.pdf]

SUPPLEMENTARY INFORMATION

A recyclable stereoauxiliary aminocatalyzed strategy for one-pot synthesis of indolizine-2-carbaldehydes

Kui Zeng[1], Ruhuai Mei[1], Sebastian Dechert[2], Lutz Ackermann,[3] Kai Zhang[1]✉

Kui Zeng[1], Ruhuai Mei[1], Kai Zhang[1]✉

[1]Sustainable Material and Chemistry, Dept. Wood Technology and Wood-based Composites, University of Göttingen, Göttingen, Germany ✉E-mail: kai.zhang@uni-goettingen.de

Sebastian Dechert[2]

[2]Institut of Inorganic Chemistry, University of Göttingen, Göttingen, Germany

Lutz Ackermann[3]

[3]Institut for Organic Biomolecular Chemistry, University of Göttingen, Göttingen, Germany

Contents

Supplementary Note 1 .... S3

General information S3

Supplementary Method 1 .... S4

Experimental Procedures for Heteroaryl Ketones Derivatives S4

Supplementary Method 2 .... S4

Optimization of Reaction Conditions. S4

Supplementary Note 2 .... S8

^{1}H NMR Analysis of the Crude Solution S8

The X-ray Data and Crystal Structure S9

Supplementary Method 3 .... S11

Experimental Procedures for Indolizine-2-carbaldehydes Derivatives S11

Supplementary Method 4 .... S12

Late-stage Modifications of Bioactive or Drug Molecules. S12

Supplementary Method 5 .... S15

Late-stage Transformation Applications. S15

Supplementary Method 6 .... S19

Optimization of the Chitosan-catalyzed [3+2] Annulations for Indolizine S19

Large-scale Synthesis and Catalytic Cycling Reactions. S19

Supplementary Method 7 .... S22

Preparation of the Intermediates 3p and 3q S22

HR-MS Analysis of Catalyst 3j After Catalytic Reactions S23

^{1}H NMR Analysis of the Conformation Stability of β-anomer S24

Supplementary Note 3 .... S24

Characterization of Products S24

Supplementary References S42

Supplementary Note 1

General information

Unless otherwise specified, the chemicals were obtained commercially and used without further purification. It will be mentioned if there is any further purification for the chemicals. Chitosan was purchased from HEPPE MEDICAL CHITOSAN GmbH. The degree of deacetylation (DD) of chitosan is 97.96% (DD: 97.96%). Practical grade of chitin from shrimp shells and crab shells was purchase from Sigma-Aldrich (Product number C7170). The DD of chitin is 0.025 (DD: 2.5%), which was tested by solid ^{13}C CP-MAS NMR analysis and provided by our previous experiment.[1] All reactions were performed under an atmosphere of Ar unless specified otherwise. NMR spectra were recorded on a 300 MHz or 400 MHz spectrometer with the sample dissolved in the solvent indicated. Chemical shifts (δ) are provided in ppm. Coupling constants are reported in Hertz (Hz) and signal multiplicity is denoted as singlet (s), doublet (d), triplet (t), quartet (q), quintet (quin.), sextet (sex.), septet (sept.), multiplet (m), and broad (br). Yields refer to isolated compounds estimated to be >95% pure as determined by ^{1}H NMR. ESI mass spectra were recorded on Bruker Daltonic micrOTOF. High resolution mass spectra (HR-MS) were recorded on micrOTOF, Bruker Daltonic. FT-IR spectra were recorded on Alpha FT-IR Spectrometer (Bruker, Germany) at room temperature. All samples were measured between 4000 and 500 cm^{-1} with a resolution of 4 cm^{-1} using Platinum ATR and accumulated 24 scans. Melt point were recorded on melting point apparatus, Electrothermal IA 9200.

Supplementary Method 1

Experimental Procedures for Heteroaryl Ketones Derivatives

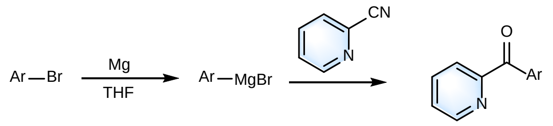

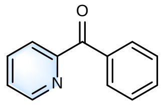

2c

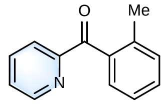

2d

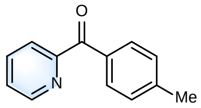

2e

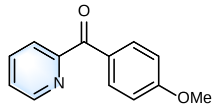

2f

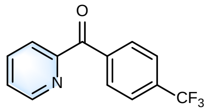

2g

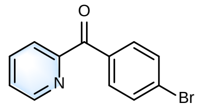

2h

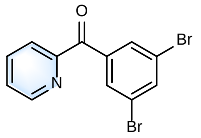

2i

The following starting chemicals (**2c**-**2i**) were synthesized according to previously described methods.[2] A solution of the bromobenzene (10.0 mmol, 1.00 equiv.) in 15 mL of dry THF was dropwised into magnesium (12 mmol, 1.2 equiv.) and stirred in Ar gas in room temperature. After the formation of the Grignard reagent (the color changed to gray), then stopped it. At the same time, carbonitrile (8 mmol, 0.8 equiv.) was dissolved in THF (10 mL), which was dropwised into the mixture solution of Grignard reagent at 0 °C. After that, the reaction was quenched by a solution of saturated NH_4Cl. The organic layer was separated and extracted twice by CH_2Cl_2. After evaporation, the organic layer was redissolved in Et_2O (30 mL) and 6 M HCl (6 mL) was added into the solution. After 30 min, the organic layer was separated, and the aqueous layer was basified by saturated $NaHCO_3$ and then extracted three times by CH_2Cl_2. The combined organic layers were dried over Na_2SO_4 and evaporated in rotary evaporator. The residue was purified by column chromatography with *n*-hexane and ethyl acetate to afford **2c**. Other pyridine ketones **2d**–**2i** were prepared with the similar procedures, and characterized by NMR analysis (More details can be found in our previous research).[3]

Supplementary Method 2

Optimization of Reaction Conditions.

Supplementary Table 1. Optimization of organocatalyzed [3+2]-cyclization.

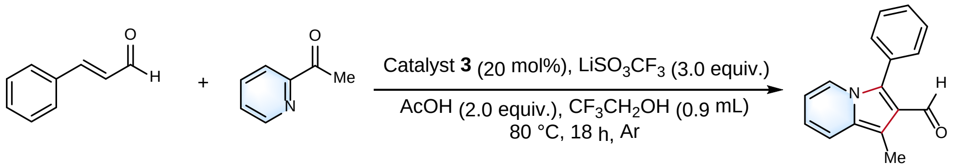

1a, 0.2 mmol **2a**, 2.5 equiv. **4**

Entry	Catalyst (20 mol%)	Acid (equiv.)	Temp./ h	Yield (%)[a]
1	-	AcOH (2.0)	80/ 18	Trace
2	3a	AcOH (2.0)	80/ 18	7
3	3b	AcOH (2.0)	80/ 18	4
4	3c	AcOH (2.0)	80/ 18	19
5	3d	AcOH (2.0)	80/ 18	5
6	3e	AcOH (2.0)	80/ 18	36
7	3f	AcOH (2.0)	80/ 18	13
8	3g	AcOH (2.0)	80/ 18	45
9	3h	AcOH (2.0)	80/ 18	39
10	3i	AcOH (2.0)	80/ 18	3
**11**	**3j**	**AcOH (2.0)**	**80/ 18**	**97**
12	3k	AcOH (2.0)	80/ 18	53
13	chitosan	AcOH (2.0)	80/ 18	trace
14	3j (10 mol%)	AcOH (2.0)	80/ 18	79
15	3j	-	80/ 18	44
16[b]	3j	AcOH (2.0)	80/ 18	82
17[c]	3j	AcOH (2.0)	80/ 18	81
18	3j	AcOH (2.0)	80/ 12	85
19	3j	AcOH (2.0)	25/ 18	32
20	3j	AcOH (2.0)	50/ 18	62

[a]Reactions were carried out at 80 ˚C with **1a** (0.2 mmol), **2a** (0.5 mmol), catalyst (0.02 mmol), $LiSO_3CF_3$ (0.6 mmol) and acetic acid (0.4 mmol) in CF_3CH_2OH (0.9 mL), under Ar gas condition with stirring for 18 h. Yield was determined by ^{1}H NMR analysis with CH_2Br_2 as internal standard. [b]$LiSO_3CF_3$ (0.4 mmol). [c]**2a** (0.3 mmol).

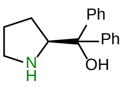

3a

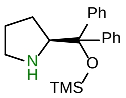

3b

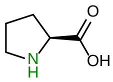

3c

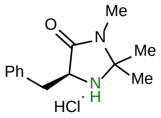

3d

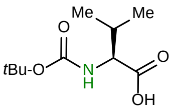

3e

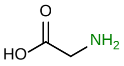

3f

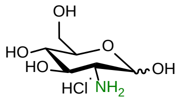

3g

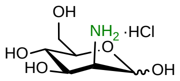

3h

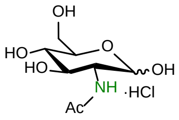

3i

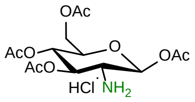

3j

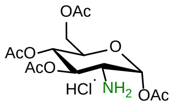

3k

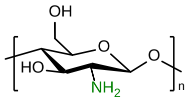

Chitosan

Supplementary Table 2. Optimization of acid.

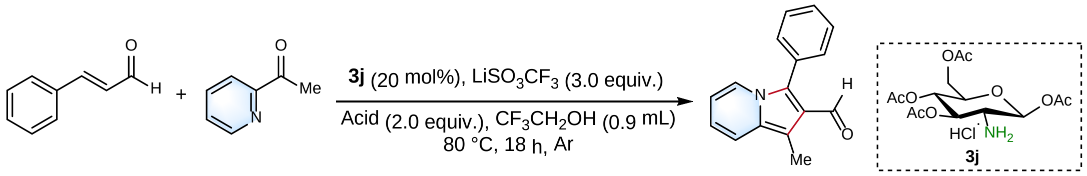

1a, 0.2 mmol **2a**, 2.5 equiv. **4**

Entry	Acid (equiv.)	Yield (%)[a]
1	-	44
2	AcOH (2.0)	97
3	CF_3COOH (2.0)	84
4	HCOOH (2.0)	83
5	CF_3SO_2OH (2.0)	65
6[b]	-	22
7[b]	AcOH (2.0)	53

[a]Reactions were carried out at 80 °C with **1a** (0.2 mmol), **2a** (0.5 mmol), catalyst **3j** (0.02 mmol), acid (2.0 equiv.) and $LiSO_3CF_3$ (0.6 mmol) in CF_3CH_2OH (0.9 mL), under Ar gas condition with stirring for 18 h. Yield was determined by ^{1}H NMR analysis with CH_2Br_2 as internal standard. [b]**3j** (20 mol%) was replaced by **3k** (20 mol%).

Supplementary Table 3. Optimization of metal Lewis acid.

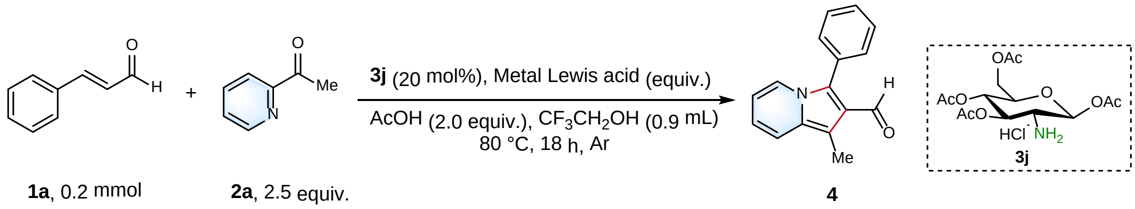

Entry	Metal Lews acid (equiv.)	Yield (%)[a]
1	LiCl (3.0)	86
2	LiBr (3.0)	85
3	LiI (3.0)	72
4	$LiBF_4$ (3.0)	33
5	$LiSO_3CF_3$ (3.0)	97
6	$LiSO_3CF_3$ (2.0)	82

[a]Reactions were carried out at 80 ˚C with **1a** (0.2 mmol), **2a** (0.5 mmol), catalyst **3j** (0.02 mmol), AcOH (2.0 equiv.) and metal Lewis acid (0.6 mmol) in CF_3CH_2OH (0.9 mL), under Ar gas condition with stirring for 18 h. Yield was determined by ^1H NMR analysis with CH_2Br_2 as internal standard.

Supplementary Table 4. Optimization of solvent.

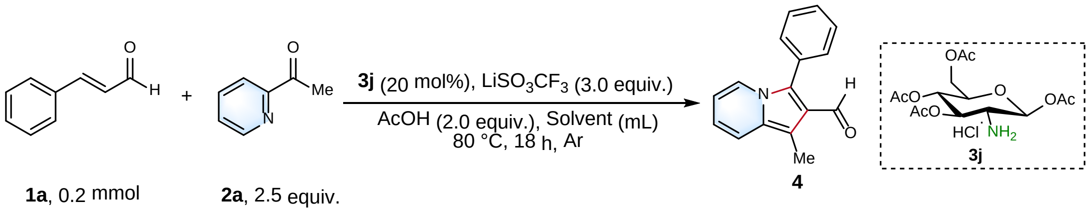

Entry	Solvent (mL)	Yield (%)[a]
1	THF (0.9)	34
2	CF_3CH_2OH (0.9)	97
3	dichloromethane (0.9)	45
4	ethyl acetate (0.9)	41
5	HFIP (0.9)	63
6	CH_3CN (0.9)	62
7	H_2O (0.9)	36
8[b]	AcOH (0.9)	2

[a]Reactions were carried out at 80 ˚C with **1a** (0.2 mmol), **2a** (0.5 mmol), catalyst **3j** (0.02 mmol), AcOH (2.0 equiv.) and $LiSO_3CF_3$ (0.6 mmol) in solvent (0.9 mL), under Ar gas condition with stirring for 18 h. [b]Reactions were carried out at 80 ˚C with **1a** (0.2 mmol), **2a** (0.5 mmol) and NaOAc (0.6 mmol) in AcOH (0.9 mL), under Ar gas condition with stirring for 18 h. Yield was determined by ^1H NMR analysis with CH_2Br_2 as internal standard.

Supplementary Table 5. Optimization of the glucosamine-catalyzed [3+2] annulations for indolizine.[a]

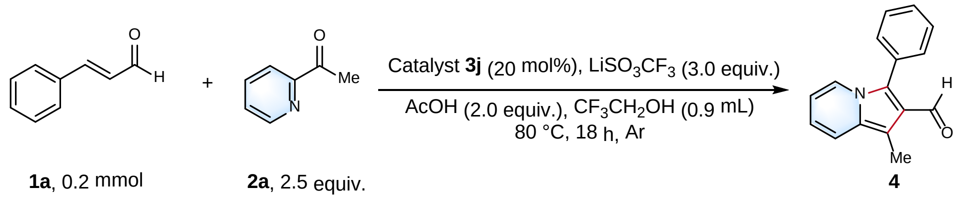

Entry	Variation from standard conditions	**4** (%)[b]
1	no change	97
2	no catalyst **3j**	trace
3	no acetic acid	44
4	no $LiSO_3CF_3$	80
5	no catalyst **3j**, no $LiSO_3CF_3$	n.d.
6	no catalyst **3j**, no acetic acid	n.d.
7	no acetic acid, no $LiSO_3CF_3$	45%
8	no catalyst **3j**, no acetic acid, no $LiSO_3CF_3$	n.d.
9	no catalyst **3j**, no acetic acid, no $LiSO_3CF_3$, 120 °C/48 h	6
10	no catalyst **3j**, no acetic acid, no $LiSO_3CF_3$, no solvent, 120 °C/48 h	n.d.

[a]Reactions were carried out at 80 °C with **1a** (0.2 mmol), **2a** (2.5 equiv.), **3j** (20 mol%), acetic acid (2.0 equiv.), CF_3CH_2OH (0.9 mL), Ar, 18 h, 80 °C. [b]Yield was determined by ^1H NMR analysis with CH_2Br_2 as internal standard.

Supplementary Note 2

^1H NMR Analysis of the Crude Solution

A mixture of **1a** (0.2 mmol), **2a** (2.5 equiv.), catalyst **3j** (0.04 mmol), AcOH (2.0 equiv.) and $LiSO_3CF_3$ (3.0 equiv.) in the CF_3CH_2OH (0.9 mL) were stirred at 80 ºC under an argon atmosphere for 18 h. The reactions were conducted in a sealed Schlenk tube and heated by an IKA magnetic heating agitator with heating block. The reaction temperature was directly read from temperature detector of IKA apparatus and was calibrated by thermometer. After cooling to room temperature, the reaction mixture was basified up to pH 7 *via* stad. Na_2CO_3 aqueous solution, then extracted by diether (3×3 mL) and dried over anhydrous Na_2SO_4. After filtration and concentrated in rotary evaporator, the crude product was dissolved in $CDCl_3$ solution with CH_2Br_2 (10 uL) as the internal standard for ^1H NMR analysis. Based on the NMR analysis, 97% yield of product **4** was obtained. Except for the signal from starting materials and product, no other signal of side-product can be found in the spectrum.

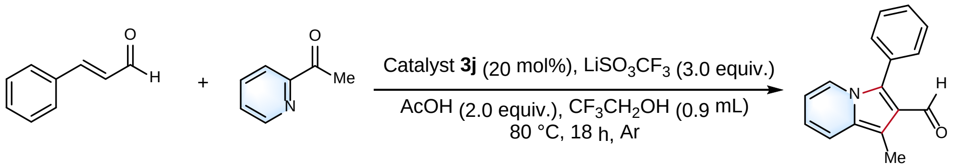

1a, 0.2 mmol **2a**, 2.5 equiv. **4**

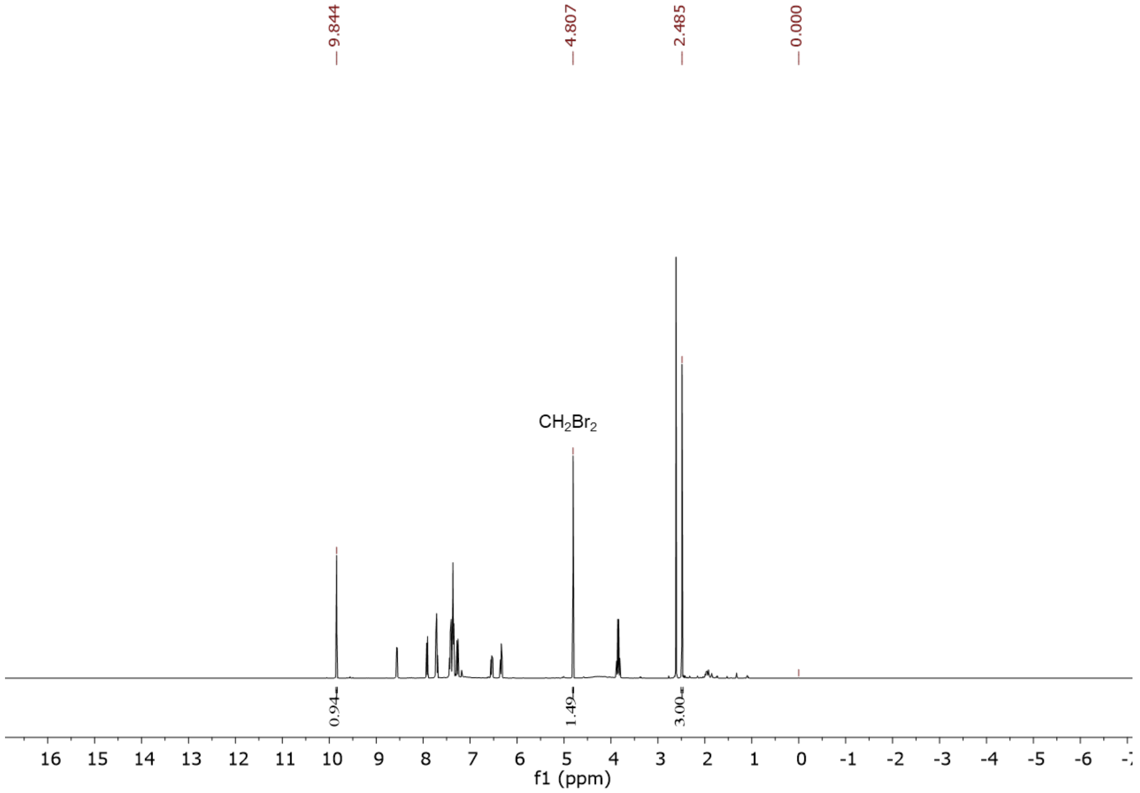

Supplementary Fig. 1 ^{1}H NMR analysis of crude solution. The yield was determined by ^{1}H NMR spectrum analysis and CH_2Br_2 was used as internal standard.

The X-ray Data and Crystal Structure

Crystal data and details of the data collections are given in Supplementary Table 6. X-ray data were collected on a BRUKER D8-QUEST diffractometer (monochromated Mo-Kα radiation, λ = 0.71073 Å) by use of ω and ϕ scans at low temperature. The structures were solved with SHELXT and refined on F^2 using all reflections with SHELXL.[4,5] Non-hydrogen atoms were refined anisotropically. Hydrogen atoms were placed in calculated positions and assigned to an isotropic displacement parameter of 1.2 U_{eq}(C). The aldehyde group (C1 and O1) and the atoms C8 and N1 were found to be disordered (occupancy factors: 0.556(3) / 0.444(3)). EADP (C1A/B, N1A/B, C8A/B) and EXYZ (N1A/B, C8A/B) constraints were applied for most of the atoms. Absorption correction was performed by the multi-scan method with SADABS.[6]

The crystal structure of 1,3-diphenylindolizine-2-carbaldehyde (19).

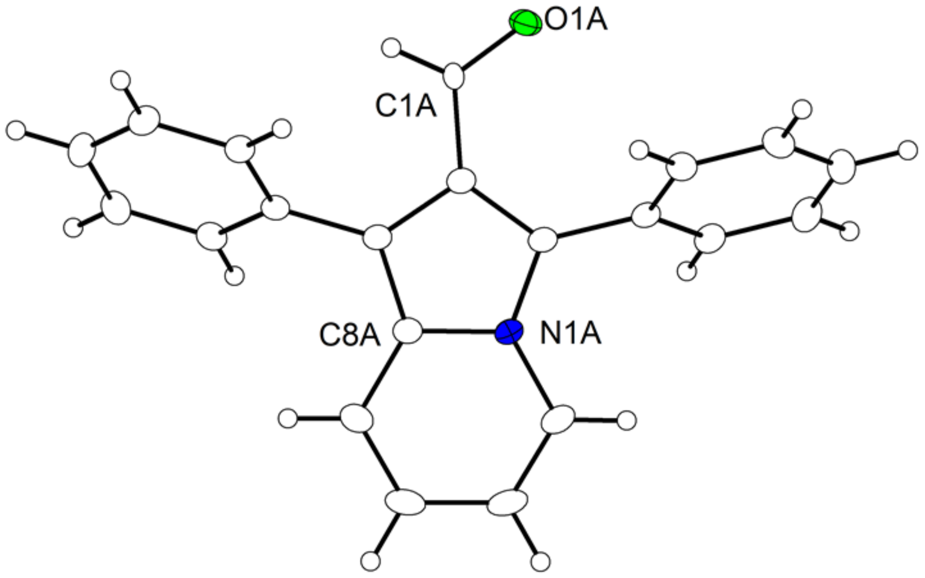

Supplementary Fig. 2 Plot (30% probability thermal ellipsoids) of the molecular structure of the **19** (CCDC: 2079110).

Supplementary Table 6. Crystal data and refinement details for **19**

**compound**	**19** (CCDC: 2079110)
empirical formula	$C_{21}H_{15}NO$
formula weight	297.34
T [K]	133(2)
crystal size [mm³]	0.398 x 0.141 x 0.088
crystal system	monoclinic
space group	$P2_1/c$ (No. 14)
a [Å]	12.1006(5)
b [Å]	16.4078(6)
c [Å]	7.6031(3)
β [°]	96.3910(10)
V [Å³]	1500.17(10)
Z	4
ρ [g·cm⁻³]	1.316
F(000)	624

μ [mm^{-1}]	0.081
T_{min} / T_{max}	0.96 / 0.99
θ–range [°]	2.100 – 27.905
hkl–range	±15, ±21, –9 to 10
measured refl.	35898
unique refl. [R_{int}]	3570 [0.0590]
observed refl. ($I > 2\sigma(I)$)	2773
data / restr. / param.	3570 / 0 / 221
goodness–of–fit (F^2)	1.080
$R1$, $wR2$ ($I > 2\sigma(I)$)	0.0431 / 0.0906
$R1$, $wR2$ (all data)	0.0615 / 0.0988
res. el. dens. [e·Å^{-3}]	–0.244 / 0.146

Supplementary Method 3

Experimental Procedures for Indolizine-2-carbaldehydes Derivatives

General procedure A

A mixture of *α*,*β*-unsaturated aldehyde or ketone (0.2 mmol), heteroaryl ketone (2.5 equiv.), catalyst **3j** (0.04 mmol), AcOH (2.0 equiv.) and $LiSO_3CF_3$ (3.0 equiv.) in the CF_3CH_2OH (0.9 mL) were stirred at 80 °C under Ar atmosphere for 18 h.

General procedure B

A mixture of *α*,*β*-unsaturated aldehyde (0.2 mmol), heteroaryl ketone (2.5 equiv.), catalyst **3j** (0.04 mmol) and $LiSO_3CF_3$ (3.0 equiv.) in the CF_3CH_2OH : AcOH (0.5 : 0.4 mL) were stirred at 80 °C under Ar atmosphere for 36 h.

General procedure C

A mixture of *α*,*β*-unsaturated aldehyde (0.2 mmol), heteroaryl ketone (2.5 equiv.), catalyst **3j** (0.04 mmol), AcOH (4.0 equiv.) and $LiSO_3CF_3$ (3.0 equiv.) in the CF_3CH_2OH (0.9 mL) were stirred at room temperature under Ar atmosphere for 42 h.

General procedure D

A mixture of *α*,*β*-unsaturated aldehyde (0.2 mmol), heteroaryl ketone (2.5 equiv.), catalyst **3j** (0.04 mmol), AcOH (2.0 equiv.) and $LiSO_3CF_3$ (3.0 equiv.) in the CF_3CH_2OH (0.9 mL) were stirred at 80 °C under Ar atmosphere for 36 h.

Workup for General procedure A-D: The reactions were conducted in a sealed Schlenk tube and heated by an IKA magnetic heating agitator with heating block. The reaction temperature was directly read from temperature detector of IKA apparatus and was calibrated by thermometer. After cooling to room temperature, the reaction mixture was basified up to pH 7 *via* stad. Na_2CO_3 aqueous solution, then extracted by diether (3×3 mL) and dried over anhydrous Na_2SO_4. After filtration and concentrated in rotary evaporator, the crude product was purified with flash chromatography on silica gel (ethyl acetate : *n*-hexane) to give products.

General procedure E

A mixture of *α,β*-unsaturated aldehyde or *α,β*-unsaturated ketone (0.2 mmol), heteroaryl ketone (2.5 equiv.), catalyst chitosan (0.04 mmol), formic acid (4.0 equiv.) in H_2O (1.0 mL) were stirred at 120 ºC under Ar atmosphere for 18 h.

General procedure F

A mixture of *α,β*-unsaturated aldehyde or *α,β*-unsaturated ketone (0.2 mmol), heteroaryl ketone (2.5 equiv.), catalyst chitosan (0.04 mmol) in formic acid : H_2O (0.5 : 0.5 mL) were stirred at 120 ºC under Ar atmosphere for 36 h.

Workup for General procedure E-F: The reactions were conducted in a sealed Schlenk tube and heated by an IKA magnetic heating agitator with heating block. The reaction temperature was directly read from temperature detector of IKA apparatus and was calibrated by thermometer. After cooling to room temperature, the reaction mixture was extracted by diether (3×3 mL) and dried over anhydrous Na_2SO_4. After filtration and concentrated in rotary evaporator, the crude product was purified with flash chromatography on silica gel (ethyl acetate : *n*-hexane) to give products.

Supplementary Method 4

Late-stage Modifications of Bioactive or Drug Molecules.
Late-stage preparation of 28

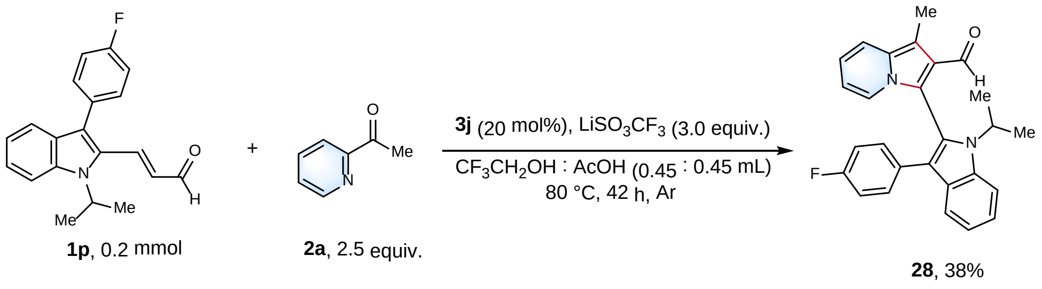

Preparation of **28**: A mixture of **1p** (0.2 mmol), **2a** (2.5 equiv.), catalyst **3j** (0.04 mmol) and $LiSO_3CF_3$ (3.0 equiv.) in CF_3CH_2OH : AcOH (0.45 : 0.45 mL) were stirred at 80 ˚C under Ar atmosphere for 42 h. The reactions were conducted in a sealed Schlenk tube and heated by an IKA magnetic heating agitator with heating block. The reaction temperature was directly read from temperature detector of IKA apparatus and was calibrated by thermometer. After cooling to room temperature, the reaction mixture was basified up to pH 7 *via* stad. Na_2CO_3 aqueous solution, then extracted by diether (3×3 mL) and dried over anhydrous Na_2SO_4. After filtration and concentrated in rotary evaporator, the crude product was purified with flash chromatography on silica gel (ethyl acetate : *n*-hexane) to give products. Work-up gave product **28** (31.2 mg, 0.08 mmol, isolated yield 38%) as a yellow solid. **mp**: 199−200 ˚C. **FT-IR**: *ν* (cm^{-1}): 3039, 2926, 2854, 2743, 2108, 1673, 1599, 1519, 1484, 1475, 1445, 1426, 1395, 1356, 1317, 1259, 1230, 1179, 1123, 1105, 1069, 1039, 1020, 1006, 921, 901, 816, 797, 748, 732, 701, 688, 645, 560, 501, 488, 435, 408. **^{1}H NMR** (400 MHz, $CDCl_3$) δ 9.85 (s, 1H), 7.74 (d, *J* = 8.0 Hz, 1H), 7.61 (d, *J* = 8.4 Hz, 1H), 7.40 – 7.35 (m, 2H), 7.27 (t, *J* = 7.7 Hz, 1H), 7.16 (t, *J* = 7.6 Hz, 1H), 7.05 – 7.01 (m, 2H), 6.82 (t, *J* = 8.5 Hz, 2H), 6.65 – 6.62 (m, 1H), 6.39 (t, *J* = 6.7 Hz, 1H), 4.08 (hept, *J* = 6.9 Hz, 1H), 2.53 (s, 3H), 1.48 (d, *J* = 7.2 Hz, 3H), 1.44 (d, *J* = 6.8 Hz, 3H). **^{13}C NMR** (100 MHz, $CDCl_3$) δ 188.6, 161.5 (d, $^1J_{C-F}$ = 243.9 Hz), 135.8, 131.7, 130.1 (d, $^4J_{C-F}$ = 3.2 Hz), 129.9 (d, $^3J_{C-F}$ = 7.8 Hz), 127.7, 126.2, 123.6, 122.9, 122.7, 120.3, 120.2, 119.7, 119.2, 118.1, 115.5 (d, $^2J_{C-F}$ = 21.2 Hz), 113.4, 112.4, 110.5, 48.8, 22.0, 21.5, 9.6. **^{19}F NMR** (377 MHz, $CDCl_3$) δ -116.1. **ESI-HRMS**: *m/z* calcd. for $C_{27}H_{23}FN_2O$ $[M+H]^+$: 411.1873, found 411.1854.

Late-stage preparation of 29

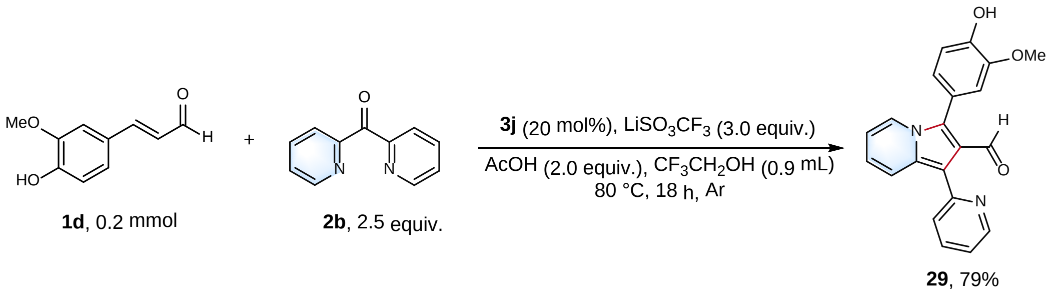

Preparation of **29**: A mixture of **1d** (0.2 mmol), **2b** (2.5 equiv.), catalyst **3j** (0.04 mmol), AcOH (2.0 equiv.) and $LiSO_3CF_3$ (3.0 equiv.) in the CF_3CH_2OH (0.9 mL) were stirred at 80 ˚C under Ar atmosphere for 18 h. The reactions were conducted in a sealed Schlenk tube and heated by an IKA magnetic heating agitator with heating block. The reaction temperature was directly read from temperature detector of IKA apparatus and was calibrated by thermometer. After cooling to room temperature, the reaction mixture was basified up to pH 7 *via* stad. Na_2CO_3 aqueous solution, then extracted by diether (3×3 mL) and dried over anhydrous Na_2SO_4. After filtration and concentrated in rotary evaporator, the crude product was purified with flash chromatography on silica gel (ethyl acetate : *n*-hexane) to give products. Work-up gave product **29** (55.0 mg, 0.16 mmol, isolated yield 79 %) as a yellow liquid gel.

According to the general procedure E, a mixture of **1d** (0.2 mmol), **2b** (2.5 equiv.), catalyst chitosan (0.04 mmol), formic acid (4.0 equiv.) in H_2O (1.0 mL) were stirred at 120 ºC under Ar atmosphere for 18 h. Work-up gave product **29** (22.0 mg, 0.06 mmol, isolated yield 32%) as a yellow liquid gel.

FT-IR: *v* (cm^{-1}): 3017, 2955, 2930, 2848, 2755, 1671, 1587, 1533, 1517, 1475, 1422, 1377, 1346, 1265, 1216, 1168, 1121, 1098, 1057, 1026, 960, 925, 876, 810, 785, 742, 696, 664, 626, 598, 556, 437, 408. **^{1}H NMR** (400 MHz, $CDCl_3$) δ 10.02 (s, 1H), 8.63 (d, *J* = 4.8 Hz, 1H), 7.85 (d, *J* = 9.2 Hz, 1H), 7.80 (d, *J* = 7.2 Hz, 1H), 7.69 – 7.66 (m, 2H), 7.15 – 7.11 (m, 1H), 7.00 – 6.93 (m, 3H), 6.76 (dd, *J* = 9.0, 6.7 Hz, 1H), 6.50 (t, *J* = 6.8 Hz, 1H), 3.83 (s, 3H). **^{13}C NMR** (100 MHz, $CDCl_3$) δ 188.8, 153.2, 149.1, 147.0, 146.8, 135.9, 132.1, 132.0, 125.6, 124.4, 122.9, 122.2, 121.0, 120.8, 120.4, 120.2, 115.0, 113.7, 113.7, 113.6, 56.1. **ESI-HRMS**: *m/z* calcd. for $C_{21}H_{16}N_2O_3$ $[M+H]^+$: 345.1239, found 345.1234.

Late-stage preparation of 30

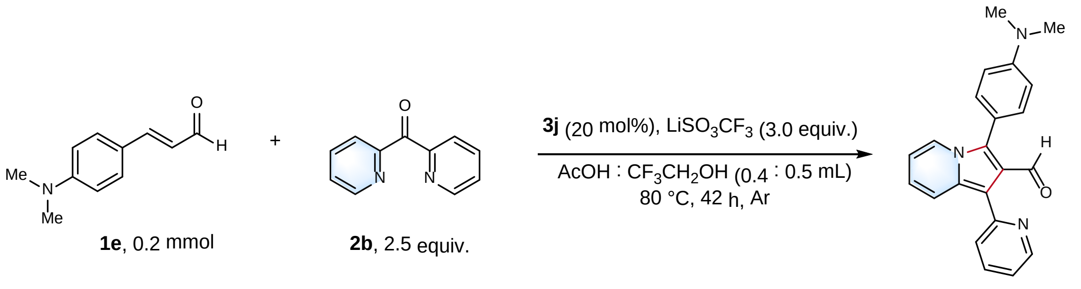

Preparation of **30**: A mixture of **1e** (0.2 mmol), **2b** (2.5 equiv.), catalyst **3j** (0.04 mmol), and $LiSO_3CF_3$ (3.0 equiv.) in CF_3CH_2OH : AcOH (0.5 : 0.4 mL) were stirred at 80 ˚C under Ar atmosphere for 42 h. The reactions were conducted in a sealed Schlenk tube and heated by an IKA magnetic heating agitator with heating block. The reaction temperature was directly read from temperature detector of IKA apparatus and was calibrated by thermometer. After cooling to room temperature, the reaction mixture was basified up to pH 7 *via* stad. Na_2CO_3 aqueous solution, then extracted by diether (3×3 mL) and dried over anhydrous Na_2SO_4. After filtration and concentrated in rotary evaporator, the crude product was purified with flash chromatography on silica gel (ethyl acetate : *n*-hexane) to give products. Work-up gave product **30** (54.6 mg, 0.16 mmol, isolated yield 81%) as a yellow solid. **mp**: 166−167 ˚C. **FT-IR**: ν (cm^{-1}): 3315, 2899, 2848, 2800, 2774, 2188, 1918, 1665, 1605, 1587, 1535, 1521, 1473, 1440, 1418, 1381, 1354, 1327, 1263, 1228, 1203, 1166, 1117, 1096, 1063, 1053, 1034, 1006, 985, 942, 905, 874, 833, 814, 806, 777, 736, 721, 692, 645, 618, 560, 530, 478, 443, 404. **^{1}H NMR** (300 MHz, $CDCl_3$) δ 9.98 (s, 1H), 8.59 (d, *J* = 4.8 Hz, 1H), 7.87 – 7.81 (m, 2H), 7.69 – 7.60 (m, 2H), 7.28 (d, *J* = 8.4 Hz, 2H), 7.09 – 7.04 (m, 1H), 6.76 – 6.67 (m, 3H), 6.43 (t, *J* = 6.8 Hz, 1H). **^{13}C NMR** (75 MHz, $CDCl_3$) δ 188.9, 153.5, 150.7, 148.9, 135.6, 133.7, 131.9, 125.5, 122.9, 121.8, 120.7, 120.6, 120.5, 115.1, 113.2, 113.1, 112.0, 40.1. **ESI-HRMS**: *m/z* calcd. for $C_{22}H_{19}N_3O$ $[M+H]^+$: 342.1606, found 342.1603.

Supplementary Method 5

Late-stage Transformation Applications.
Late-stage transformation of 17 for product 31

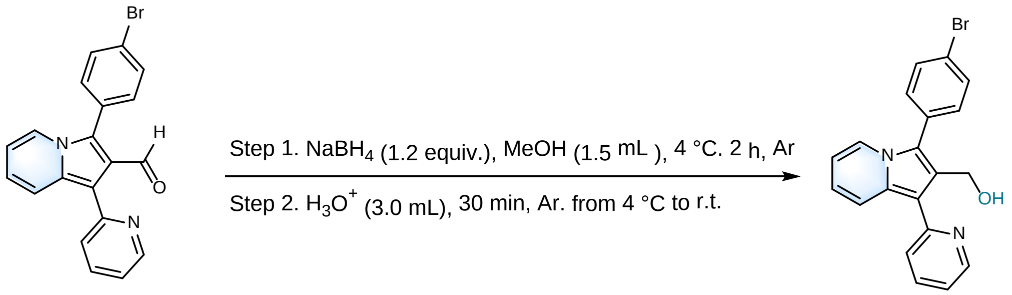

17, 0.1 mmol

31, 57%

Preparation of **31**: A mixture of **17** (0.1 mmol) and $NaBH_4$ (1.2 equiv.) in anhydrous MeOH solution (1.5 mL) were stirred at 4 ºC under Ar atmosphere for 2 h. The reactions were conducted in a sealed Schlenk tube and stirred by an IKA magnetic heating agitator with heating block. The reaction temperature was calibrated by thermometer. After the reaction, the solution was acidified *via* 0.5 N HCl aqueous solution, then stirred for 30 min from 4 ºC to room temperature. After completion, the solution was extracted by diether (3×3 mL) and dried over anhydrous Na_2SO_4. After filtration and concentrated in rotary evaporator, the crude product was purified with flash chromatography on silica gel (ethyl acetate : *n*-hexane) to give products. Work-up gave product **31** (22.7 mg, 0.06 mmol, isolated yield 57%) as a yellow solid. **mp**: 99−100 ºC. **FT-IR**: ν (cm^{-1}): 2922, 2852, 1587, 1537, 1513, 1473, 1389, 1323, 1273, 1228, 1148, 1123, 1100, 1069, 1055, 1037, 1008, 946, 907, 828, 787, 727, 703, 664, 616, 499, 439, 404. **^{1}H NMR** (400 MHz, $CDCl_3$) δ 8.59 (d, *J* = 4.4 Hz, 1H), 7.95 (d, *J* = 6.8 Hz, 1H), 7.78 – 7.67 (m, 3H), 7.61 (d, *J* = 8.0 Hz, 2H), 7.38 (d, *J* = 8.4 Hz, 2H), 7.12 – 7.09 (m, 1H), 6.86 – 6.82 (m, 1H), 6.50 (t, *J* = 6.8 Hz, 1H), 4.52 (s, 1H). **^{13}C NMR** (100 MHz, $CDCl_3$) δ 154.3, 148.6, 137.4, 132.4, 132.2, 131.5, 129.0, 127.7, 123.5, 123.3, 122.7, 122.5, 120.8, 120.0, 117.6, 111.8, 55.9. **ESI-HRMS**: m/z calcd. for $C_{20}H_{15}N_2OBr$ [M+H]+: 379.0446, found 379.0430.

Late-stage transformation of 17 for product 32

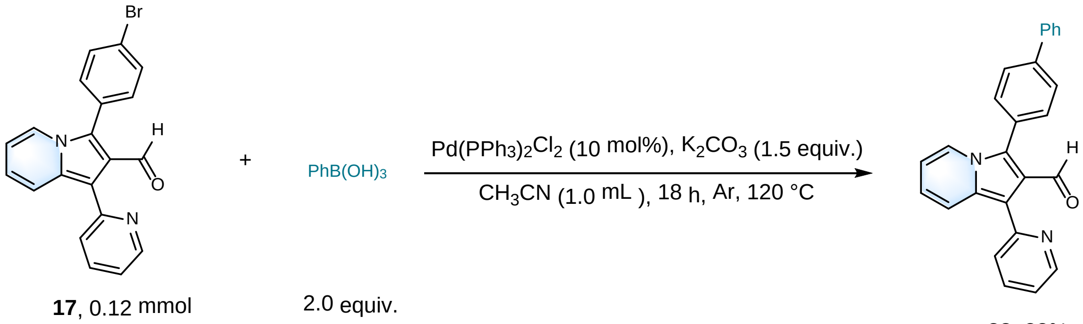

17, 0.12 mmol

2.0 equiv.

32, 83%

Preparation of **32**: A mixture of **17** (0.12 mmol), phenylboronic acid (2.0 equiv.), catalyst $Pd(PPh_3)_2Cl_2$ (10% mmol), and K_2CO_3 (1.5 equiv.) in CH_3CN (1.0 mL) were stirred at 120 ˚C under Ar atmosphere for 18 h. The reactions were conducted in a sealed Schlenk tube and heated by an IKA magnetic heating agitator with heating block. The reaction temperature was directly read from temperature detector of IKA apparatus and was calibrated by thermometer. After cooling to room temperature, the reaction mixture was basified up to pH 7 *via* stad. Na_2CO_3 aqueous solution, then extracted by diether (3×3 mL) and dried over anhydrous Na_2SO_4. After filtration and concentrated in rotary evaporator, the crude product was purified with flash chromatography on silica gel (ethyl acetate : *n*-hexane) to give products. Work-up gave product **32** (37.4 mg, 0.10 mmol, isolated yield 83%) as a yellow solid. **mp**: 146−147 ˚C. **FT-IR**: *ν* (cm^{-1}):3058, 3031, 2920, 2854, 2774, 1673, 1630, 1583, 1519, 1475, 1426, 1416, 1356, 1323, 1280, 1269, 1230, 1197, 1181, 1156, 1142, 1100, 1086, 1055, 1034, 1018, 1008, 946, 907, 882, 853, 820, 785, 767, 740, 725, 694, 647, 622, 587, 571, 550, 505, 495, 439, 420, 404. **^{1}H NMR** (400 MHz, $CDCl_3$) δ 10.09 (s, 1H), 8.64 (d, J = 4.8 Hz, 1H), 7.89 (d, J = 8.4 Hz, 2H), 7.71 – 7.67 (m, 4H), 7.59 (d, J = 7.6 Hz, 2H), 7.55 – 7.46 (m, 3H), 7.41 (t, J = 7.6 Hz, 2H), 7.33 (d, J = 7.2 Hz, 1H), 7.15 – 7.11 (m, 1H), 6.78 (dd, J = 9.6, 6.4 Hz, 1H), 6.53 (t, J = 6.0 Hz, 1H). **^{13}C NMR** (100 MHz, $CDCl_3$) δ 188.7, 153.1, 149.2, 142.0, 140.2, 135.9, 132.3, 131.5, 131.1, 130.3, 128.9, 127.8, 127.7, 127.6, 127.2, 127.1, 125.6, 122.9, 122.4, 121.0, 120.5. **ESI-HRMS**: *m/z* calcd. for $C_{26}H_{18}N_2O$ $[M+H]^+$: 375.1497, found 375.1495.

Late-stage transformation of 17 for product 33

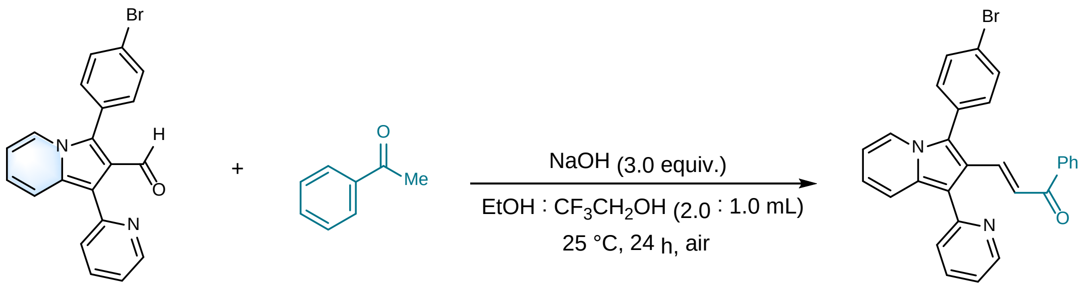

17, 0.05 mmol 3.0 equiv. **33**, 90%

Preparation of **33**: A mixture of **17** (0.05 mmol), acetophenone (3.0 equiv.) and NaOH (3.0 equiv.) in EtOH : CF_3CH_2OH (2.0 : 1.0 mL) were stirred at 25 ºC under air atmosphere for 24 h. The reactions were conducted in a glass tube and stirred by an IKA magnetic heating agitator. The reaction was monitored by TLC. After filtration and concentrated in rotary evaporator, the crude product was purified with flash chromatography on silica gel (ethyl

acetate : *n*-hexane) to give products. Workup gave product **33** (21.5 mg, 0.05 mmol, isolated yield 90%) as a yellow solid. **mp**: 173−174 ºC. **FT-IR**: *v* (cm^{-1}): 3054, 2920, 1655, 1583, 1504, 1471, 1447, 1387, 1341, 1296, 1212, 1177, 1148, 1102, 1072, 1034, 1008, 991, 853, 830, 789, 742, 690, 647, 583, 548, 509, 441, 408. **^{1}H NMR** (400 MHz, $CDCl_3$) δ 8.73 (d, *J* = 4.4 Hz, 1H), 7.91 (d, *J* = 16.0 Hz, 1H), 7.72 – 7.66 (m, 5H), 7.57 (d, *J* = 7.6 Hz, 2H), 7.47 (d, *J* = 7.6 Hz, 1H), 7.42 (t, *J* = 7.1 Hz, 1H), 7.34 – 7.32 (m, 4H), 7.17 (d, *J* = 10.0 Hz, 1H), 6.93 (d, *J* = 15.6 Hz, 1H), 6.77 – 6.73 (m, 1H), 6.48 (t, *J* = 6.8 Hz, 1H). **^{13}C NMR** (100 MHz, $CDCl_3$) δ 189.9, 154.2, 149.9, 138.2, 137.4, 136.4, 132.9, 132.8, 132.5, 132.3, 129.6, 128.4, 128.2, 125.1, 124.7, 124.1, 123.3, 122.3, 121.0, 120.9, 120.1, 119.0, 114.0, 112.8. **ESI-HRMS**: *m/z* calcd. for $C_{28}H_{19}N_2OBr$ $[M+H]^+$: 479.0759, found 479.07740.

Late-stage transformation of 17 for product 34

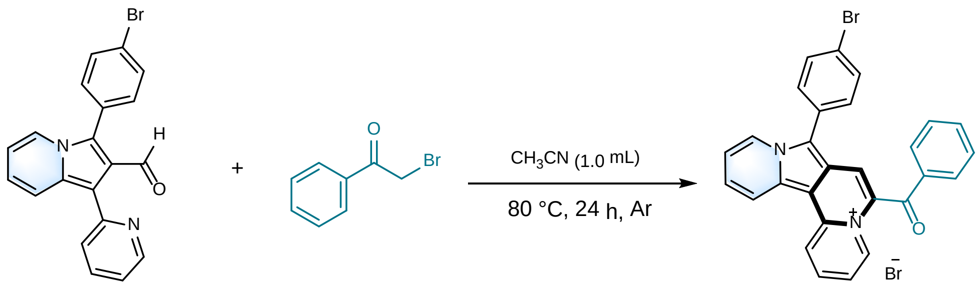

Preparation of **34**: A mixture of **17** (0.05 mmol) and 2-bromo-1-phenylethan-1-one (1.0 equiv.) in CH_3CN (1.0 mL) were stirred at 80 ºC under Ar atmosphere for 24 h. The reactions were conducted in a sealed Schlenk tube and heated by an IKA magnetic heating agitator with heating block. The reaction temperature was directly read from temperature detector of IKA apparatus and was calibrated by thermometer. After cooling to room temperature, resulting precipitate was filtered off, washed with *n*-hexane and washed with ethyl acetate, then gave product **34** (20.0 mg, 0.04 mmol, isolated yield 72%) as a red solid. **mp**: 293−295 ºC. **FT-IR**: *v* (cm^{-1}): 3392, 3060, 1655, 1630, 1597, 1552, 1533, 1492, 1473, 1436, 1354, 1333, 1302, 1245, 1197, 1164, 1142, 1119, 1076, 1057, 1008, 950, 863, 824, 771, 756, 723, 711, 699, 686, 668, 635, 622, 614, 546, 499, 443, 416. **^{1}H NMR** (400 MHz, DMSO-d_6) δ 9.26 (d, *J* = 8.8 Hz, 1H), 9.19 (d, *J* = 6.8 Hz, 1H), 9.11 (d, *J* = 9.2 Hz, 1H), 8.98 (d, *J* = 6.8 Hz, 1H), 8.49 (t, *J* = 7.8 Hz, 1H), 8.15 (s, 1H), 8.09 (d, *J* = 7.6 Hz, 2H), 7.89 – 7.80 (m, 4H), 7.75 – 7.73 (m, 3H), 7.64 (t, *J* = 7.5 Hz, 2H), 7.58 (t, *J* = 6.7 Hz, 1H). **^{13}C NMR** (100 MHz, DMSO-d_6) δ 188.9, 142.1, 140.3, 137.7, 135.6, 135.2, 135.2, 132.8, 132.1, 130.8, 130.4,

129.1, 126.3, 126.1, 125.6, 122.9, 122.8, 120.5, 120.1, 119.8, 119.5, 118.7, 118.3, 104.2. **ESI-HRMS**: *m/z* calcd. for $C_{28}H_{18}N_2OBr^+$ [M]: 477.0603, found 477.0596.

Supplementary Method 6

Optimization of the Chitosan-catalyzed [3+2] Annulations for Indolizine

Supplementary Table 7. Optimization of the chitosan-catalyzed [3+2] annulations for indolizine.[a]

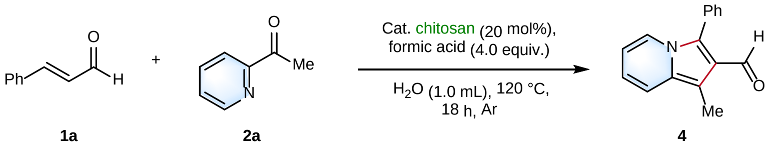

Entry	Variation from standard conditions	**4** (%)[b]
1	no change	51
2	no chitosan as catalyst	trace, 0
3	no formic acid	trace, 0
4	chitin in place of chitosan	3
5	acetic acid in place of formic acid	50
6	CF_3CH_2OH in place of H_2O	15
7	80 °C in place of 120 °C	26
8	140 °C in place of 120 °C	49
9	36 h in place of 18 h	48
10	2 equiv. of formic acid in place of 4 equiv. of formic acid	30
11	7 equiv. of formic acid in place of 4 equiv. of formic acid	45
12	10 equiv. of formic acid in place of 4 equiv. of formic acid	44

[a]Reactions were carried out at 80 °C with **1a** (0.2 mmol), **2a** (2.5 equiv.), chitosan (20 mol%), formic acid (4.0 equiv.), H_2O (1.0 mL), Ar, 18 h, 120 °C. [b]Yield was determined by ^{1}H NMR analysis with CH_2Br_2 as internal standard. Chitosan (DD: 97.96%), chitin (DD: 2.5%).

Large-scale Synthesis and Catalytic Cycling Reactions.

Large-scale synthesis of 4

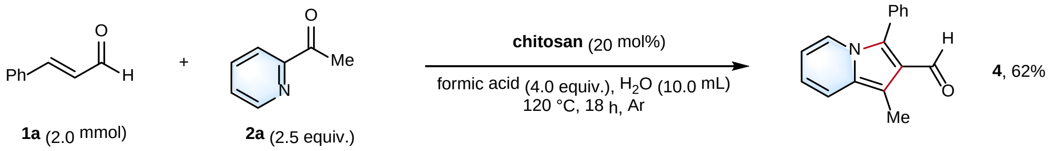

A mixture of **1a** (2.0 mmol), **2a** (2.5 equiv.), catalyst chitosan (0.4 mmol), formic acid (4.0 equiv.) in H_2O (10.0 mL) were stirred at 120 ºC under Ar atmosphere for 18 h. The reactions were conducted in a sealed Schlenk tube and heated by an IKA magnetic heating agitator with oil bath. The reaction temperature was directly read from temperature detector of IKA

apparatus and was calibrated by thermometer. After cooling to room temperature, the reaction mixture was extracted by diether (3×10 mL) and dried over anhydrous Na_2SO_4. After filtration and concentrated in rotary evaporator, the crude product was purified with flash chromatography on silica gel (ethyl acetate : *n*-hexane) to give product **4** (291.4 mg, 1.24 mmol, isolated yield 62%) as a yellow liquid.

Catalytic cycling experiments

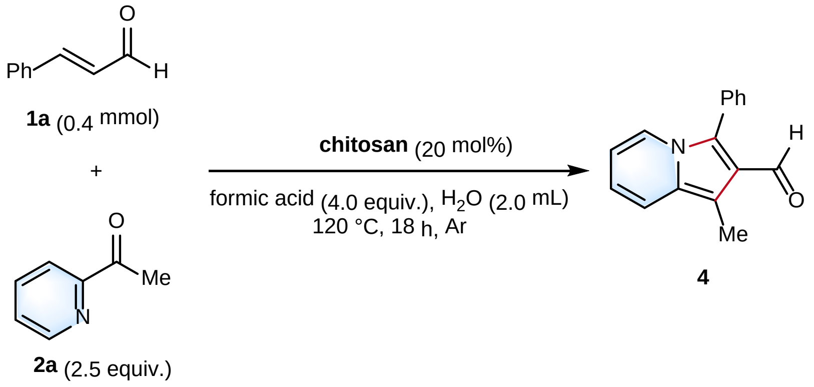

Catalytic cycle	Yield of 4
1	58%
2	57%
3	40%

The first catalytic cycle: A mixture of **1a** (0.4 mmol), **2a** (2.5 equiv.), catalyst chitosan (0.08 mmol), formic acid (4.0 equiv.) in H_2O (2.0 mL) were stirred at 120 ºC under Ar atmosphere for 18 h. The reactions were conducted in a sealed Schlenk tube and heated by an IKA magnetic heating agitator with heating block. The reaction temperature was directly read from temperature detector of IKA apparatus and was calibrated by thermometer. After cooling to room temperature, the reaction mixture was directly extracted by diether (3×3 mL) and dried over anhydrous Na_2SO_4. After filtration and concentrated in rotary evaporator, the crude product was purified with flash chromatography on silica gel (ethyl acetate : *n*-hexane) to give product **4** (54.1 mg, 0.23 mmol, isolated yield 58%) as a yellow liquid. The remaining aqueous phase was used for next catalytic cycle.

The second catalytic cycle: **1a** (0.4 mmol) and **2a** (2.5 equiv.) were directly added into the remaining aqueous phase of the first catalytic cycle and stirred at 120 ºC under Ar atmosphere for 18 h. The reactions were conducted in a sealed Schlenk tube and heated by an IKA magnetic heating agitator with heating block. The reaction temperature was directly read from temperature detector of IKA apparatus and was calibrated by thermometer. After cooling to room temperature, the reaction mixture was directly extracted by diether (3×3 mL) and dried over anhydrous Na_2SO_4. After filtration and concentrated in rotary evaporator, the

crude product was purified with flash chromatography on silica gel (ethyl acetate : *n*-hexane) to give product **4** (54.1 mg, 0.23 mmol, isolated yield 57%) as a yellow liquid. The remaining aqueous phase was used for next catalytic cycle.

The third catalytic cycle: **1a** (0.4 mmol) and **2a** (2.5 equiv.) were directly added into the remaining aqueous phase of the second catalytic cycle and stirred at 120 °C under Ar atmosphere for 18 h. The reactions were conducted in a sealed Schlenk tube and heated by an IKA magnetic heating agitator with heating block. The reaction temperature was directly read from temperature detector of IKA apparatus and was calibrated by thermometer. After cooling to room temperature, the reaction mixture was directly extracted by diether (3×3 mL) and dried over anhydrous Na_2SO_4. After filtration and concentrated in rotary evaporator, the crude product was purified with flash chromatography on silica gel (ethyl acetate : *n*-hexane) to give product **4** (37.6 mg, 0.16 mmol, isolated yield 40%) as a yellow liquid.

FT-IR analysis of catalyst chitosan after 3 recycling reactions

After the third recycling catalytic reaction followed the above procedures, the organic phase was extracted by diether. Then, the aqueous phase was basified to pH 7 *via* saturated Na_2CO_3 aqueous solution. Then, the precipitation chitosan was isolated by centrifuge and dried for FT-IR analysis.

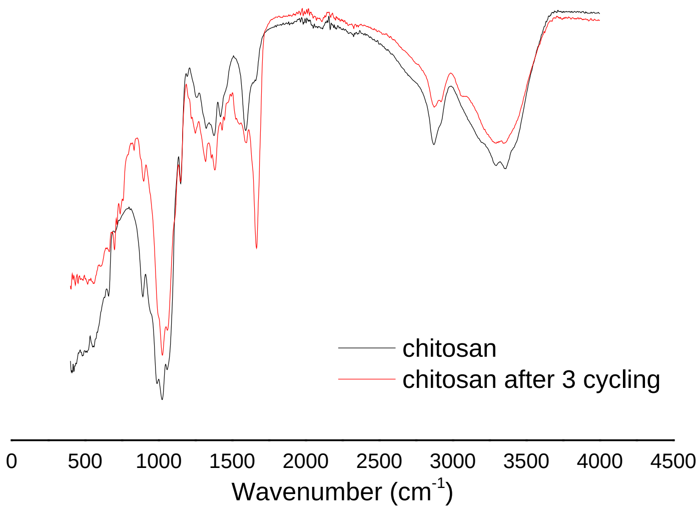

Supplementary Fig. 3 FT-IR analysis of catalyst chitosan after 3 recycling reactions.

Supplementary Method 7

Preparation of the Intermediates 3p and 3q

Preparation of intermediate 3p

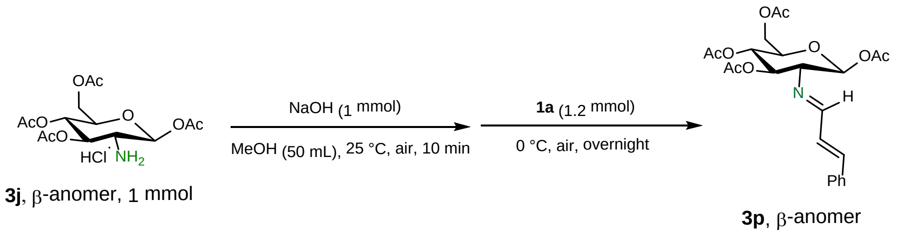

Preparation of **3p**: **3j** (1 mmol, 383.10 mg) was suspended in methanol (50 mL) at 25 ºC. NaOH (1 mmol, 40 mg) was added into the methanol solution to remove HCl. NaCl precipitate in methanol solution was filtered. Then, **1a** (1.2 mmol, 151.04 uL) was added into the solution, the reaction mixture was stirred in 0 ºC overnight. After the reaction, the solution was concentrated, filtered, dried in air, recrystallized from Methanol. Work-up gave product **3p** (161.4 mg, 0.35 mmol, isolated yield 35%) as a white solid; **mp**: 207−208 ºC. **FT-IR**: *v* (cm^{-1}): 2912, 1754, 1737, 1634, 1432, 1364, 1251, 1214, 1158, 1111, 1076, 1032, 1001, 903, 758, 694, 596, 564, 546, 497, 453, 412. **^{1}H-NMR** (400 MHz, DMSO-d_6) δ 8.10 (d, *J* = 8.8 Hz, 1H), 7.63 (d, *J* = 6.8 Hz, 2H), 7.42 – 7.36 (d, 3H), 7.22 (d, *J* = 16.0 Hz, 1H), 6.91 (dd, *J* = 16.0, 8.8 Hz, 1H), 5.99 (d, *J* = 8.0 Hz, 1H), 5.41 – 5.36 (m, 1H), 4.97 – 4.93 (m, 1H), 4.26 – 4.19 (m, 2H), 4.01 (d, *J* = 11.2 Hz, 1H), 3.39 (t, *J* = 9.0 Hz, 1H), 2.03 (s, 3H), 2.02 (s, 3H), 1.98 (s, 3H), 1.89 (s, 3H). **^{13}C NMR** (100 MHz, DMSO-d_6) δ 170.0, 169.4, 169.1, 168.6, 167.0, 143.6, 135.2, 129.6, 128.9, 127.6, 127.4, 92.5, 72.4, 72.2, 71.5, 67.8, 61.6, 20.5, 20.5, 20.4, 20.2. **ESI-HRMS**: *m/z* calcd. for $C_{23}H_{27}NO_9$ $[M+H]^+$: 462.1764, found 462.1763.

Preparation of intermediate 3q

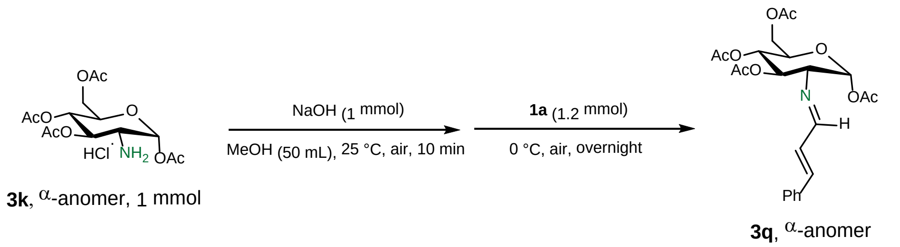

Preparation of **3q**: **3k** (1 mmol, 383.10 mg) was suspended in methanol (50 mL) at 25 ºC. NaOH (1 mmol, 40 mg) was added into the methanol solution to remove HCl. NaCl precipitate of methanol solution was filtered. Then, **1a** (1.2 mmol, 151.04 uL) was added into the solution, the reaction mixture was stirred in 0 ºC overnight. After the reaction, the

solution was concentrated, filtered, dried in air, recrystallized from Methanol. Work-up gave product **3q** (129.1 mg, 0.28 mmol, isolated yield 28%) as a white solid; **mp**: 174−175 ºC. **FT-IR**: *ν* (cm^{-1}): 2858, 1745, 1636, 1449, 1377, 1245, 1218, 1154, 1063, 938, 919, 878, 752, 690, 596, 536, 476, 437, 404. **^{1}H-NMR** (400 MHz, DMSO-d_6) δ 8.16 (d, *J* = 8.8 Hz, 1H), 7.65 – 7.63 (m, 2H), 7.42 – 7.36 (m, 3H), 7.19 (d, *J* = 16.0 Hz, 1H), 6.93 (dd, *J* = 16.0, 8.9 Hz, 1H), 6.06 (d, *J* = 3.6 Hz, 1H), 5.40 (t, *J* = 10.0 Hz, 1H), 5.04 (t, *J* = 9.6 Hz, 1H), 4.24 – 4.20 (m, 2H), 4.05 – 4.01 (m, 1H), 3.70 (dd, *J* = 10.3, 3.5 Hz, 1H), 2.17 (s, 3H), 2.02 (s, 3H), 1.99 (s, 3H), 1.89 (s, 3H). **^{13}C NMR** (100 MHz, DMSO-d_6) δ 170.0, 169.3, 169.3, 169.0, 167.7, 143.7, 135.2, 129.5, 128.9, 127.6, 127.5, 91.2, 70.6, 69.5, 69.3, 67.8, 61.5, 20.8, 20.5, 20.4, 20.3. **ESI-HRMS**: *m/z* calcd. for $C_{23}H_{27}NO_9$ $[M+H]^+$: 462.1764, found 462.1759.

HR-MS Analysis of Catalyst 3j After Catalytic Reactions

A mixture of **1a** (0.2 mmol), **2a** (2.5 equiv.), catalyst **3j** (0.04 mmol), AcOH (2.0 equiv.) and $LiSO_3CF_3$ (3.0 equiv.) in CF_3CH_2OH (0.9 mL) were stirred at 80 ºC under Ar atmosphere for 18 h. After the reaction, the crude reaction solution was directly test by HR-MS-ESI. The catalyst **3j** was detected in the below figure. **ESI-HRMS**: *m/z* calcd. for $C_{14}H_{22}NO_9^+$ [M]: 348.1289, found 348.1297.

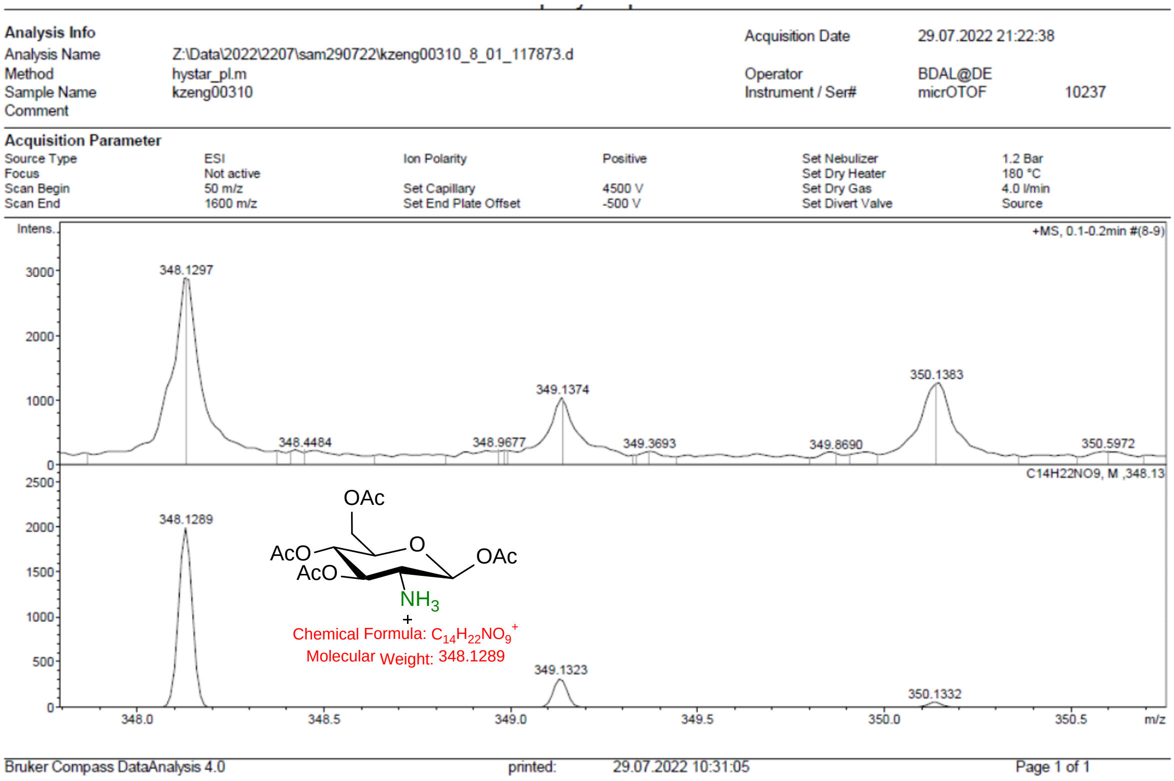

Supplementary Fig. 4 HR-MS analysis of catalyst **3j** after catalytic reactions

^{1}H NMR Analysis of the Conformation Stability of β-anomer

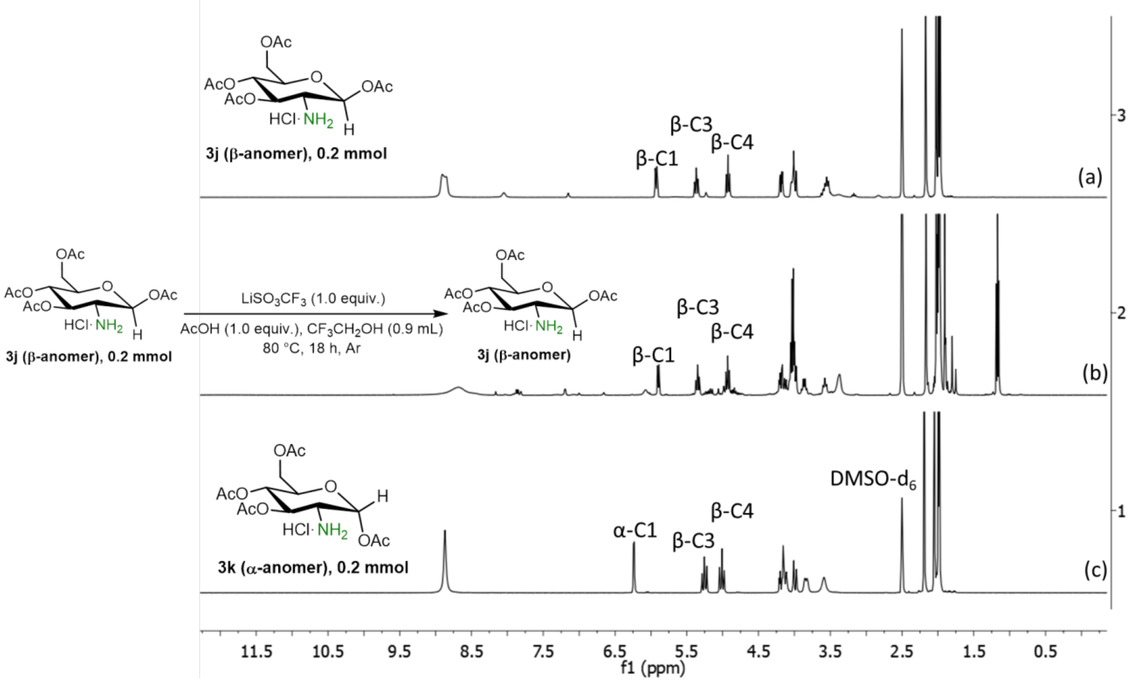

Supplementary Fig. 5 a) ^{1}H NMR analysis of **3j** (β anomer, 0.2 mmol) in DMSO-d_6 at r.t.; b) The mixture of **3j** (0.2 mmol), $LiSO_3CF_3$ (1.0 equiv.), and AcOH (1.0 equiv.) were stirred in CF_3CH_2OH (0.9 mL) at 80 °C for 18 h under Ar. After the concentration with rotary evaporator, the mixture was directly dissolved in DMSO-d6 and analyzed with ^{1}H NMR at r.t.; c) ^{1}H NMR analysis of **3k** (α anomer, 0.2 mmol) in DMSO-d_6 at r.t.

Supplementary Note 3

Characterization of Products

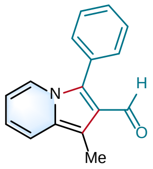

4

According to the general procedure A, a mixture of *α,β*-unsaturated aldehyde (0.2 mmol), heteroaryl ketone (2.5 equiv.), catalyst **3j** (0.04 mmol), AcOH (2.0 equiv.) and $LiSO_3CF_3$ (3.0 equiv.) in CF_3CH_2OH (0.9 mL) were stirred at 80 ºC under Ar atmosphere for 18 h. Work-up gave product 1-methyl-3-phenylindolizine-2-carbaldehyde (**4,** 44.7 mg, 0.19 mmol, isolated yield 95%) as a yellow liquid.

According to the general procedure E, a mixture of *α,β*-unsaturated aldehyde (0.2 mmol), heteroaryl ketone (2.5 equiv.), catalyst chitosan (0.04 mmol), formic acid (4.0 equiv.) in H_2O (1.0 mL) were stirred at 120 ºC under Ar atmosphere for 18 h. Work-up gave product 1-

methyl-3-phenylindolizine-2-carbaldehyde (**4,** 23.5 mg, 0.10 mmol, isolated yield 50%) as a yellow liquid.

FT-IR: *v* (cm^{-1}): 3052, 2922, 2749, 1661, 1517, 1477, 1430, 1383, 1358, 1319, 1247, 1218, 1142, 1115, 1076, 997, 940, 872, 830, 756, 736, 715, 699, 682, 666, 620, 534, 482, 431. **^{1}H NMR** (400 MHz, $CDCl_3$) δ 9.91 (s, 1H), 7.76 (d, *J* = 7.2 Hz, 1H), 7.47 – 7.45 (m, 2H), 7.42 – 7.40 (m, 3H), 7.32 (d, *J* = 9.2 Hz, 1H), 6.61 – 6.56 (m, 1H), 6.40 – 6.36 (m, 1H), 2.53 (s, 3H). **^{13}C NMR** (100 MHz, $CDCl_3$) δ 189.7, 131.3, 131.0, 130.6, 129.0, 128.9, 128.8, 123.4, 122.3, 119.1, 117.6, 112.7, 109.9, 9.7. **ESI-HRMS**: *m/z* calcd. for $C_{16}H_{13}NO$ $[M+H]^+$: 236.1075, found 236.1071.

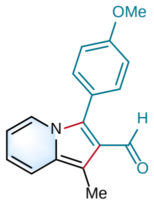

5

According to the general procedure A, a mixture of *α,β*-unsaturated aldehyde (0.2 mmol), heteroaryl ketone (2.5 equiv.), catalyst **3j** (0.04 mmol), AcOH (2.0 equiv.) and $LiSO_3CF_3$ (3.0 equiv.) in CF_3CH_2OH (0.9 mL) were stirred at 80 ºC under Ar atmosphere for 18 h. Work-up gave product 3-(4-methoxyphenyl)-1-methylindolizine-2-carbaldehyde (**5,** 33.4 mg, 0.13 mmol, isolated yield 63%) as a yellow liquid.

According to the general procedure E, a mixture of *α,β*-unsaturated aldehyde (0.2 mmol), heteroaryl ketone (2.5 equiv.), catalyst chitosan (0.04 mmol), formic acid (4.0 equiv.) in H_2O (1.0 mL) were stirred at 120 ºC under Ar atmosphere for 18 h. Work-up gave product 3-(4-methoxyphenyl)-1-methylindolizine-2-carbaldehyde (**5,** 21.7 mg, 0.08 mmol, isolated yield 41%) as a yellow liquid.

FT-IR: *v* (cm^{-1}): 2922, 2840, 2741, 1661, 1607, 1574, 1527, 1482, 1432, 1381, 1356, 1319, 1286, 1245, 1220, 1175, 1150, 1111, 1026, 878, 824, 785, 736, 684, 641, 626, 583, 515, 433, 404. **^{1}H NMR** (400 MHz, $CDCl_3$) δ 9.89 (s, 1H), 7.71 (d, *J* = 7.2 Hz, 1H), 7.34 – 7.28 (m, 3H), 6.98 (d, *J* = 8.8 Hz, 2H), 6.57 – 6.53 (m, 1H), 6.35 (t, *J* = 6.6 Hz, 1H), 3.81 (s, 3H), 2.52 (s, 3H). **^{13}C NMR** (100 MHz, $CDCl_3$) δ 189.7, 160.1, 132.2, 131.4, 130.3, 123.3, 122.3, 120.8, 119.0, 117.4, 114.5, 112.5, 109.6, 55.4, 9.6. **ESI-HRMS**: *m/z* calcd. for $C_{17}H_{15}NO_2$ $[M+H]^+$: 266.1181, found 266.1177.

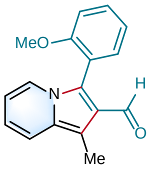

6

According to the general procedure A, a mixture of *α,β*-unsaturated aldehyde (0.2 mmol), heteroaryl ketone (2.5 equiv.), catalyst **3j** (0.04 mmol), AcOH (2.0 equiv.) and $LiSO_3CF_3$ (3.0 equiv.) in CF_3CH_2OH (0.9 mL) were stirred at 80 ºC under Ar atmosphere for 18 h. Work-up gave product 3-(2-methoxyphenyl)-1-methylindolizine-2-carbaldehyde (**6,** 50.4 mg, 0.19 mmol, isolated yield 95%) as a yellow liquid.

According to the general procedure E, a mixture of *α,β*-unsaturated aldehyde (0.2 mmol), heteroaryl ketone (2.5 equiv.), catalyst chitosan (0.04 mmol), formic acid (4.0 equiv.) in H_2O (1.0 mL) were stirred at 120 ºC under Ar atmosphere for 18 h. Work-up gave product 3-(2-methoxyphenyl)-1-methylindolizine-2-carbaldehyde (**6,** 28.1 mg, 0.11 mmol, isolated yield 53%) as a yellow liquid.

FT-IR: *ν* (cm^{-1}): 2920, 2833, 2745, 1663, 1601, 1576, 1515, 1463, 1432, 1383, 1358, 1321, 1288, 1278, 1245, 1216, 1181, 1152, 1146, 1127, 1100, 1047, 1022, 940, 880, 851, 835, 783, 736, 713, 684, 659, 573, 552, 528, 462, 429. **^{1}H NMR** (400 MHz, $CDCl_3$) δ 9.85 (s, 1H), 7.43 – 7.38 (m, 1H), 7.36 (d, J = 7.2 Hz, 1H), 7.31 (d, J = 9.2 Hz, 1H), 7.25 (dd, J = 7.2, 1.6 Hz, 1H), 7.03 – 6.98 (m, 2H), 6.57 (dd, J = 9.1, 6.3 Hz, 1H), 6.36 (t, J = 6.8 Hz, 1H), 3.70 (s, 3H), 2.54 (s, 3H). **^{13}C NMR** (100 MHz, $CDCl_3$) δ 189.7, 157.9, 133.7, 130.8, 128.2, 123.6, 123.4, 120.7, 118.8, 117.4, 117.2, 112.0, 111.2, 109.5, 55.5, 9.7. **ESI-HRMS**: *m/z* calcd. for $C_{17}H_{15}NO_2$ $[M+H]^+$: 266.1181, found 266.1176.

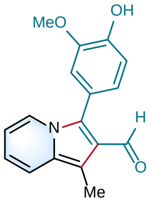

7

According to the general procedure A, a mixture of *α,β*-unsaturated aldehyde (0.2 mmol), heteroaryl ketone (2.5 equiv.), catalyst **3j** (0.04 mmol), AcOH (2.0 equiv.) and $LiSO_3CF_3$ (3.0

equiv.) in CF_3CH_2OH (0.9 mL) were stirred at 80 ºC under Ar atmosphere for 18 h. Work-up gave product 3-(4-hydroxy-3-methoxyphenyl)-1-methylindolizine-2-carbaldehyde (**7**, 36.5 mg, 0.13 mmol, isolated yield 63%) as a yellow solid.

According to the general procedure E, a mixture of *α*,*β*-unsaturated aldehyde (0.2 mmol), heteroaryl ketone (2.5 equiv.), catalyst chitosan (0.04 mmol), formic acid (4.0 equiv.) in H_2O (1.0 mL) were stirred at 120 ºC under Ar atmosphere for 18 h. Work-up gave product 3-(4-hydroxy-3-methoxyphenyl)-1-methylindolizine-2-carbaldehyde (**7**, 40.5 mg, 0.14 mmol, isolated yield 72%) as a yellow solid.

mp: 168−169 ˚C. **FT-IR**: *ν* (cm^{-1}): 3171, 2924, 2852, 1638, 1583, 1523, 1488, 1471, 1432, 1416, 1377, 1344, 1319, 1271, 1236, 1208, 1177, 1142, 1125, 1057, 1030, 964, 913, 888, 876, 812, 769, 736, 725, 684, 659, 647, 571, 558, 528, 517, 433, 410. **^{1}H NMR** (400 MHz, $CDCl_3$) δ 9.92 (s, 1H), 7.74 (d, *J* = 6.8 Hz, 1H), 7.30 (d, *J* = 8.8 Hz, 1H), 7.00 (d, *J* = 8.0 Hz, 1H), 6.93 (d, *J* = 8.0 Hz, 1H), 6.86 (s, 1H), 6.58 – 6.54 (m, 1H), 6.37 (t, *J* = 6.8 Hz, 1H), 5.83 (s, 1H), 3.84 (s, 3H), 2.52 (s, 3H). **^{13}C NMR** (100 MHz, $CDCl_3$) δ 189.8, 146.9, 146.5, 131.6, 130.3, 124.4, 123.3, 122.5, 120.5, 119.1, 117.4, 115.0, 113.4, 112.6, 109.6, 56.1, 9.6. **ESI-HRMS**: *m/z* calcd. for $C_{17}H_{15}NO_3$ $[M+H]^+$: 282.1130, found 282.1124.

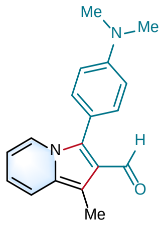

8

According to the general procedure B, a mixture of *α*,*β*-unsaturated aldehyde (0.2 mmol), heteroaryl ketone (2.5 equiv.), catalyst **3j** (0.04 mmol), and $LiSO_3CF_3$ (3.0 equiv.) in AcOH : CF_3CH_2OH (0.4 : 0.5 mL) were stirred at 80 ºC under Ar atmosphere for 36 h. Work-up gave product 3-(4-(dimethylamino)phenyl)-1-methylindolizine-2-carbaldehyde (**8**, 25.0 mg, 0.09 mmol, isolated yield 46%) as a yellow liquid.

According to the general procedure F, a mixture of *α*,*β*-unsaturated aldehyde (0.2 mmol), heteroaryl ketone (2.5 equiv.) and catalyst chitosan (0.04 mmol) (4.0 equiv.) in H_2O : formic acid (0.5 : 0.5 mL) were stirred at 120 ºC under Ar atmosphere for 36 h. Work-up gave

product 3-(4-(dimethylamino)phenyl)-1-methylindolizine-2-carbaldehyde (**8**, 19.5 mg, 0.07 mmol, isolated yield 36%) as a yellow liquid.

FT-IR: *ν* (cm^{-1}): 2924, 2854, 2800, 1661, 1605, 1531, 1488, 1432, 1352, 1222, 1195, 1164, 1113, 1057, 944, 876, 814, 736, 641, 552, 515, 435. **^{1}H NMR** (400 MHz, $CDCl_3$) δ 9.91 (s, 1H), 7.78 (d, *J* = 7.6 Hz, 1H), 7.30 – 7.25 (m, 4H), 6.77 (d, *J* = 8.6 Hz, 2H), 6.53 (dd, *J* = 9.0, 6.4 Hz, 1H), 6.33 (t, *J* = 6.6 Hz, 1H), 2.98 (s, 6H), 2.52 (s, 3H). **^{13}C NMR** (100 MHz, $CDCl_3$) δ 190.2, 150.6, 132.8, 131.8, 130.1, 123.1, 122.6, 119.0, 117.2, 115.7, 112.2, 112.2, 109.3, 40.3, 9.7. **ESI-HRMS**: m/z calcd. for $C_{18}H_{18}N_2O$ [M+H]+: 279.1497, found 279.1502.

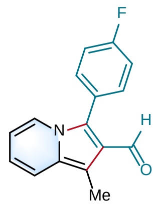

9

According to the general procedure A, a mixture of *α,β*-unsaturated aldehyde (0.2 mmol), heteroaryl ketone (2.5 equiv.), catalyst **3j** (0.04 mmol), AcOH (2.0 equiv.) and $LiSO_3CF_3$ (3.0 equiv.) in CF_3CH_2OH (0.9 mL) were stirred at 80 ºC under Ar atmosphere for 18 h. Work-up gave product 3-(4-fluorophenyl)-1-methylindolizine-2-carbaldehyde (**9**, 40.5 mg, 0.16 mmol, isolated yield 81%) as a yellow liquid.

According to the general procedure E, a mixture of *α,β*-unsaturated aldehyde (0.2 mmol), heteroaryl ketone (2.5 equiv.), catalyst chitosan (0.04 mmol), formic acid (4.0 equiv.) in H_2O (1.0 mL) were stirred at 120 ºC under Ar atmosphere for 18 h. Work-up gave product 3-(4-fluorophenyl)-1-methylindolizine-2-carbaldehyde (**9**, 22.8 mg, 0.09 mmol, isolated yield 45%) as a yellow liquid.

FT-IR: *ν* (cm^{-1}): 3072, 2924, 2739, 1665, 1601, 1525, 1480, 1434, 1383, 1356, 1319, 1220, 1158, 1113, 1094, 1053, 938, 878, 828, 802, 736, 717, 684, 641, 571, 550, 509, 441, 420. **^{1}H NMR** (400 MHz, $CDCl_3$) δ 9.88 (s, 1H), 7.67 (d, *J* = 7.2 Hz, 1H), 7.39 – 7.36 (m, 2H), 7.31 (d, *J* = 9.2 Hz, 1H), 7.15 (t, *J* = 8.6 Hz, 2H), 6.57 (dd, *J* = 9.2, 6.4 Hz, 1H), 6.40 – 6.37 (m, 1H), 2.51 (s, 3H). **^{13}C NMR** (100 MHz, $CDCl_3$) δ 189.2, 163.0 (d, $^1J_{C\text{-}F}$ = 248.2 Hz), 132.8 (d, $^3J_{C\text{-}F}$ = 8.3 Hz), 130.6, 129.8, 124.9 (d, $^4J_{C\text{-}F}$ = 3.5 Hz), 123.4, 122.1, 119.1, 117.6, 116.2 (d, $^2J_{C\text{-}F}$ = 21.6 Hz), 112.9, 110.0, 9.5. **^{19}F NMR** (375 MHz, $CDCl_3$) δ -111.5. **ESI-HRMS**: *m/z* calcd. for $C_{16}H_{12}NOF$ $[M+H]^+$: 254.0981, found 254.0975.

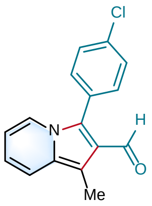

10

According to the general procedure A, a mixture of *α,β*-unsaturated aldehyde (0.2 mmol), heteroaryl ketone (2.5 equiv.), catalyst **3j** (0.04 mmol), AcOH (2.0 equiv.) and $LiSO_3CF_3$ (3.0 equiv.) in CF_3CH_2OH (0.9 mL) were stirred at 80 ºC under Ar atmosphere for 18 h. Work-up gave product 3-(4-chlorophenyl)-1-methylindolizine-2-carbaldehyde (**10**, 40.4 mg, 0.15 mmol, isolated yield 75%) as a yellow solid.

According to the general procedure E, a mixture of *α,β*-unsaturated aldehyde (0.2 mmol), heteroaryl ketone (2.5 equiv.), catalyst chitosan (0.04 mmol), formic acid (4.0 equiv.) in H_2O (1.0 mL) were stirred at 120 ºC under Ar atmosphere for 18 h. Work-up gave product 3-(4-chlorophenyl)-1-methylindolizine-2-carbaldehyde (**10**, 16.1 mg, 0.06 mmol, isolated yield 30%) as a yellow solid.

mp: 105−106 ˚C. **FT-IR**: *v* (cm^{-1}): 3056, 2916, 2846, 1655, 1510, 1475, 1430, 1405, 1385, 1352, 1319, 1247, 1220, 1150, 1111, 1090, 1014, 938, 878, 820, 740, 729, 711, 680, 637, 622, 548, 536, 501, 482, 435, 404. **^{1}H NMR** (400 MHz, $CDCl_3$) δ 9.90 (s, 1H), 7.71 (d, *J* = 7.2 Hz, 1H), 7.44 (d, *J* = 8.4 Hz, 2H), 7.35 – 7.31 (m, 3H), 6.61 – 6.57 (m, 1H), 6.40 (t, *J* = 6.8 Hz, 1H), 2.52 (s, 3H). **^{13}C NMR** (100 MHz, $CDCl_3$) δ 189.1, 135.1, 132.1, 130.8, 129.4, 127.3, 123.4, 122.0, 119.2, 117.7, 113.1, 110.3, 9.5. **ESI-HRMS**: *m/z* calcd. for $C_{16}H_{13}ClNO$ $[M+H]^+$: 270.0686, found 270.0685.

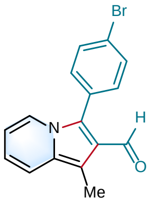

11

According to the general procedure A, a mixture of *α,β*-unsaturated aldehyde (0.2 mmol), heteroaryl ketone (2.5 equiv.), catalyst **3j** (0.04 mmol), AcOH (2.0 equiv.) and $LiSO_3CF_3$ (3.0 equiv.) in CF_3CH_2OH (0.9 mL) were stirred at 80 ºC under Ar atmosphere for 18 h. Work-up

gave product 3-(4-bromophenyl)-1-methylindolizine-2-carbaldehyde (**11**, 53.2 mg, 0.17 mmol, isolated yield 87%) as a yellow liquid. **FT-IR**: *ν* (cm^{-1}): 2916, 2747, 1663, 1587, 1508, 1471, 1432, 1399, 1381, 1354, 1321, 1249, 1220, 1148, 1115, 1069, 1010, 938, 878, 814, 736, 680, 666, 622, 560, 534, 492, 433, 402. **^{1}H NMR** (400 MHz, $CDCl_3$) δ 9.89 (s, 1H), 7.71 (d, J = 7.6 Hz, 1H), 7.58 (d, J = 8.4 Hz, 2H), 7.31 (d, J = 9.2 Hz, 1H), 7.26 (d, J = 8.4 Hz, 2H), 6.58 (dd, J = 9.2, 6.6 Hz, 1H), 6.41 – 6.37 (m, 1H), 2.50 (s, 3H). **^{13}C NMR** (100 MHz, $CDCl_3$) δ 189.0, 132.3, 132.3, 130.8, 129.3, 127.8, 123.3, 123.2, 122.0, 119.1, 117.7, 113.1, 110.3, 9.5. **ESI-HRMS**: *m/z* calcd. for $C_{16}H_{12}NOBr$ $[M+H]^+$: 314.0181, found 314.0164.

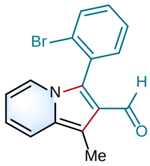

12

According to the general procedure A, a mixture of *α,β*-unsaturated aldehyde (0.2 mmol), heteroaryl ketone (2.5 equiv.), catalyst **3j** (0.04 mmol), AcOH (2.0 equiv.) and $LiSO_3CF_3$ (3.0 equiv.) in CF_3CH_2OH (0.9 mL) were stirred at 80 ºC under Ar atmosphere for 18 h. Work-up gave product 3-(2-bromophenyl)-1-methylindolizine-2-carbaldehyde (**12**, 43.8 mg, 0.14 mmol, isolated yield 71%) as a yellow liquid.

According to the general procedure E, a mixture of *α,β*-unsaturated aldehyde (0.2 mmol), heteroaryl ketone (2.5 equiv.), catalyst chitosan (0.04 mmol), formic acid (4.0 equiv.) in H_2O (1.0 mL) were stirred at 120 ºC under Ar atmosphere for 18 h. Work-up gave product 3-(2-bromophenyl)-1-methylindolizine-2-carbaldehyde (**12**, 15.0 mg, 0.05 mmol, isolated yield 24%) as a yellow liquid.

FT-IR: *ν* (cm^{-1}): 2918, 2850, 2807, 2739, 1667, 1510, 1432, 1385, 1358, 1321, 1249, 1218, 1154, 1144, 1125, 1115, 1045, 1024, 954, 882, 861, 833, 754, 736, 711, 688, 645, 569, 550, 536, 497, 447, 420. **^{1}H NMR** (400 MHz, $CDCl_3$) δ 9.81 (s, 1H), 7.69 – 7.67 (m, 1H), 7.38 – 7.27 (m, 5H), 6.61 (dd, *J* = 8.8, 6.4 Hz, 1H), 6.44 – 6.40 (m, 1H), 2.54 (s, 3H). **^{13}C NMR** (100 MHz, $CDCl_3$) δ 188.8, 134.2, 133.3, 131.0, 130.7, 130.3, 129.2, 127.6, 125.9, 123.6, 122.8, 119.0, 117.6, 112.7, 109.6, 9.6. **ESI-HRMS**: *m/z* calcd. for $C_{16}H_{12}NOBr$ $[M+H]^+$: 314.0181, found 314.0164.

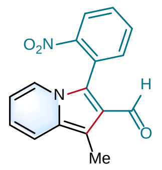

13

According to the general procedure A, a mixture of *α,β*-unsaturated aldehyde (0.2 mmol), heteroaryl ketone (2.5 equiv.), catalyst **3j** (0.04 mmol), AcOH (2.0 equiv.) and $LiSO_3CF_3$ (3.0 equiv.) in CF_3CH_2OH (0.9 mL) were stirred at 80 ºC under Ar atmosphere for 18 h. Work-up gave product 1-methyl-3-(2-nitrophenyl)indolizine-2-carbaldehyde (**13**, 39.2 mg, 0.14 mmol, isolated yield 69%) as a red liquid. **FT-IR**: *ν* (cm^{-1}): 2916, 2854, 1667, 1609, 1583, 1523, 1467, 1432, 1385, 1339, 1300, 1249, 1220, 1144, 995, 950, 936, 853, 830, 787, 738, 717, 701, 666, 647, 567, 536, 482, 422. **^{1}H NMR** (400 MHz, $CDCl_3$) δ 9.87 (s, 1H), 8.15 (dd, *J* = 8.0, 1.2 Hz, 1H), 7.68 (td, *J* = 7.2, 1.6 Hz, 1H), 7.63 – 7.59 (m, 1H), 7.44 (dd, *J* = 7.6, 1.2 Hz, 1H), 7.36 (d, *J* = 9.2 Hz, 1H), 7.28 (d, *J* = 7.2 Hz, 1H), 6.63 (dd, *J* = 9.2, 6.4 Hz, 1H), 6.44 – 6.41 (m, 1H), 2.53 (s, 3H). **^{13}C NMR** (100 MHz, $CDCl_3$) δ 187.7, 149.7, 134.5, 133.4, 131.3, 130.5, 125.2, 124.5, 123.5, 123.4, 122.2, 119.2, 117.7, 113.6, 111.0, 9.1. **ESI-HRMS**: *m/z* calcd. for $C_{16}H_{12}N_2O_3$ $[M+H]^+$: 281.0926, found 281.0921.

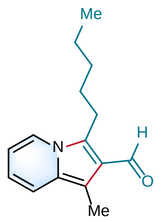

14

According to the general procedure A, a mixture of *α,β*-unsaturated aldehyde (0.2 mmol), heteroaryl ketone (2.5 equiv.), catalyst **3j** (0.04 mmol), AcOH (2.0 equiv.) and $LiSO_3CF_3$ (3.0 equiv.) in CF_3CH_2OH (0.9 mL) were stirred at 80 ºC under Ar atmosphere for 18 h. Work-up gave product 1-methyl-3-pentylindolizine-2-carbaldehyde (**14**, 34.4 mg, 0.15 mmol, isolated yield 76%) as a yellow liquid. **FT-IR**: *ν* (cm^{-1}): 2955, 2924, 2856, 2734, 1663, 1504, 1447, 1434, 1393, 1321, 1249, 1199, 1142, 1111, 1057, 905, 853, 732, 643, 523, 427. **^{1}H NMR** (400 MHz, $CDCl_3$) δ 10.22 (s, 1H), 7.57 (d, *J* = 7.2 Hz, 1H), 7.23 (d, *J* = 9.2 Hz, 1H), 6.48 (dd, *J* = 8.8, 6.4 Hz, 1H), 6.44 – 6.40 (m, 1H), 3.08 – 3.05 (m, 2H), 2.44 (s, 3H), 1.55 (p, *J* = 7.4 Hz, 2H), 1.29 – 1.25 (m, 4H), 0.80 (s, 3H). **^{13}C NMR** (100 MHz, $CDCl_3$) δ 188.0, 130.3,

129.7, 122.0, 121.6, 119.1, 115.9, 112.6, 109.8, 31.5, 27.8, 23.6, 22.4, 13.9, 8.9. **ESI-HRMS**: *m/z* calcd. for $C_{15}H_{19}NO$ $[M+H]^+$: 230.1545, found 230.1542.

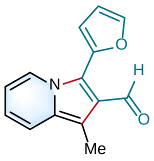

15

According to the general procedure C, a mixture of *α,β*-unsaturated aldehyde (0.2 mmol), heteroaryl ketone (2.5 equiv.), catalyst **3j** (0.04 mmol), AcOH (4.0 equiv.) and $LiSO_3CF_3$ (3.0 equiv.) in CF_3CH_2OH (0.9 mL) were stirred at room temperature under Ar atmosphere for 42 h. Work-up gave product 3-(furan-2-yl)-1-methylindolizine-2-carbaldehyde (**15**, 13.5 mg, 0.06 mmol, isolated yield 30%) as a yellow liquid. **FT-IR**: *ν* (cm^{-1}): 3118, 2920, 2850, 1663, 1510, 1463, 1434, 1381, 1352, 1319, 1249, 1212, 1162, 1144, 1117, 1076, 1016, 958, 888, 874, 808, 7734, 682, 659, 620, 593, 528, 427. **^{1}H NMR** (400 MHz, $CDCl_3$) δ 10.17 (s, 1H), 8.13 (d, *J* = 7.2 Hz, 1H), 7.58 (s, 1H), 7.35 (d, *J* = 9.2 Hz, 1H), 6.68 – 6.64 (m, 1H), 6.61 (d, *J* = 3.2 Hz, 1H), 6.55 – 6.50 (m, 2H), 2.52 (s, 3H). **^{13}C NMR** (100 MHz, $CDCl_3$) δ 189.3, 143.6, 143.4, 131.5, 124.0, 124.0, 120.2, 119.0, 118.2, 113.3, 112.2, 111.6, 110.9, 9.6. **ESI-HRMS**: *m/z* calcd. for $C_{14}H_{11}NO_2$ $[M+H]^+$: 226.0868, found 226.0862.

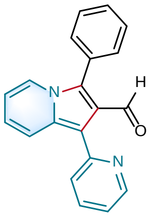

16

According to the general procedure A, a mixture of *α,β*-unsaturated aldehyde (0.2 mmol), heteroaryl ketone (2.5 equiv.), catalyst **3j** (0.04 mmol), AcOH (2.0 equiv.) and $LiSO_3CF_3$ (3.0 equiv.) in CF_3CH_2OH (0.9 mL) were stirred at 80 ºC under Ar atmosphere for 18 h. Work-up gave product 3-phenyl-1-(pyridin-2-yl)indolizine-2-carbaldehyde (**16**, 56.6 mg, 0.19 mmol, isolated yield 95%) as a yellow liquid. **FT-IR**: *ν* (cm^{-1}): 3060, 2846, 2761, 1673, 1585, 1517, 1473, 1445, 1420, 1381, 1354, 1323, 1278, 1267, 1236, 1191, 1146, 1123, 1096, 1076, 1055, 1039, 1024, 987, 948, 923, 905, 824, 779, 740, 719, 694, 664, 645, 624, 610, 585, 565, 507,

486, 433, 406. **^{1}H NMR** (400 MHz, $CDCl_3$) δ 10.01 (s, 1H), 8.61 (d, *J* = 4.8 Hz, 1H), 7.86 (d, *J* = 9.2 Hz, 1H), 7.77 (d, *J* = 7.2 Hz, 1H), 7.65 (d, *J* = 3.2 Hz, 2H), 7.47 – 7.40 (m, 5H), 7.09 (q, *J* = 4.4 Hz, 1H), 6.74 (dd, *J* = 9.2, 6.5 Hz, 1H), 6.47 (t, *J* = 6.8 Hz, 1H). **^{13}C NMR** (100 MHz, $CDCl_3$) δ 188.6, 153.2, 149.1, 135.7, 132.1, 131.6, 131.1, 129.2, 128.9, 128.7, 125.5, 122.7, 122.2, 120.9, 120.4, 114.0, 113.6. **ESI-HRMS**: *m/z* calcd. for $C_{20}H_{14}N_2O$ $[M+H]^+$: 299.1184, found 299.1180.

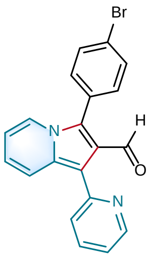

17

According to the general procedure A, a mixture of *α,β*-unsaturated aldehyde (0.2 mmol), heteroaryl ketone (2.5 equiv.), catalyst **3j** (0.04 mmol), AcOH (2.0 equiv.) and $LiSO_3CF_3$ (3.0 equiv.) in CF_3CH_2OH (0.9 mL) were stirred at 80 ºC under Ar atmosphere for 18 h. Work-up gave product 3-(4-bromophenyl)-1-(pyridin-2-yl)indolizine-2-carbaldehyde (**17**, 63.9 mg, 0.17 mmol, isolated yield 83%) as a yellow solid. **mp**: 167−168 ºC. **FT-IR**: *ν* (cm^{-1}): 3052, 2854, 2770, 1671, 1630, 1585, 1562, 1519, 1506, 1469, 1434, 1397, 1333, 1263, 1232, 1193, 1148, 1107, 1094, 1074, 1051, 1037, 1008, 989, 946, 911, 841, 814, 775, 740, 725, 713, 686, 618, 563, 503, 495, 441, 404. **^{1}H-NMR** (400 MHz, $CDCl_3$) δ 10.07 (s, 1H), 8.66 (d, *J* = 3.6 Hz, 1H), 7.86 (d, *J* = 9.2 Hz, 1H), 7.80 – 7.72 (m, 2H), 7.65 – 7.63 (m, 3H), 7.36 (d, *J* = 8.1 Hz, 2H), 7.19 (s, 1H), 6.83 (*t*, *J* = 7.6 Hz, 1H), 6.57 (*t*, *J* = 6.5 Hz, 1H). **^{13}C NMR** (100 MHz, $CDCl_3$) δ 188.5, 152.8, 149.3, 136.1, 132.7, 132.3, 132.2, 129.1, 127.9, 125.4, 123.7, 122.6, 122.5, 121.1, 121.1, 120.4, 114.1. **ESI-HRMS**: *m/z* calcd. for $C_{20}H_{13}BrN_2O$ $[M+H]^+$: 377.0290, found 377.0280.

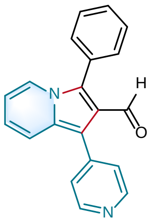

18

According to the general procedure A, a mixture of *α*,*β*-unsaturated aldehyde (0.2 mmol), heteroaryl ketone (2.5 equiv.), catalyst **3j** (0.04 mmol), AcOH (2.0 equiv.) and $LiSO_3CF_3$ (3.0 equiv.) in CF_3CH_2OH (0.9 mL) were stirred at 80 °C under Ar atmosphere for 18 h. Work-up gave product 3-phenyl-1-(pyridin-4-yl)indolizine-2-carbaldehyde (**18**, 50.7 mg, 0.17 mmol, isolated yield 85%) as a yellow solid. **mp**: 182−183 °C. **FT-IR**: *ν* (cm^{-1}): 3050, 2106, 1657, 1628, 1593, 1554, 1533, 1490, 1473, 1436, 1352, 1331, 1300, 1245, 1142, 1119, 1076, 1057, 1006, 950, 863, 824, 769, 756, 723, 711, 696, 686, 668, 635, 622, 612, 546, 499, 443, 414. **^{1}H NMR** (400 MHz, $CDCl_3$) δ 9.91 (s, 1H), 8.58 (d, *J* = 6.0 Hz, 2H), 7.84 (d, *J* = 7.2 Hz, 1H), 7.52 – 7.45 (m, 6H), 7.40 (d, *J* = 6.0 Hz, 2H), 6.76 (dd, *J* = 9.2, 6.4 Hz, 1H), 6.53 (t, *J* = 6.8 Hz, 1H). **^{13}C NMR** (100 MHz, $CDCl_3$) δ 188.0, 149.5, 141.6, 132.7, 131.3, 131.1, 129.5, 129.1, 128.1, 125.2, 123.0, 121.9, 121.1, 118.9, 113.7, 111.9. **ESI-HRMS**: *m/z* calcd. for $C_{20}H_{14}N_2O$ $[M+H]^+$: 299.1184, found 299.1179.

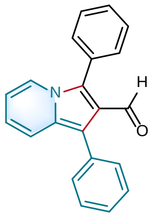

19

According to the general procedure A, a mixture of *α*,*β*-unsaturated aldehyde (0.2 mmol), heteroaryl ketone (2.5 equiv.), catalyst **3j** (0.04 mmol), AcOH (2.0 equiv.) and $LiSO_3CF_3$ (3.0 equiv.) in CF_3CH_2OH (0.9 mL) were stirred at 80 °C under Ar atmosphere for 18 h. Work-up gave product 1,3-diphenylindolizine-2-carbaldehyde (**19**, 50.5 mg, 0.17 mmol, isolated yield 84%) as a yellow solid.[6] **mp**: 164−165 °C. **FT-IR**: *ν* (cm^{-1}): 3029, 1675, 1591, 1525, 1475, 1442, 1428, 1414, 1383, 1354, 1344, 1331, 1315, 1261, 1236, 1218, 1187, 1146, 1076, 1039, 1022, 989, 948, 925, 905, 843, 822, 804, 750, 740, 729, 711, 692, 674, 633, 604, 585, 569, 511, 488, 435, 414. **^{1}H NMR** (400 MHz, $CDCl_3$) δ 9.95 (s, 1H), 7.79 (d, *J* = 7.2 Hz, 1H), 7.46 – 7.43 (m, 7H), 7.40 – 7.35 (m, 3H), 7.26 (t, *J* = 7.2 Hz, 1H), 6.62 (dd, *J* = 9.2, 6.4 Hz, 1H), 6.43 (t, *J* = 6.8 Hz, 1H). **^{13}C NMR** (100 MHz, $CDCl_3$) δ 188.4, 133.1, 131.0, 130.7, 130.6, 130.2, 129.1, 129.0, 128.9, 128.2, 126.8, 122.6, 122.0, 119.6, 119.4, 116.2, 113.5. **ESI-HRMS**: *m/z* calcd. for $C_{21}H_{15}NO$ $[M+H]^+$: 298.1232, found 298.1227.

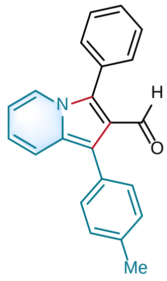

20

According to the general procedure A, a mixture of *α,β*-unsaturated aldehyde (0.2 mmol), heteroaryl ketone (2.5 equiv.), catalyst **3j** (0.04 mmol), AcOH (2.0 equiv.) and $LiSO_3CF_3$ (3.0 equiv.) in CF_3CH_2OH (0.9 mL) were stirred at 80 ºC under Ar atmosphere for 18 h. Work-up gave product 3-phenyl-1-(p-tolyl)indolizine-2-carbaldehyde (**20**, 52.9 mg, 0.17 mmol, isolated yield 87%) as a yellow liquid. **FT-IR**: *v* (cm^{-1}): 3019, 2918, 2848, 2747, 1673, 1626, 1601, 1523, 1504, 1475, 1447, 1428, 1381, 1356, 1323, 1259, 1232, 1183, 1121, 1074, 1041, 1016, 971, 950, 923, 903, 812, 746, 729, 696, 565, 507, 441. **^{1}H NMR** (400 MHz, $CDCl_3$) δ 9.93 (s, 1H), 7.76 (d, *J* = 7.2 Hz, 2H), 7.43-7.42 (m, 4H), 7.38 (d, J = 2.4 Hz, 1H), 7.34 – 7.30 (m, 3H), 7.17 (d, J = 8.0 Hz, 2H), 6.58 (ddd, J = 9.2, 6.4, 1.2 Hz, 1H), 6.42-6.38 (m, 1H). **^{13}C NMR** (100 MHz, $CDCl_3$) δ 188.5, 136.4, 131.2, 130.9, 130.4, 130.0, 129.0, 129.0, 129.0, 128.9, 128.4, 122.5, 122.0, 119.7, 119.2, 116.2, 113.4, 21.2. **ESI-HRMS**: *m/z* calcd. for $C_{22}H_{17}NO$ $[M+H]^+$: 312.1388, found 312.1384.

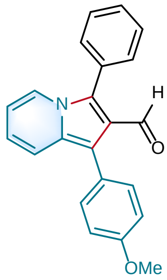

21

According to the general procedure A, a mixture of *α,β*-unsaturated aldehyde (0.2 mmol), heteroaryl ketone (2.5 equiv.), catalyst **3j** (0.04 mmol), AcOH (2.0 equiv.) and $LiSO_3CF_3$ (3.0 equiv.) in CF_3CH_2OH (0.9 mL) were stirred at 80 ºC under Ar atmosphere for 18 h. Work-up gave product 1-(4-methoxyphenyl)-3-phenylindolizine-2-carbaldehyde (**21**, 22.9 mg, 0.07 mmol, isolated yield 35%) as a yellow liquid. **FT-IR**: *v* (cm^{-1}): 3015, 2930, 2833, 2749, 1673, 1605, 1537, 1523, 1504, 1463, 1445, 1428, 1381, 1358, 1286, 1243, 1175, 1109, 1076, 1030, 1020, 948, 923, 903, 833, 785, 748, 729, 696, 666, 565, 528, 488, 439, 410. **^{1}H NMR** (400

MHz, $CDCl_3$) δ 9.94 (s, 1H), 7.79 (d, *J* = 7.2 Hz, 1H), 7.47 – 7.44 (m, 4H), 7.42 – 7.37 (m, 4H), 6.93 (d, *J* = 8.4 Hz, 2H), 6.63 – 6.59 (m, 1H), 6.45 – 6.41 (m, 1H), 3.79 (s, 3H). **^{13}C NMR** (100 MHz, $CDCl_3$) δ 188.6, 158.6, 131.7, 131.0, 130.6, 130.2, 129.0, 129.0, 128.9, 125.3, 122.5, 122.0, 119.7, 119.1, 115.9, 113.7, 113.4, 55.3. **ESI-HRMS**: *m/z* calcd. for $C_{22}H_{17}NO_2$ $[M+H]^+$: 328.1338, found 328.1331.

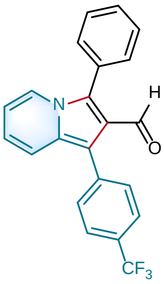

22

According to the general procedure A, a mixture of *α,β*-unsaturated aldehyde (0.2 mmol), heteroaryl ketone (2.5 equiv.), catalyst **3j** (0.04 mmol), AcOH (2.0 equiv.) and $LiSO_3CF_3$ (3.0 equiv.) in CF_3CH_2OH (0.9 mL) were stirred at 80 ºC under Ar atmosphere for 18 h. Work-up gave product 3-phenyl-1-(4-(trifluoromethyl)phenyl)indolizine-2-carbaldehyde (**22**, 69.4 mg, 0.19 mmol, isolated yield 97%) as a yellow solid. **mp**: 127−128 ºC. **FT-IR**: *ν* (cm^{-1}): 3058, 2848, 2776, 1669, 1613, 1539, 1523, 1445, 1426, 1409, 1383, 1354, 1317, 1261, 1230, 1160, 1107, 1065, 1016, 939, 903, 843, 833, 756, 736, 703, 692, 674, 600, 558, 490, 455, 439. **^{1}H NMR** (400 MHz, $CDCl_3$) δ 9.91 (s, 1H), 7.83 (d, J = 7.2 Hz, 1H), 7.60 (q, J = 8.4 Hz, 4H), 7.52 – 7.45 (m, 5H), 7.40 (d, J = 9.2 Hz, 1H), 6.71 (dd, J = 9.2, 6.4 Hz, 1H), 6.50 (t, J = 6.8 Hz, 1H). **^{13}C NMR** (100 MHz, $CDCl_3$) δ 188.2, 148.6, 137.2 (q, $^4J_{C-F}$ = 1.2 Hz), 132.2, 131.1, 130.7, 129.4, 129.1, 128.9 (q, $^2J_{C-F}$ = 32.2 Hz), 128.4, 126.7, 125.0 (q, $^3J_{C-F}$ = 3.7 Hz), 124.4 (q, $^1J_{C-F}$ = 270.2 Hz), 122.9, 122.0, 120.5, 119.1, 113.6. **^{19}F NMR** (377 MHz, $CDCl_3$) δ -62.3. **ESI-HRMS**: *m/z* calcd. for $C_{22}H_{14}F_3NO$ $[M+H]^+$: 366.1106, found 366.1100.

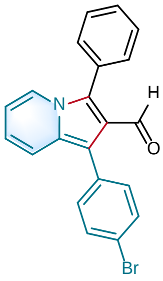

23

According to the general procedure A, a mixture of *α,β*-unsaturated aldehyde (0.2 mmol), heteroaryl ketone (2.5 equiv.), catalyst **3j** (0.04 mmol), AcOH (2.0 equiv.) and $LiSO_3CF_3$ (3.0 equiv.) in CF_3CH_2OH (0.9 mL) were stirred at 80 ºC under Ar atmosphere for 18 h. Work-up gave product 1-(4-bromophenyl)-3-phenylindolizine-2-carbaldehyde (**23**, 67.5 mg, 0.18 mmol, isolated yield 88%) as a yellow solid. **mp**: 150−151 ºC. **FT-IR**: *v* (cm^{-1}): 3046, 2926, 2846, 2749, 1675, 1597, 1521, 1486, 1447, 1426, 1397, 1379, 1356, 1321, 1259, 1230, 1177, 1123, 1102, 1069, 1039, 1022, 1006, 921, 901, 816, 797, 748, 732, 701, 688, 647, 560, 501, 488, 435, 412. **^{1}H NMR** (400 MHz, $CDCl_3$) δ 9.90 (s, 1H), 7.81 (d, *J* = 7.2 Hz, 1H), 7.50 – 7.45 (m, 7H), 7.38 – 7.32 (m, 3H), 6.69 – 6.65 (m, 1H), 6.47 (t, *J* = 6.6 Hz, 1H). **^{13}C NMR** (100 MHz, $CDCl_3$) δ 188.3, 132.1, 131.3, 131.0, 131.0, 130.6, 129.3, 129.1, 128.9, 128.6, 128.2, 122.7, 121.9, 120.8, 120.0, 119.33, 113.5. **ESI-HRMS**: *m/z* calcd. for $C_{21}H_{14}BrNO$ $[M+H]^+$: 376.0337, found 376.0335.

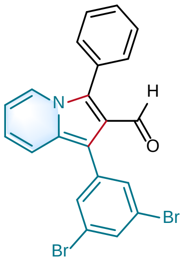

24

According to the general procedure A, a mixture of *α,β*-unsaturated aldehyde (0.2 mmol), heteroaryl ketone (2.5 equiv.), catalyst **3j** (0.04 mmol), AcOH (2.0 equiv.) and $LiSO_3CF_3$ (3.0 equiv.) in CF_3CH_2OH (0.9 mL) were stirred at 80 ºC under Ar atmosphere for 18 h. Work-up gave product 1-(3,5-dibromophenyl)-3-phenylindolizine-2-carbaldehyde (**24**, 86.1 mg, 0.19 mmol, isolated yield 95%) as a yellow solid. **mp**: 57−58 ºC. **FT-IR**: *v* (cm^{-1}): 3064, 2844, 2747, 1673, 1578, 1543, 1523, 1475, 1453, 1405, 1377, 1356, 1321, 1300, 1280, 1259, 1232, 1156, 1123, 1105, 1074, 1047, 1026, 989, 956, 925, 907, 851, 750, 740, 694, 672, 641, 618, 577, 523, 488, 422. **^{1}H NMR** (400 MHz, $CDCl_3$) δ 9.89 (s, 1H), 7.82 (d, *J* = 7.2 Hz, 1H), 7.55 (d, *J* = 4.4 Hz, 3H), 7.50 – 7.46 (m, 5H), 7.38 (d, *J* = 9.2 Hz, 1H), 6.76 – 6.72 (m, 1H), 6.51 (t, *J* = 6.8 Hz, 1H). **^{13}C NMR** (100 MHz, $CDCl_3$) δ 187.9, 148.7, 137.8, 137.3, 137.1, 132.1, 131.1, 129.5, 129.1, 128.2, 124.8, 122.9, 122.4, 120.8, 118.9, 113.6, 112.0. **ESI-HRMS**: *m/z* calcd. for $C_{21}H_{13}Br_2NO$ $[M+H]^+$: 353.9441, found 353.9442.

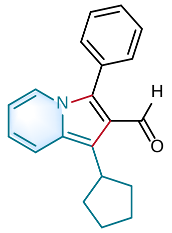

25

According to the general procedure A, a mixture of *α*,*β*-unsaturated aldehyde (0.2 mmol), heteroaryl ketone (2.5 equiv.), catalyst **3j** (0.04 mmol), AcOH (2.0 equiv.) and $LiSO_3CF_3$ (3.0 equiv.) in CF_3CH_2OH (0.9 mL) were stirred at 80 ºC under Ar atmosphere for 18 h. Work-up gave product 1-cyclopentyl-3-phenylindolizine-2-carbaldehyde (**25**, 26.6 mg, 0.09 mmol, isolated yield 46%) as a yellow liquid. **FT-IR**: *v* (cm^{-1}): 2949, 2864, 1669, 1599, 1515, 1447, 1428, 1395, 1358, 1315, 1241, 1224, 1164, 1074, 1026, 1001, 973, 927, 886, 744, 725, 696, 554, 495, 439, 406. **^{1}H NMR** (400 MHz, $CDCl_3$) δ 9.88 (s, 1H), 7.72 (d, *J* = 7.2 Hz, 1H), 7.48 – 7.39 (m, 6H), 6.57 – 6.53 (m, 1H), 6.38 – 6.34 (m, 1H), 3.84 – 3.76 (m, 1H), 1.96 – 1.89 (m, 6H), 1.69 – 1.67 (m, 2H). **^{13}C NMR** (100 MHz, $CDCl_3$) δ 189.4, 132.5, 131.2, 129.8, 129.0, 129.0, 129.0, 122.9, 122.6, 120.0, 118.3, 117.5, 112.6, 36.2, 33.3, 26.4. **ESI-HRMS**: *m/z* calcd. for $C_{20}H_{19}NO$ $[M+H]^+$: 290.1547, found 290.1545.

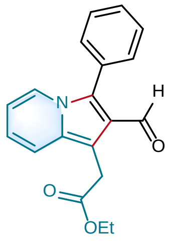

26

According to the general procedure D, a mixture of *α*,*β*-unsaturated aldehyde (0.2 mmol), heteroaryl ketone (2.5 equiv.), catalyst **3j** (0.04 mmol), AcOH (2.0 equiv.) and $LiSO_3CF_3$ (3.0 equiv.) in CF_3CH_2OH (0.9 mL) were stirred at 80 ºC under Ar atmosphere for 36 h. Work-up gave product ethyl 2-(2-formyl-3-phenylindolizin-1-yl)acetate (**26**, 19.6 mg, 0.06 mmol, isolated yield 32%) as a yellow liquid. **FT-IR**: *v* (cm^{-1}): 3056, 2978, 2926, 2835, 2753, 1731, 1665, 1525, 1445, 1389, 1356, 1321, 1249, 1226, 1212, 1175, 1154, 1105, 1076, 1028, 933, 868, 833, 752, 736, 701, 672, 530, 482, 435. **^{1}H NMR** (400 MHz, $CDCl_3$) δ 9.88 (s, 1H), 7.81 (d, *J* = 7.6 Hz, 1H), 7.50 – 7.43 (m, 5H), 7.33 (d, *J* = 9.2 Hz, 1H), 6.67 (ddd, *J* = 9.2, 6.4, 0.8 Hz, 1H), 6.45 – 6.41 (m, 1H), 4.13 (q, *J* = 7.2 Hz, 2H), 4.06 (s, 2H), 1.23 (t, *J* = 7.2 Hz,

1H). **^{13}C NMR** (100 MHz, $CDCl_3$) δ 189.3, 171.8, 132.0, 131.6, 131.1, 129.2, 129.1, 128.5, 123.1, 122.7, 119.0, 118.7, 112.9, 105.7, 60.8, 30.1, 14.3. **ESI-HRMS**: *m/z* calcd. for $C_{19}H_{17}NO_3$ $[M+H]^+$: 308.1287, found 308.1281.

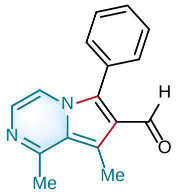

27

According to the general procedure D, a mixture of *α,β*-unsaturated aldehyde (0.2 mmol), heteroaryl ketone (2.5 equiv.), catalyst **3j** (0.04 mmol), AcOH (2.0 equiv.) and $LiSO_3CF_3$ (3.0 equiv.) in CF_3CH_2OH (0.9 mL) were stirred at 80 ºC under Ar atmosphere for 36 h. Work-up gave product 1,8-dimethyl-6-phenylpyrrolo[1,2-a]pyrazine-7-carbaldehyde (**27**, 21.5 mg, 0.09 mmol, isolated yield 43%) as a yellow liquid. **FT-IR**: *ν* (cm^{-1}): 3060, 2924, 2852, 2737, 1675, 1607, 1502, 1465, 1453, 1432, 1387, 1372, 1352, 1284, 1208, 1152, 1069, 1024, 956, 826, 760, 705, 593, 556, 488. **^{1}H NMR** (400 MHz, $CDCl_3$) δ 9.94 (s, 1H), 7.51 – 7.45 (m, 4H), 7.39 – 7.37 (m, 2H), 7.21 (d, *J* = 5.2 Hz, 1H), 2.84 (s, 3H), 2.77 (s, 3H). **^{13}C NMR** (100 MHz, $CDCl_3$) δ 189.2, 157.1, 132.7, 130.8, 129.6, 129.2, 127.8, 127.6, 125.7, 123.5, 116.5, 113.7, 24.8, 11.9. **ESI-HRMS**: *m/z* calcd. for $C_{16}H_{14}N_2O$ $[M+H]^+$: 251.1184, found 251.1178.

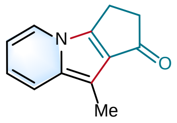

35

According to the general procedure E, a mixture of *α,β*-unsaturated ketone (0.2 mmol), heteroaryl ketone (2.5 equiv.), catalyst chitosan (0.04 mmol), formic acid (4.0 equiv.) in H_2O (1.0 mL) were stirred at 120 ºC under Ar atmosphere for 18 h. Work-up gave product 9-methyl-2,3-dihydro-1H-cyclopenta[b]indolizin-1-one (**35,** 8.5 mg, 0.05 mmol, isolated yield 23%) as a yellow solid. **mp**: 134−135 ºC. **FT-IR**: *ν* (cm^{-1}): 2910, 2856, 1679, 1624, 1517, 1477, 1434, 1403, 1381, 1313, 1284, 1253, 1228, 1195, 1148, 1137, 1065, 1032, 981, 956, 876, 826, 795, 734, 709, 682, 655, 624, 548, 497, 418. **^{1}H NMR** (400 MHz, $CDCl_3$) δ 7.51 (d, *J* = 4.0 Hz, 1H), 7.23 (d, *J* = 8.0 Hz, 1H), 6.53 (d, *J* = 4.0 Hz, 1H), 6.43 – 6.39 (m, 1H), 3.01

– 2.92 (m, 4H), 2.36 (s, 3H). **^{13}C NMR** (100 MHz, $CDCl_3$) δ 199.2, 146.2, 135.6, 128.4, 122.4, 119.7, 116.8, 111.5, 103.3, 41.4, 19.4, 8.7. **ESI-HRMS**: *m/z* calcd. for $C_{12}H_{11}NO$ $[M+H]^+$: 186.0919, found 186.0917.

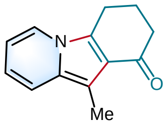

36

According to the general procedure E, a mixture of *α*,*β*-unsaturated ketone (0.2 mmol), heteroaryl ketone (2.5 equiv.), catalyst chitosan (0.04 mmol), formic acid (4.0 equiv.) in H_2O (1.0 mL) were stirred at 120 °C under Ar atmosphere for 18 h. Work-up gave product 10-methyl-3,4-dihydropyrido[1,2-a]indol-1(2H)-one (**36**, 15.2 mg, 0.08 mmol, isolated yield 38%) as a yellow solid.[7] **mp**: 111−112 °C. **FT-IR**: *ν* (cm^{-1}): 2920, 1649, 1523, 1432, 1414, 1368, 1331, 1265, 1228, 1183, 1140, 1082, 997, 925, 894, 859, 812, 783, 736, 713, 628, 598, 577, 560, 530, 466, 420. **^{1}H NMR** (400 MHz, $CDCl_3$) δ 7.48 (d, *J* = 7.2 Hz, 1H), 7.24 (d, *J* = 9.2 Hz, 1H), 6.49 (dd, *J* = 9.0, 6.4 Hz, 1H), 6.44 – 6.41 (m, 1H), 2.85 (t, *J* = 6.2 Hz, 2H), 2.54 – 2.51 (m, 2H), 2.47 (s, 3H), 2.19 (p, *J* = 6.4 Hz, 2H). **^{13}C NMR** (100 MHz, $CDCl_3$) δ 197.2, 130.8, 129.9, 121.8, 121.0, 119.1, 116.0, 112.1, 107.8, 39.4, 23.5, 21.1, 9.6. **ESI-HRMS**: *m/z* calcd. for $C_{13}H_{13}NO$ $[M+H]^+$: 200.1075, found 200.1071.

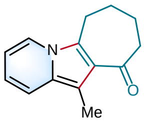

37

According to the general procedure E, a mixture of *α*,*β*-unsaturated ketone (0.2 mmol), heteroaryl ketone (2.5 equiv.), catalyst chitosan (0.04 mmol), formic acid (4.0 equiv.) in H_2O (1.0 mL) were stirred at 120 °C under Ar atmosphere for 18 h. Work-up gave product 11-methyl-6,7,8,9-tetrahydro-10H-cyclohepta[b]indolizin-10-one (**37**, 22.2 mg, 0.10 mmol, isolated yield 52%) as a yellow liquid. **FT-IR**: *ν* (cm^{-1}): 2930, 2860, 1642, 1521, 1492, 1424, 1331, 1257, 1230, 1195, 1150, 1090, 1051, 991, 954, 909, 791, 727, 645, 558, 536, 480, 422. **^{1}H NMR** (400 MHz, $CDCl_3$) δ 7.57 (d, *J* = 7.2 Hz, 1H), 7.28 (d, *J* = 9.2 Hz, 1H), 6.52 – 6.43 (m, 2H), 2.98 – 2.95 (m, 2H), 2.76 – 2.73 (m, 2H), 2.42 (s, 3H), 1.98 – 1.94 (m, 2H). 1.90 – 1.86 (m, 2H). **^{13}C NMR** (100 MHz, $CDCl_3$)δ 129.4, 127.3, 124.9, 121.6, 118.9, 115.4,

112.1, 110.0, 44.3, 25.3, 25.0, 22.5, 10.1. **ESI-HRMS**: *m/z* calcd. for $C_{14}H_{15}NO$ $[M+H]^+$: 214.1232, found 214.1225.

Supplementary References

1 Zeng, K., Mei, R., Zhang, X. C., Andreas, L. B. & Zhang, K. Direct nitrogen interception from chitin/chitosan for imidazo [1, 5-a] pyridines. *Chem. Commun.* **58**, 6068-6071 (2022)

2 Reux, B., Nevalainen, T., Raitio, K. H. & Koskinen, A. M. Synthesis of quinolinyl and isoquinolinyl phenyl ketones as novel agonists for the cannabinoid CB2 receptor. *Biorg. Med. Chem.* **17**, 4441-4447 (2009).

3 Zeng, K. *et al.* Anomeric stereoauxiliary cleavage of the C-N bond of D-glucosamine for the preparation of imidazo [1, 5-α] pyridines. *Chem. Eur. J.* **28**, e202200648 (2022)

4 Sheldrick, G. M. SHELXT–Integrated space-group and crystal-structure determination. *Acta Crystallogr. A* **71**, 3-8 (2015).

5 Sheldrick, G. M. Crystal structure refinement with SHELXL. *Acta Crystallogr. C Struct. Chem.* **71**, 3-8 (2015).

6 SADABS; BRUKER AXS GmbH, Karlsruhe, Germany, 2016.

7 Yuan, Y.-C., Liu, T.-Z. & Zhao, B.-X. Metal-Free Catalyzed Synthesis of Fluorescent Indolizine Derivatives. *J. Org. Chem.* **86**, 12737-12744 (2021).
