# Supplementary material for: A recyclable stereoauxiliary aminocatalyzed strategy for one-pot synthesis of indolizine-2-carbaldehydes
Source: Commun Chem. 2023 Feb 23;6:40. doi: 10.1038/s42004-023-00828-2 (PMC9950359; doi:10.1038/s42004-023-00828-2)
Supplement: Supplementary file 2 — Description of Additional Supplementary Files [file 42004_2023_828_MOESM2_ESM.pdf]

Description of Additional Supplementary Files

File name: Supplementary Data 1

Description: The NMR spectra of all compounds.

File name: Supplementary Data 2

Description: The X-ray crystallographic coordinates for structures 19.
