# Supplementary material for: A recyclable stereoauxiliary aminocatalyzed strategy for one-pot synthesis of indolizine-2-carbaldehydes
Source: Commun Chem. 2023 Feb 23;6:40. doi: 10.1038/s42004-023-00828-2 (PMC9950359; doi:10.1038/s42004-023-00828-2)

^{1}H NMR spectra of 3p (400 MHz, DMSO-d_6)

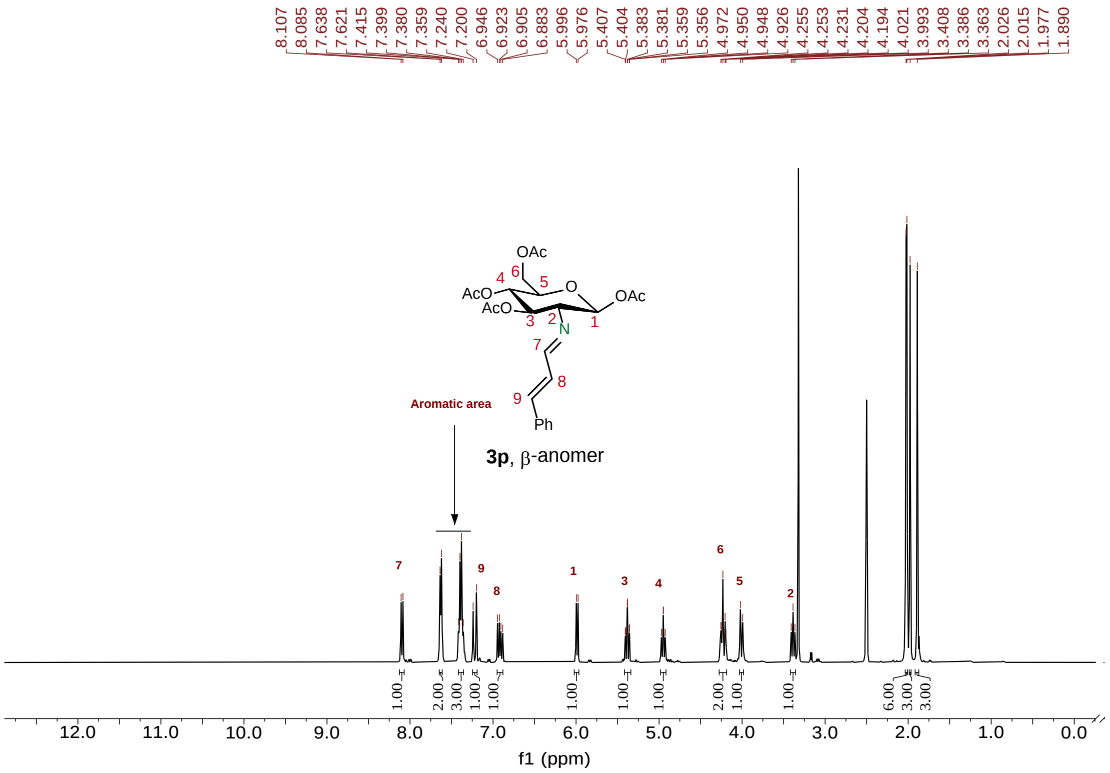

^{13}C NMR spectra of 3p (100 MHz, DMSO-d_6)

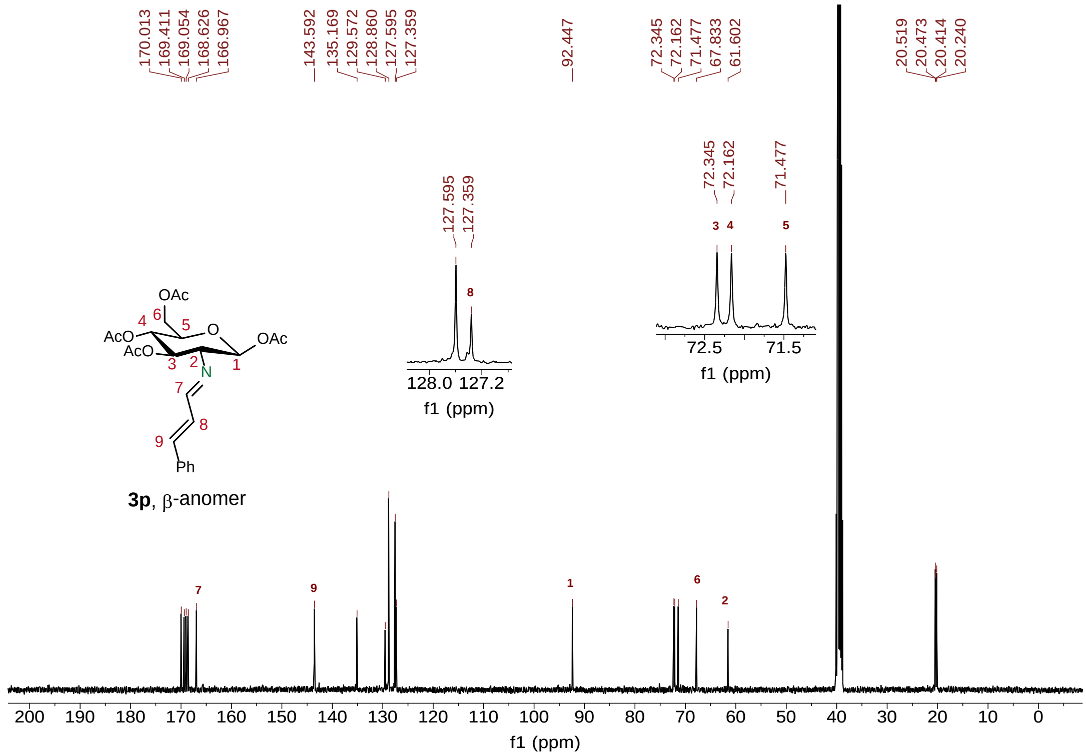

^{1}H NMR spectra of 3q (400 MHz, DMSO-d_6)

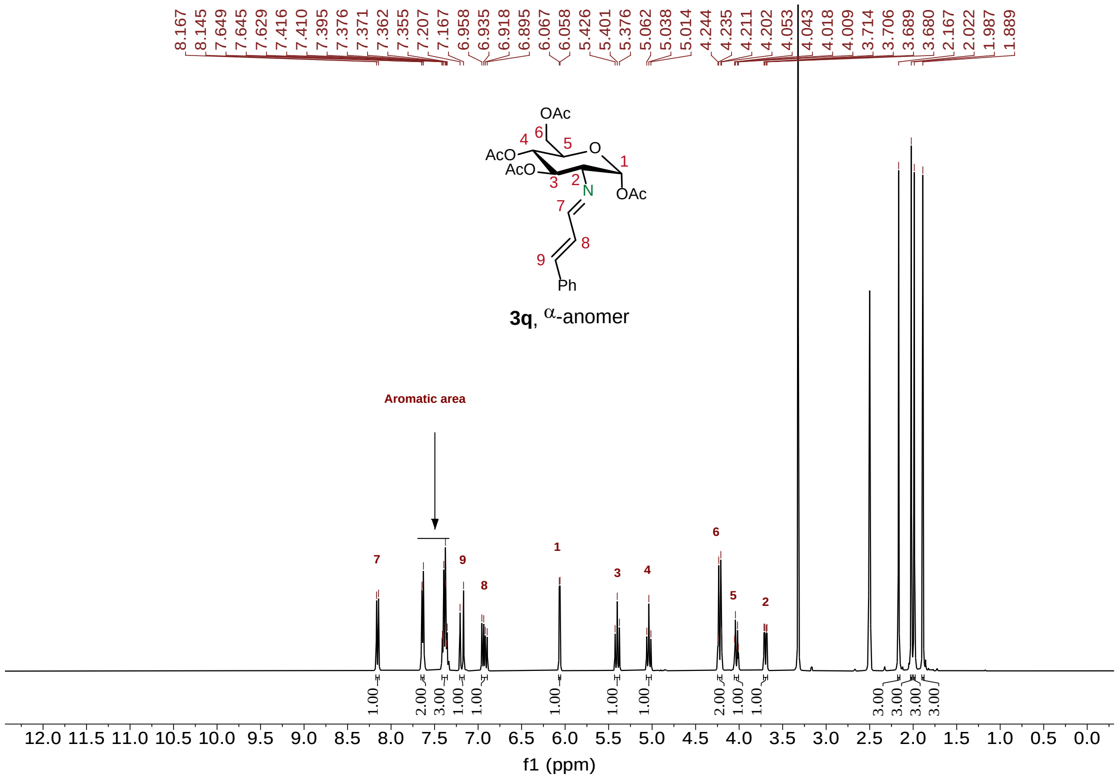

^{13}C NMR spectra of 3q (100 MHz, DMSO-d_6)

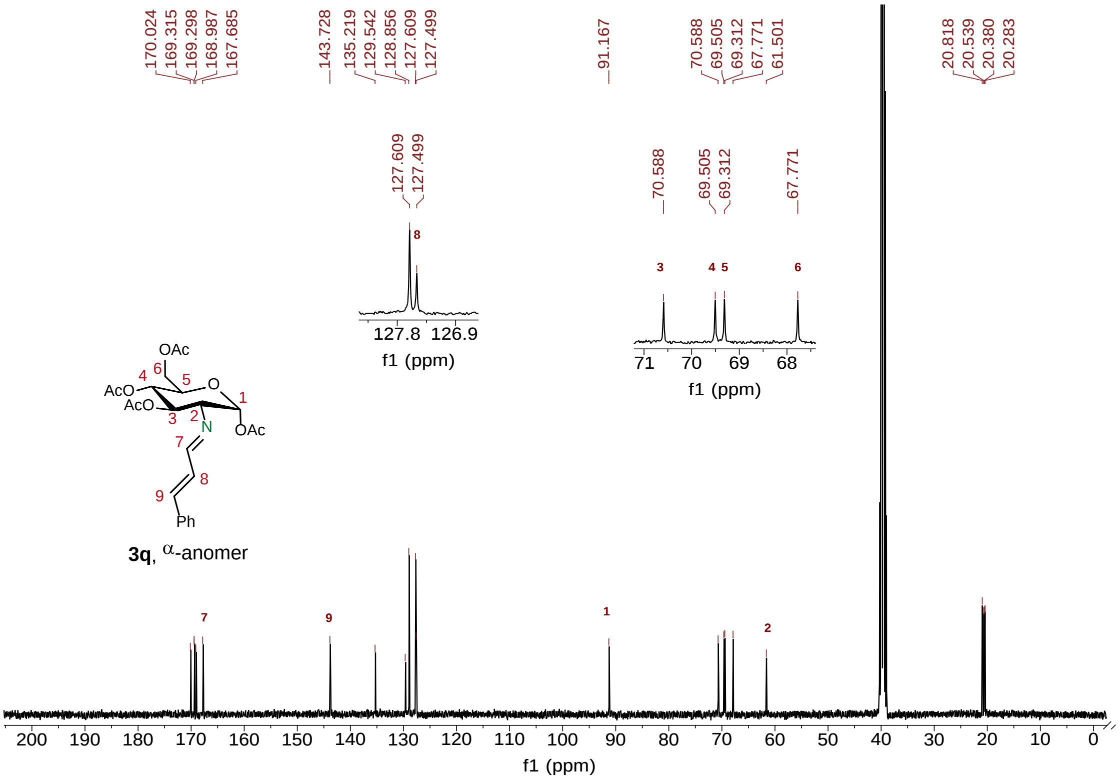

^{1}H NMR spectra of 4 (400 MHz, $CDCl_3$)

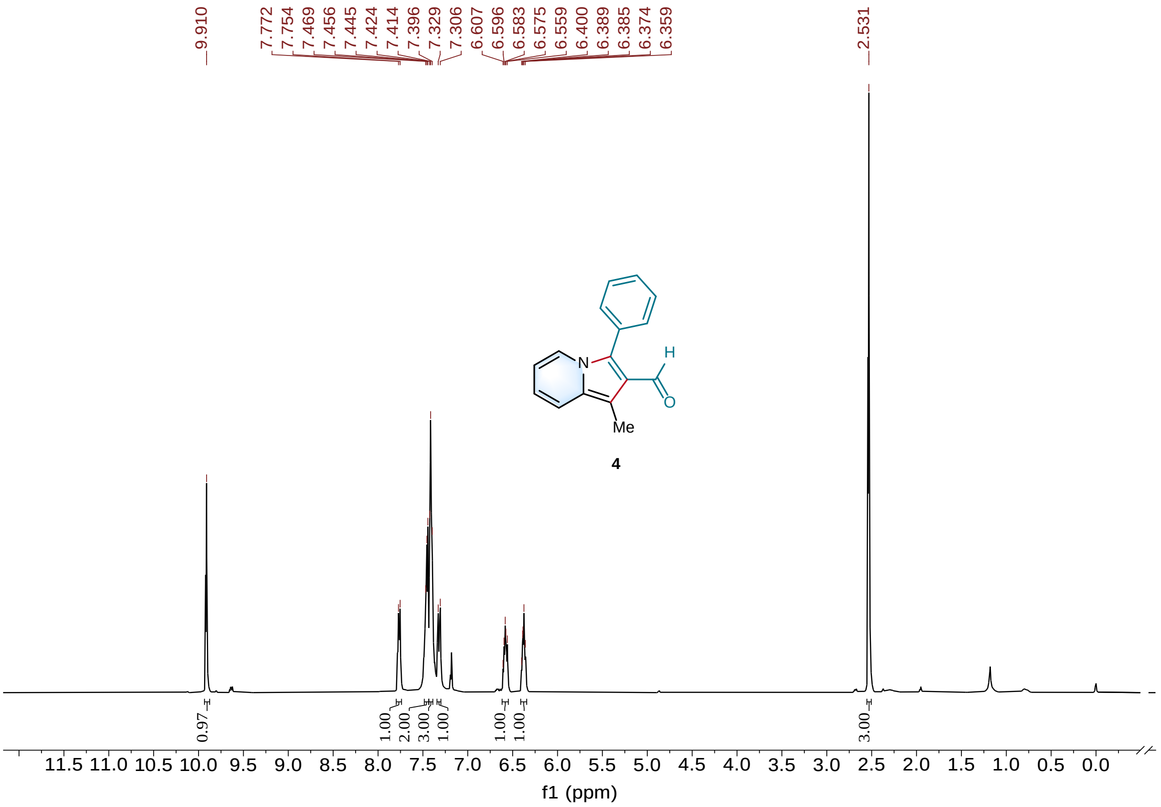

^{13}C NMR spectra of 4 (100 MHz, CDCl$_3$)

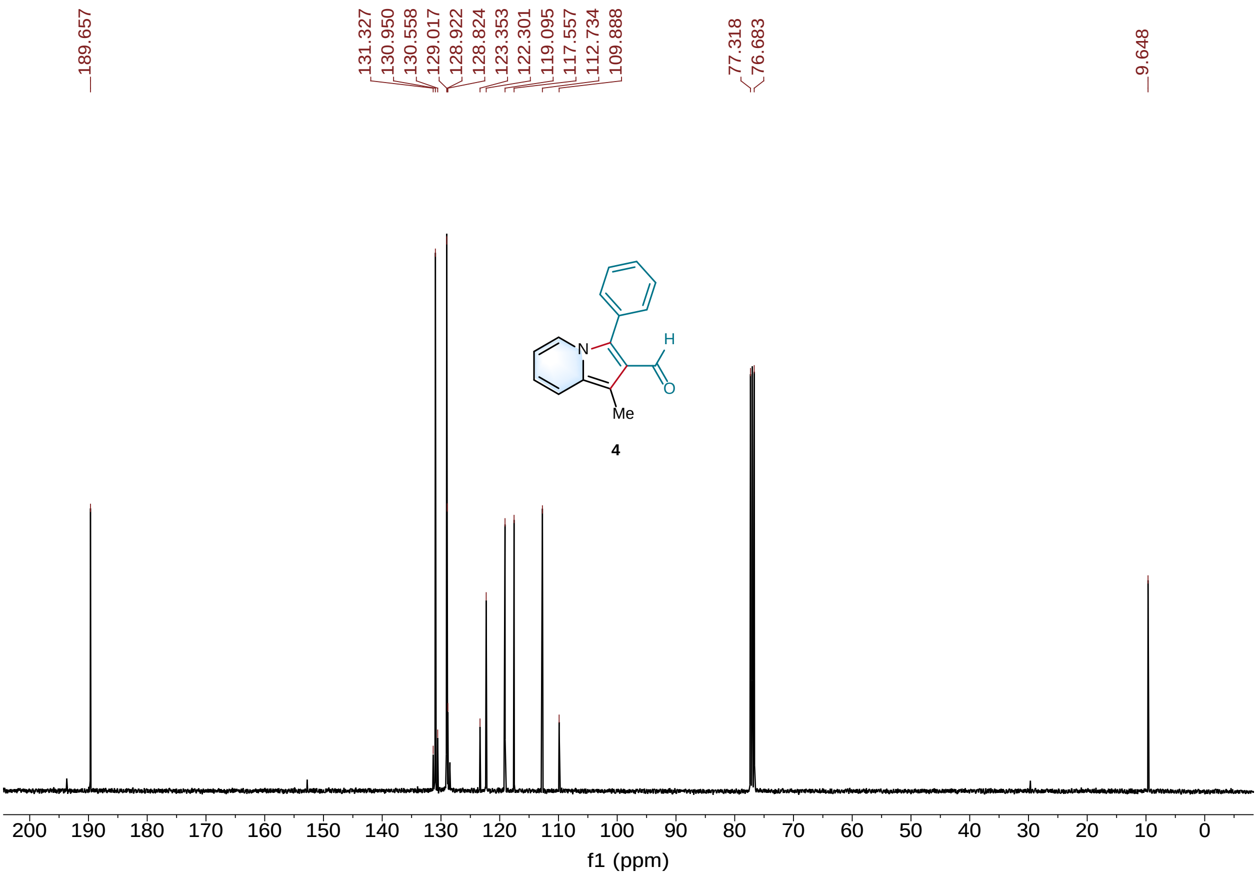

^{1}H NMR spectra of 5 (400 MHz, CDCl$_3$)

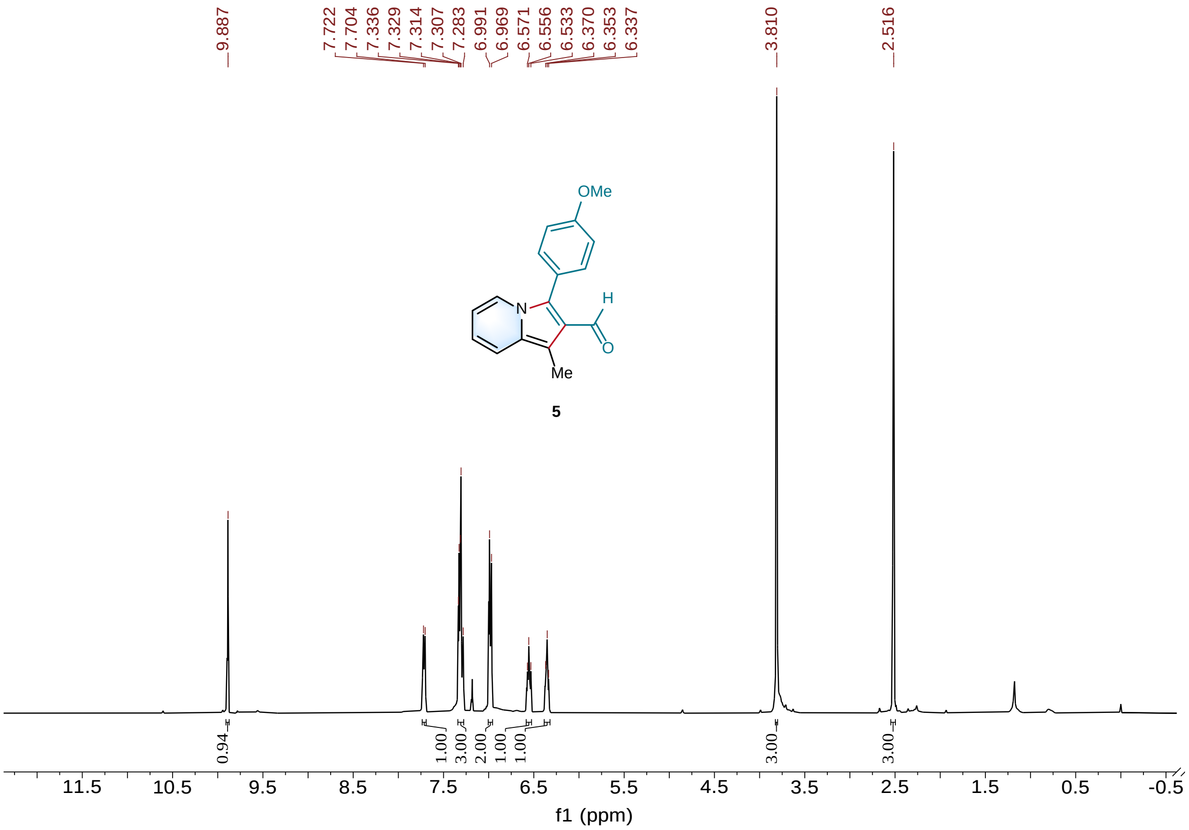

^{13}C NMR spectra of 5 (100 MHz, $CDCl_3$)

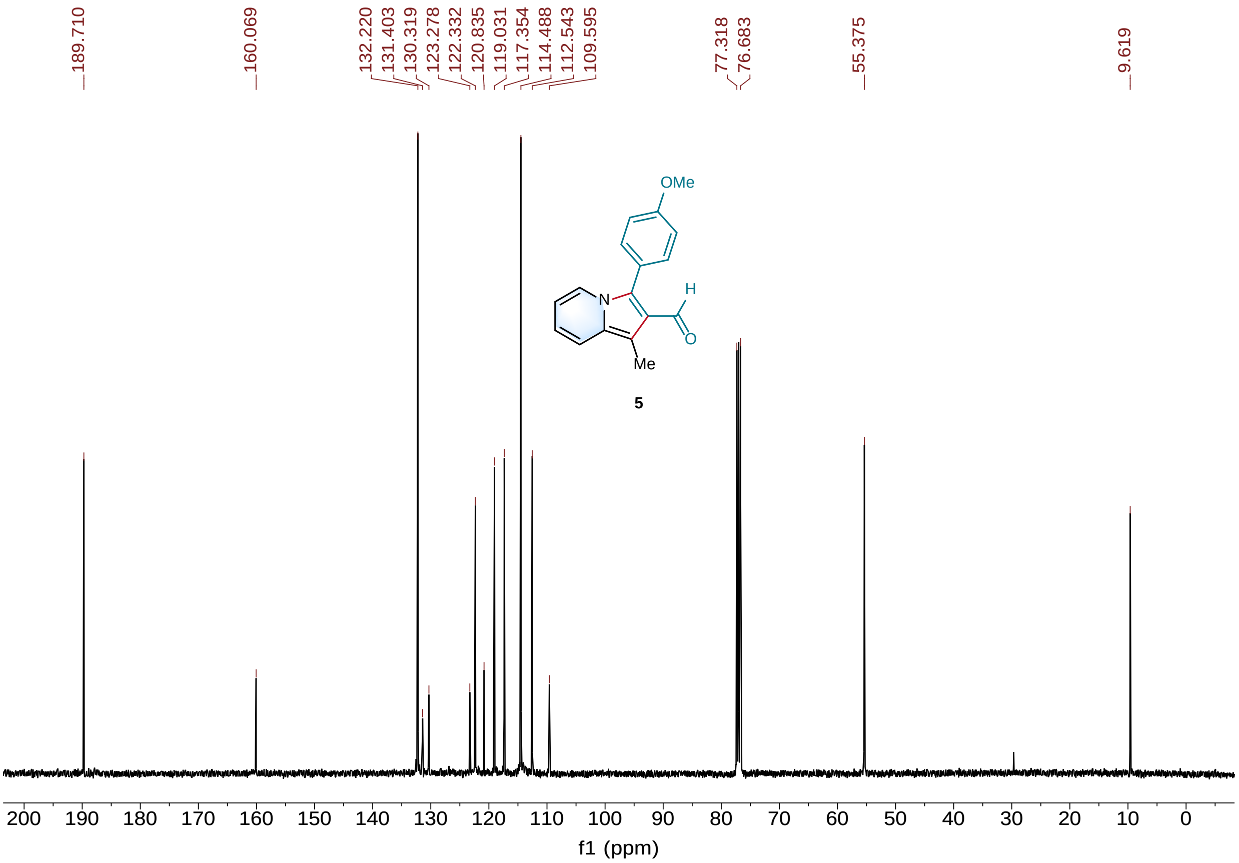

^{1}H NMR spectra of 6 (400 MHz, $CDCl_3$)

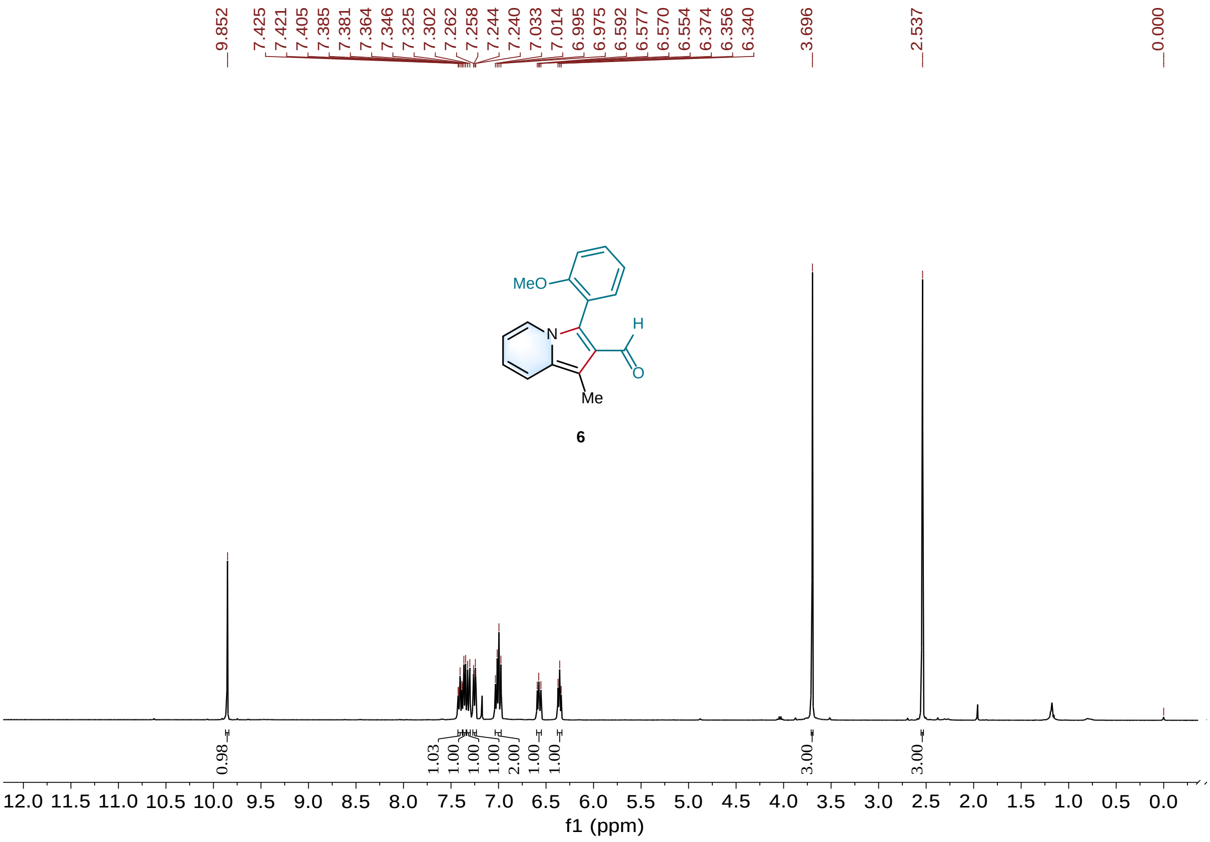

^{13}C NMR spectra of 6 (100 MHz, $CDCl_3$)

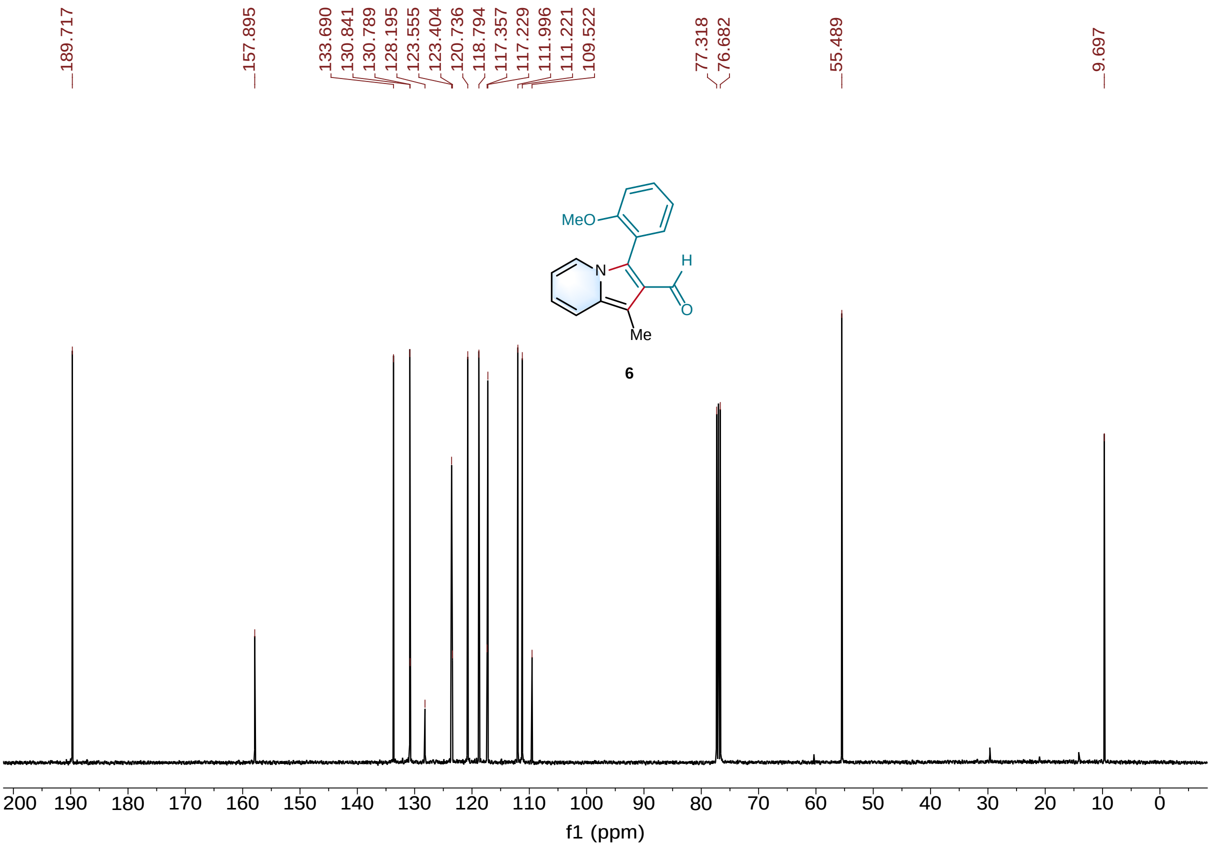

^{1}H NMR spectra of 7 (400 MHz, $CDCl_3$)

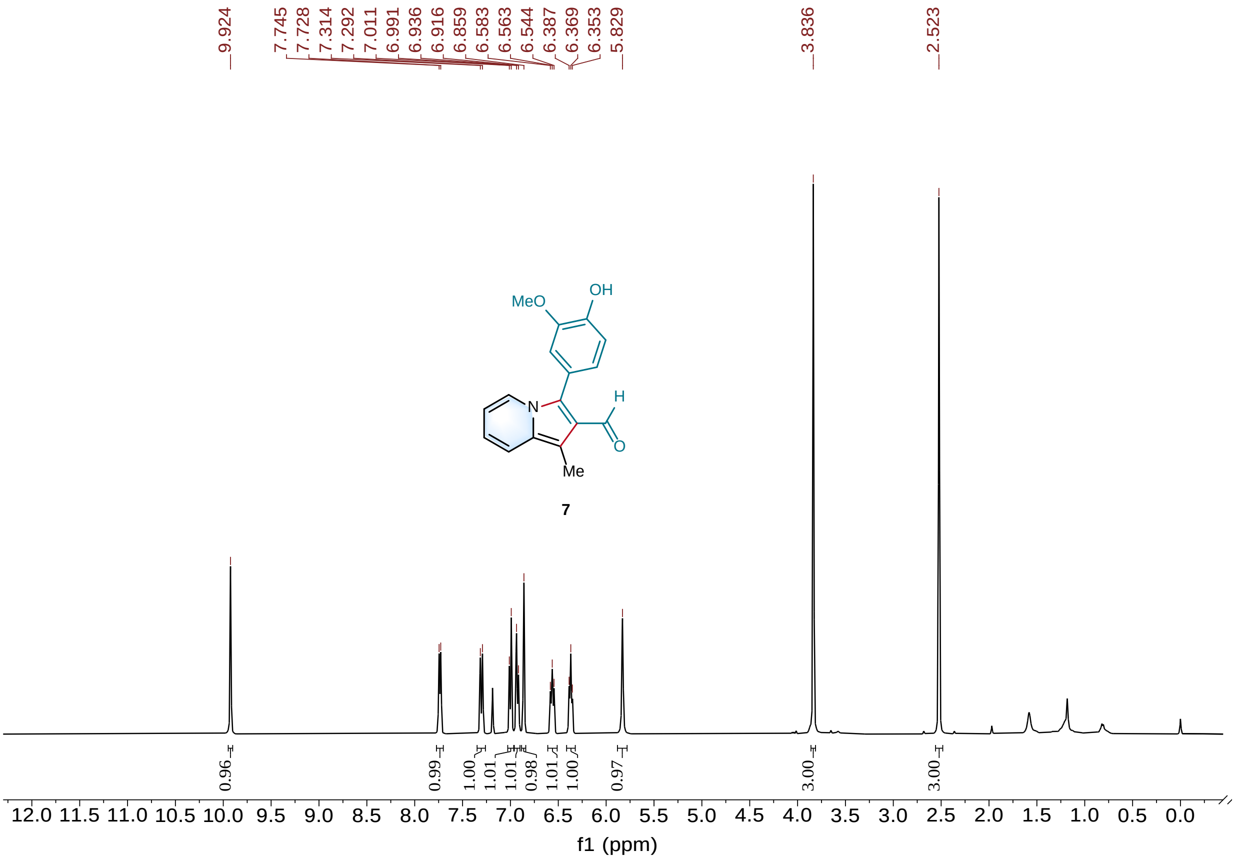

^{13}C NMR spectra of 7 (100 MHz, $CDCl_3$)

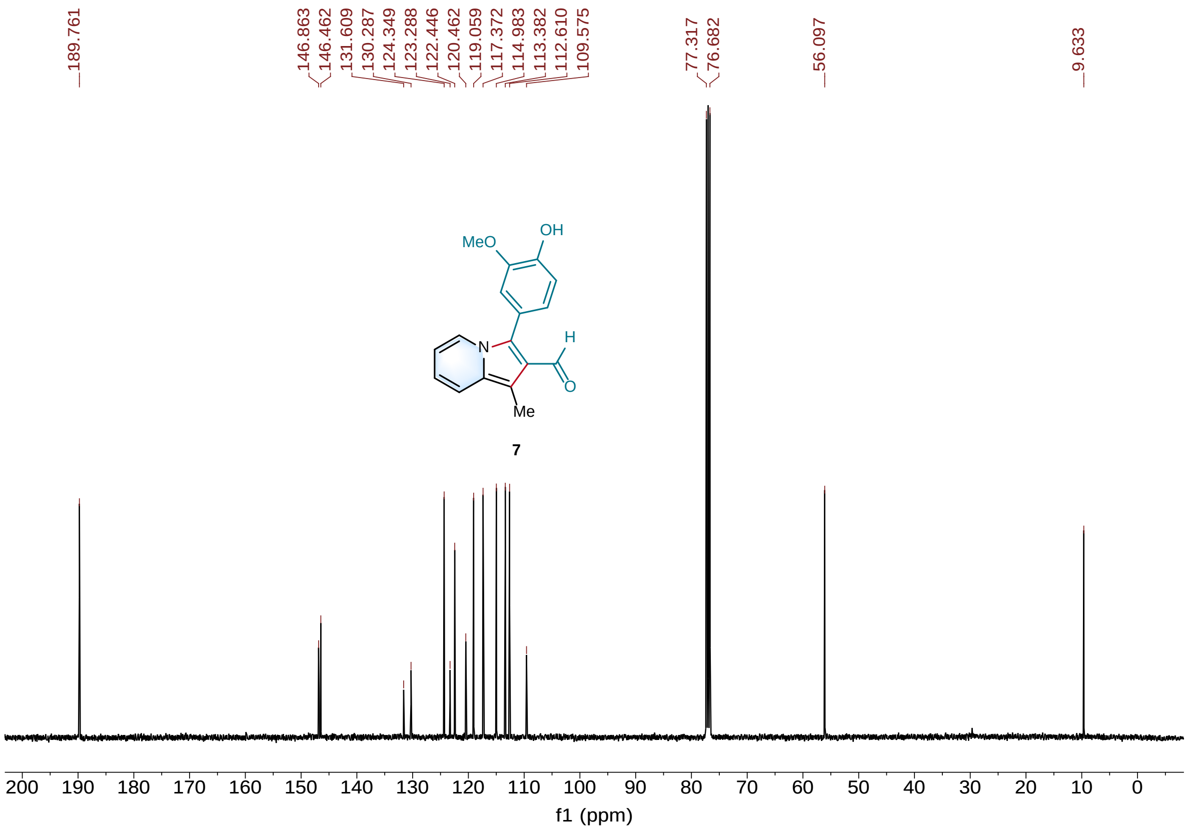

^{1}H NMR spectra of 8 (400 MHz, $CDCl_3$)

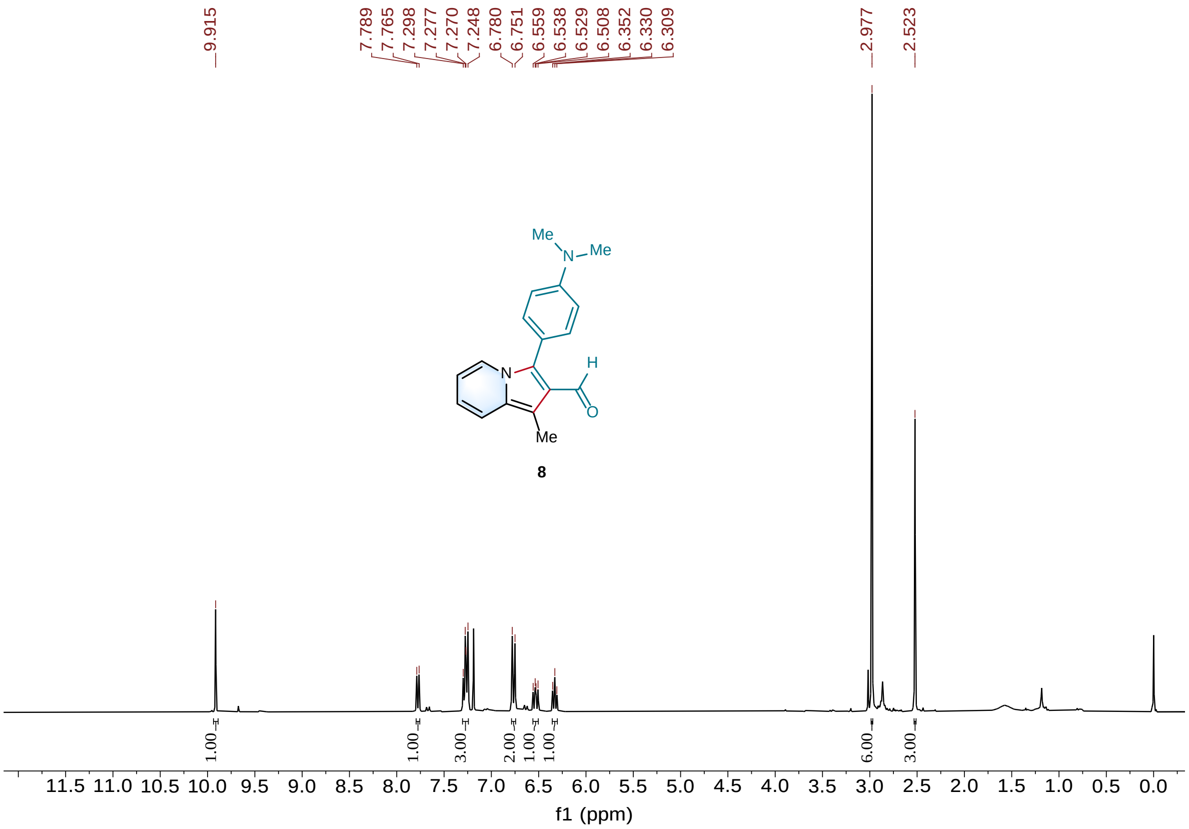

^{13}C NMR spectra of 8 (100 MHz, $CDCl_3$)

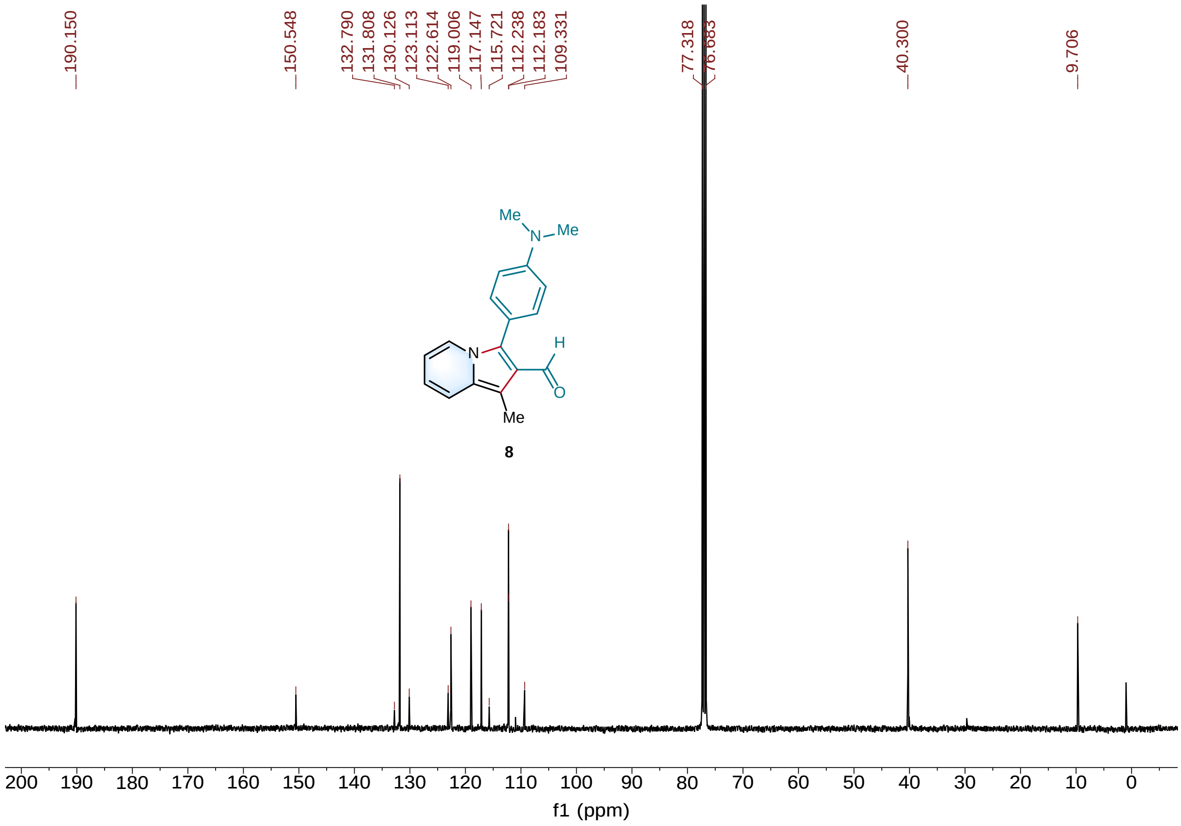

^{1}H NMR spectra of 9 (400 MHz, $CDCl_3$)

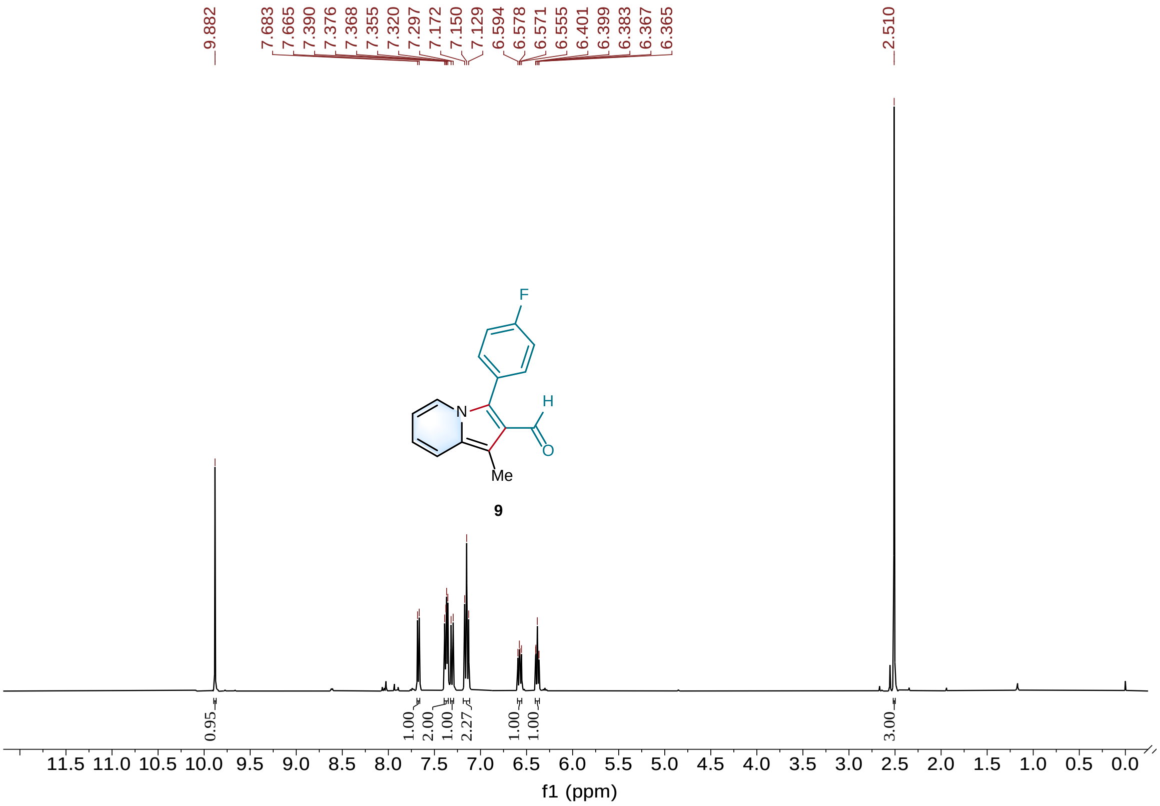

^{13}C NMR spectra of 9 (100 MHz, $CDCl_3$)

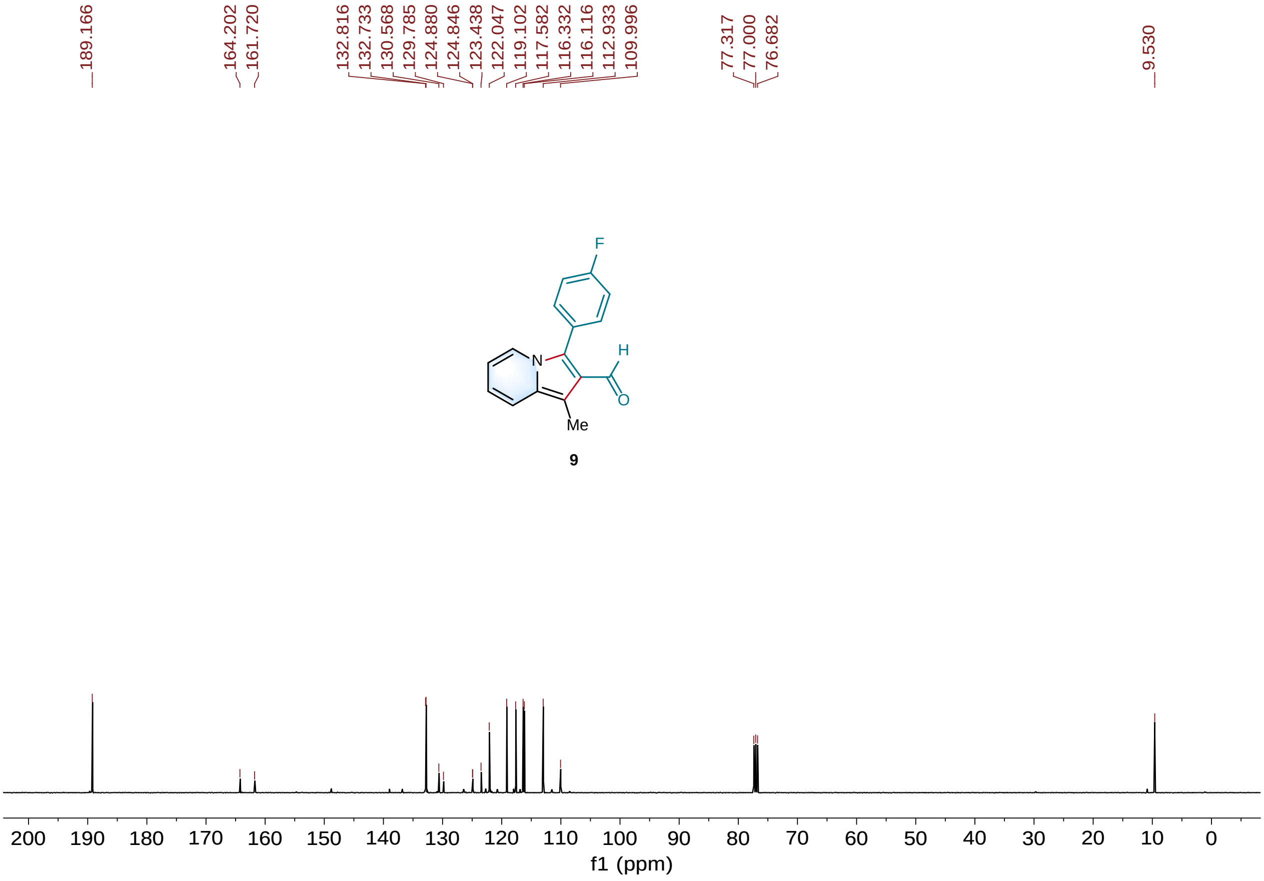

^{19}F NMR spectra of 9 (375 MHz, $CDCl_3$)

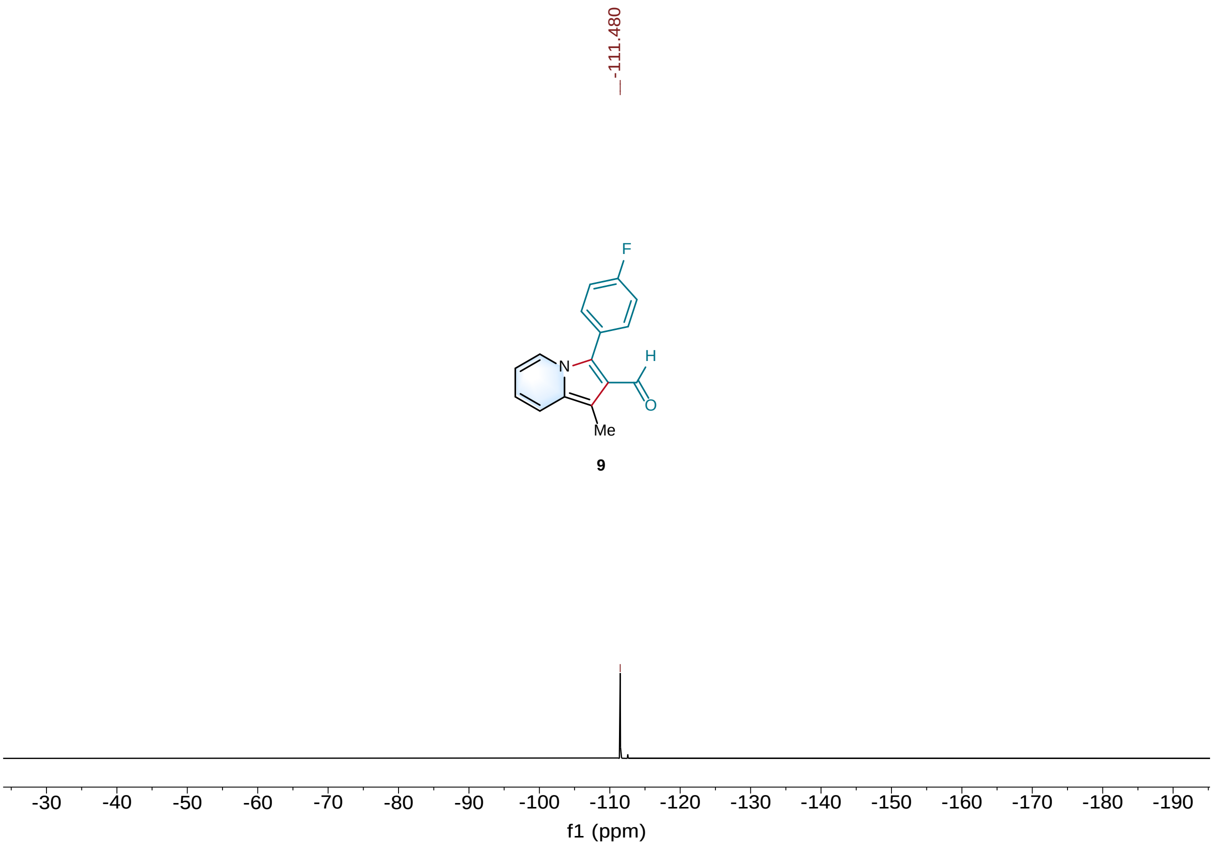

^{1}H NMR spectra of 10 (400 MHz, $CDCl_3$)

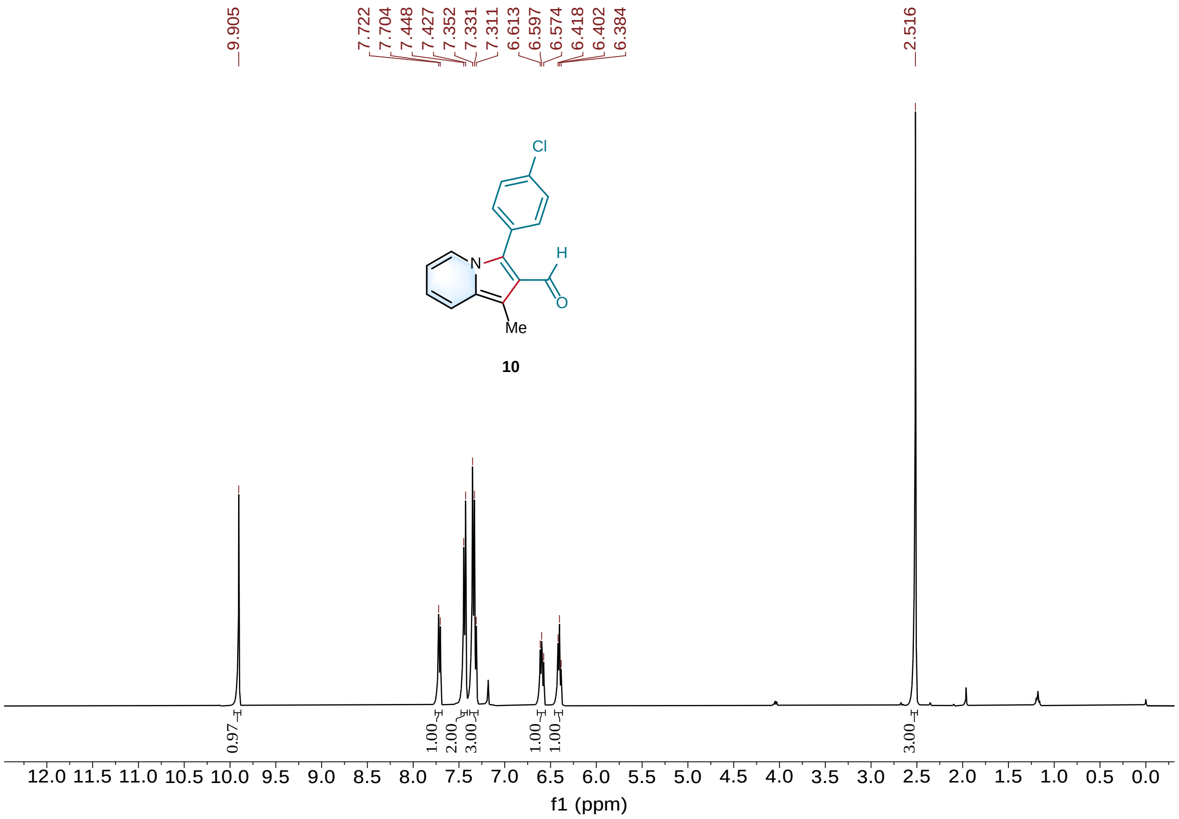

^{13}C NMR spectra of 10 (100 MHz, $CDCl_3$)

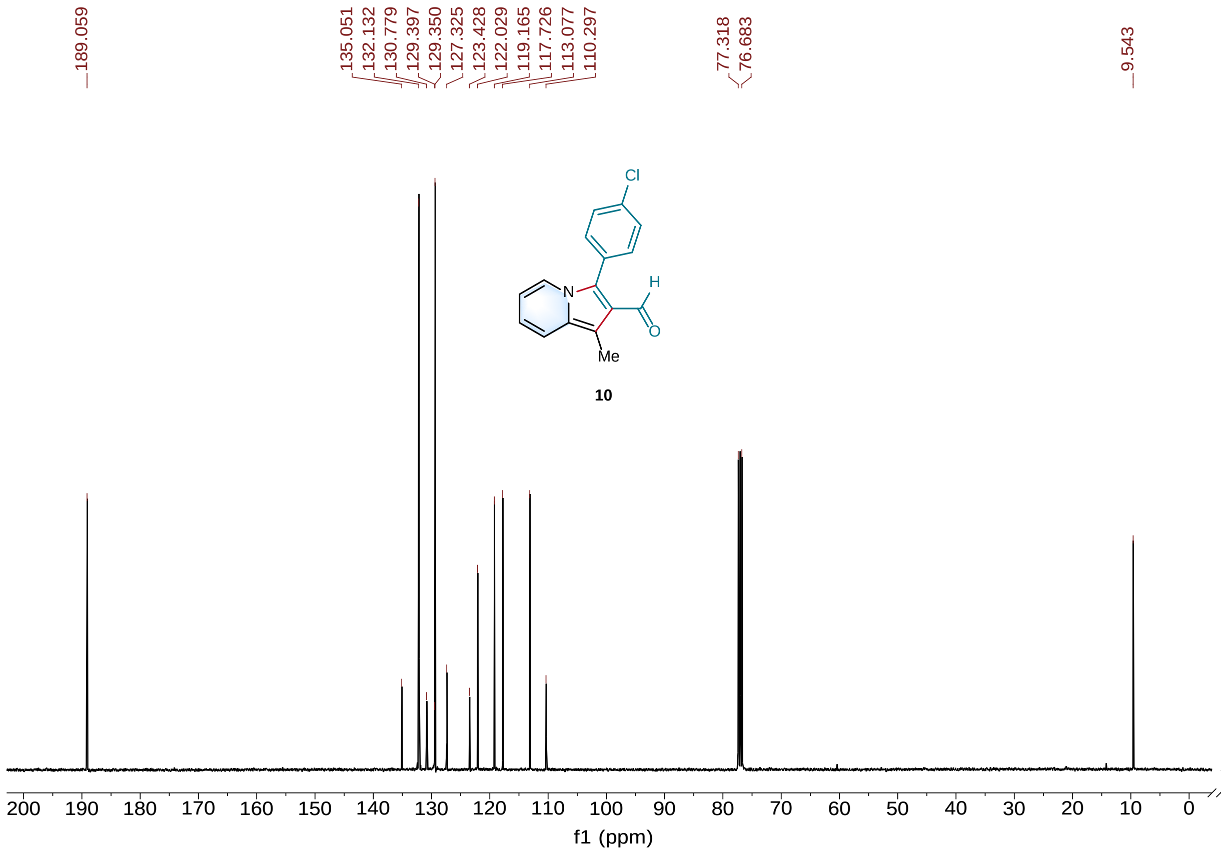

^{1}H NMR spectra of 11 (400 MHz, $CDCl_3$)

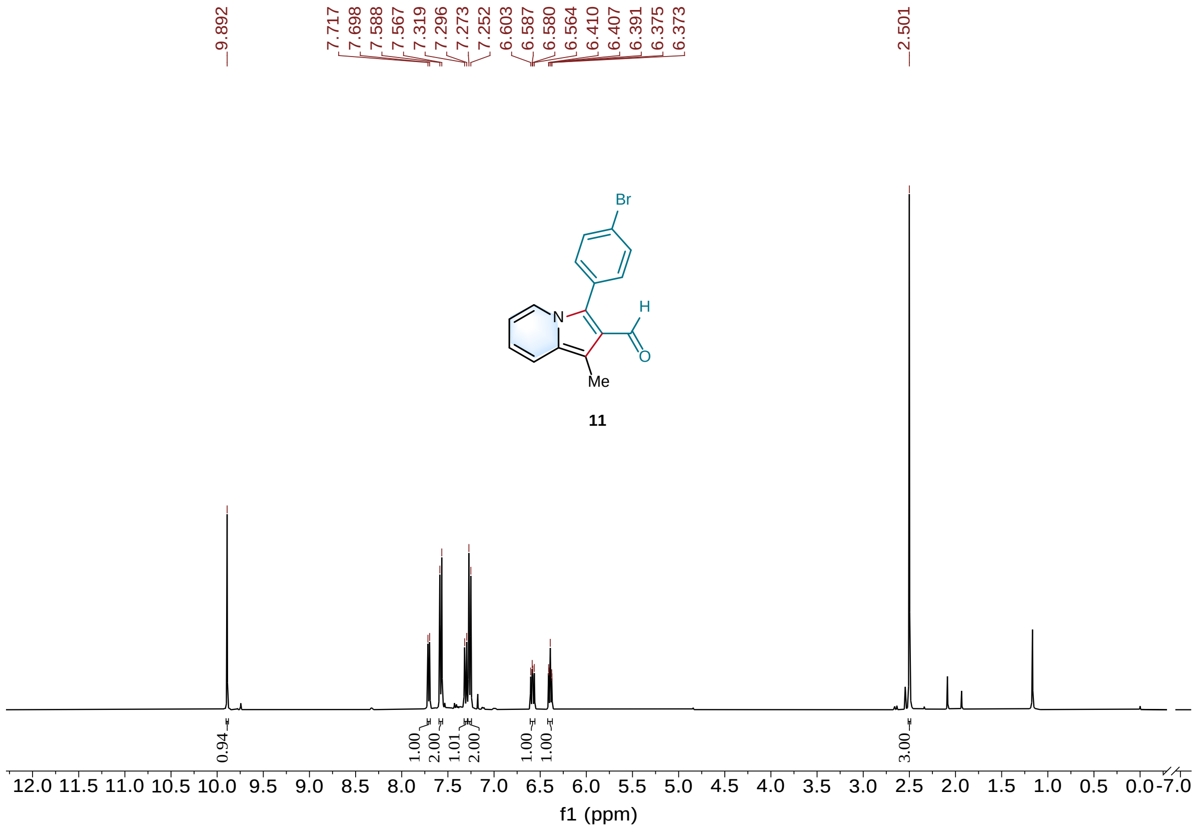

^{13}C NMR spectra of 11 (100 MHz, $CDCl_3$)

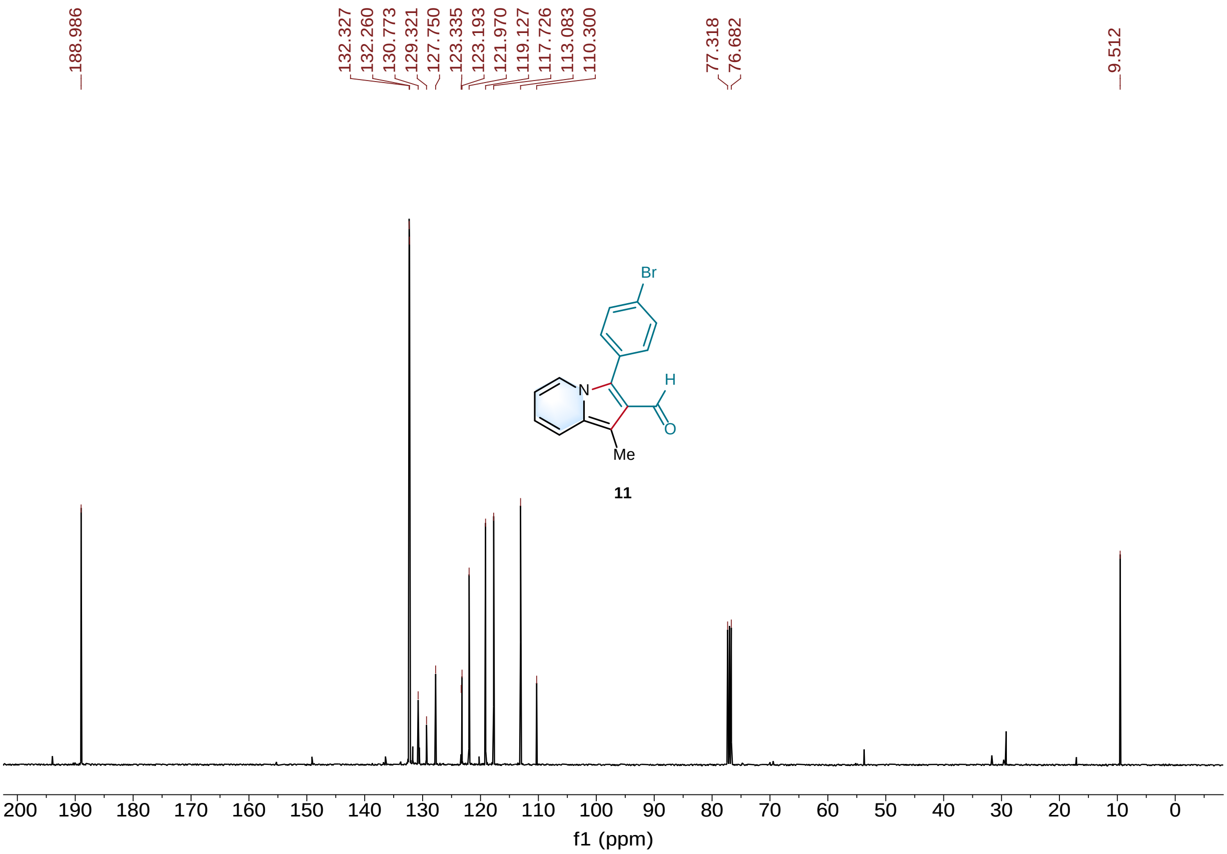

^{1}H NMR spectra of 12 (400 MHz, $CDCl_3$)

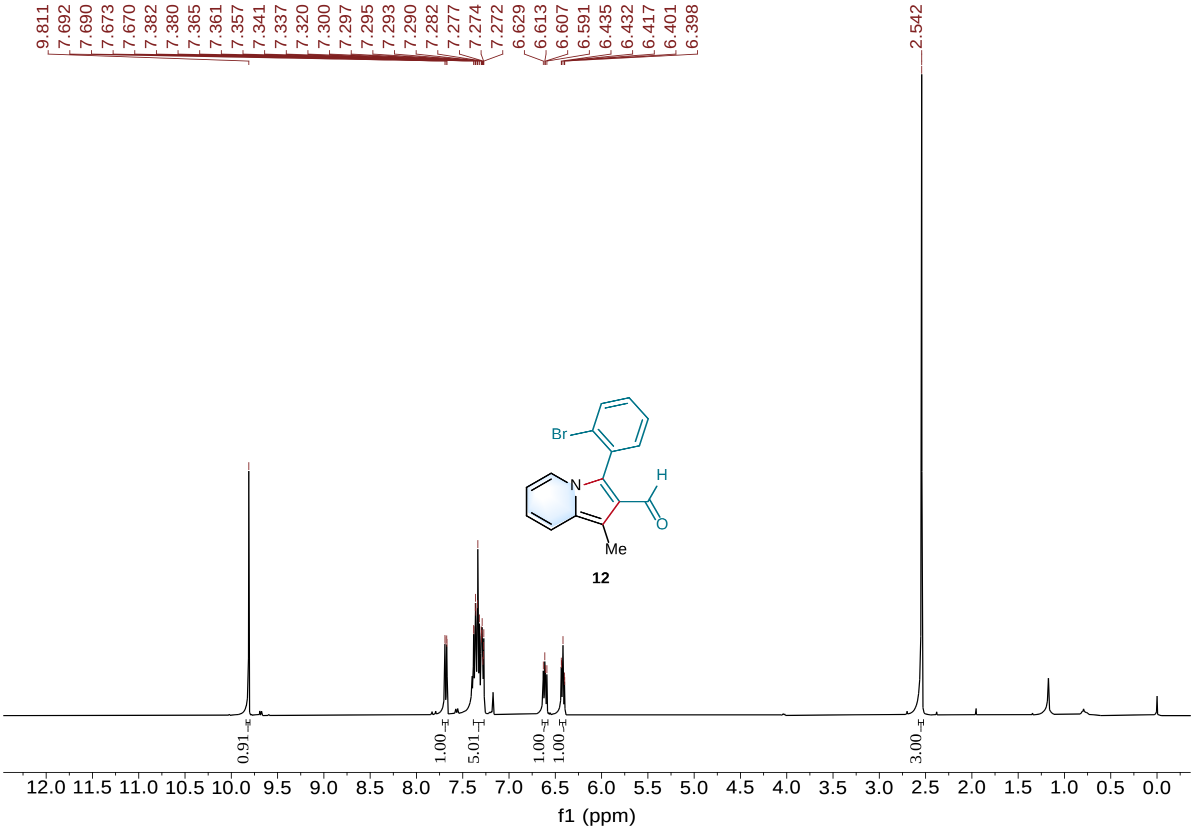

^{13}C NMR spectra of 12 (100 MHz, $CDCl_3$)

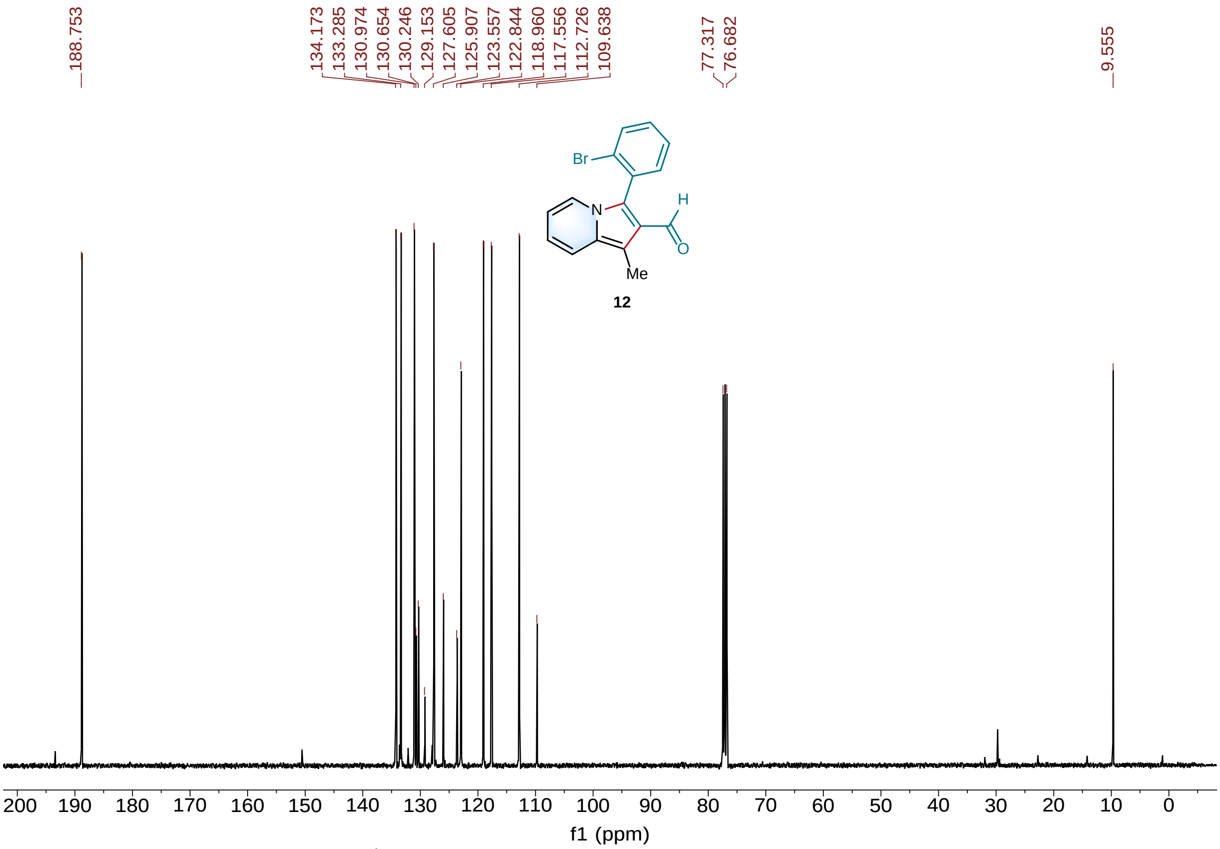

^{1}H NMR spectra of 13 (400 MHz, $CDCl_3$)

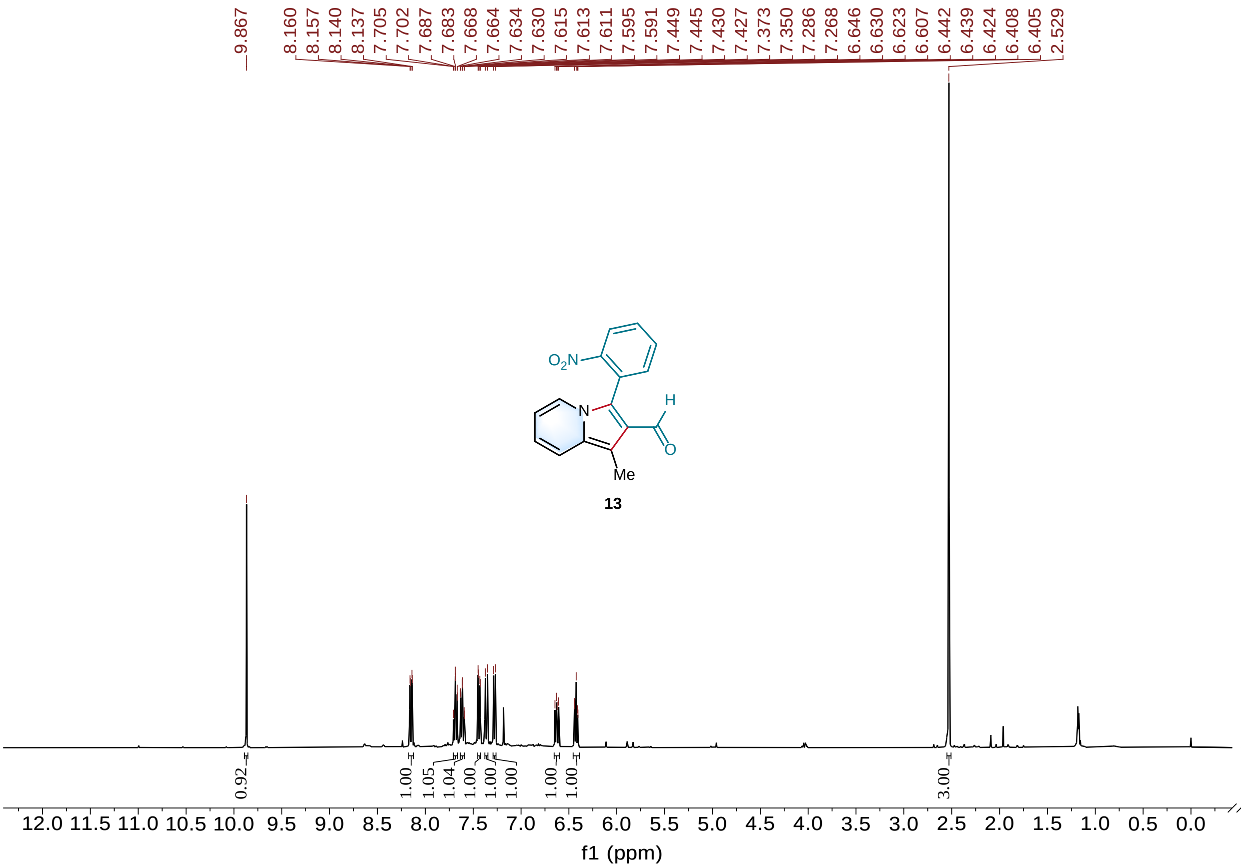

^{13}C NMR spectra of 13 (100 MHz, $CDCl_3$)

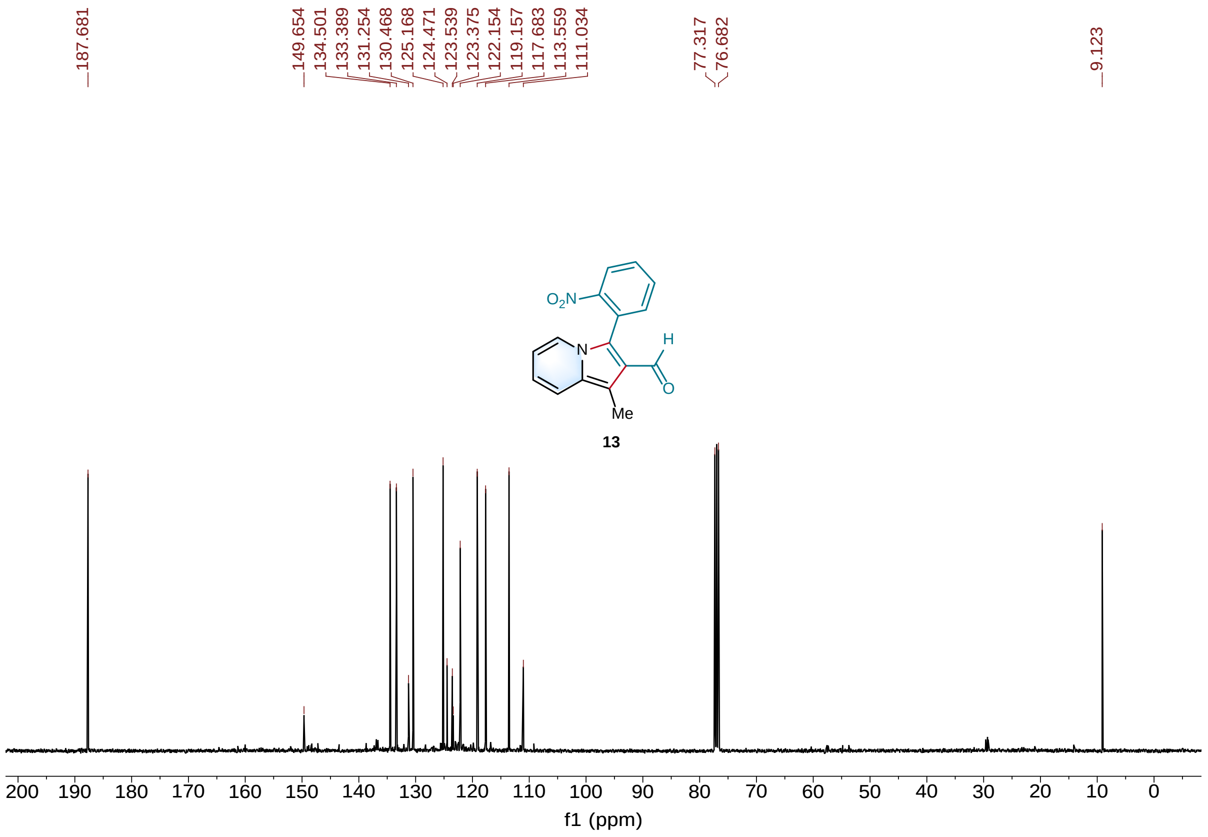

^{1}H NMR spectra of 14 (400 MHz, $CDCl_3$)

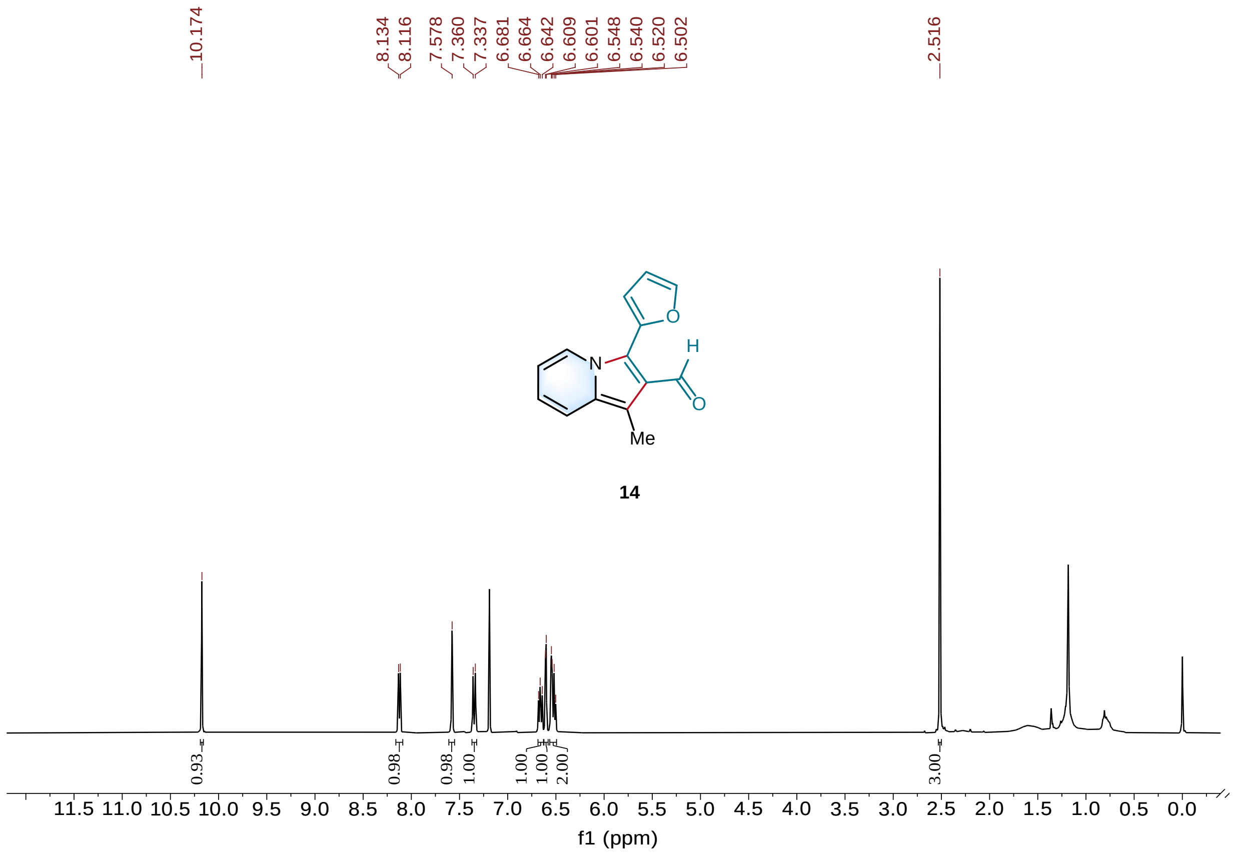

^{13}C NMR spectra of 14 (100 MHz, $CDCl_3$)

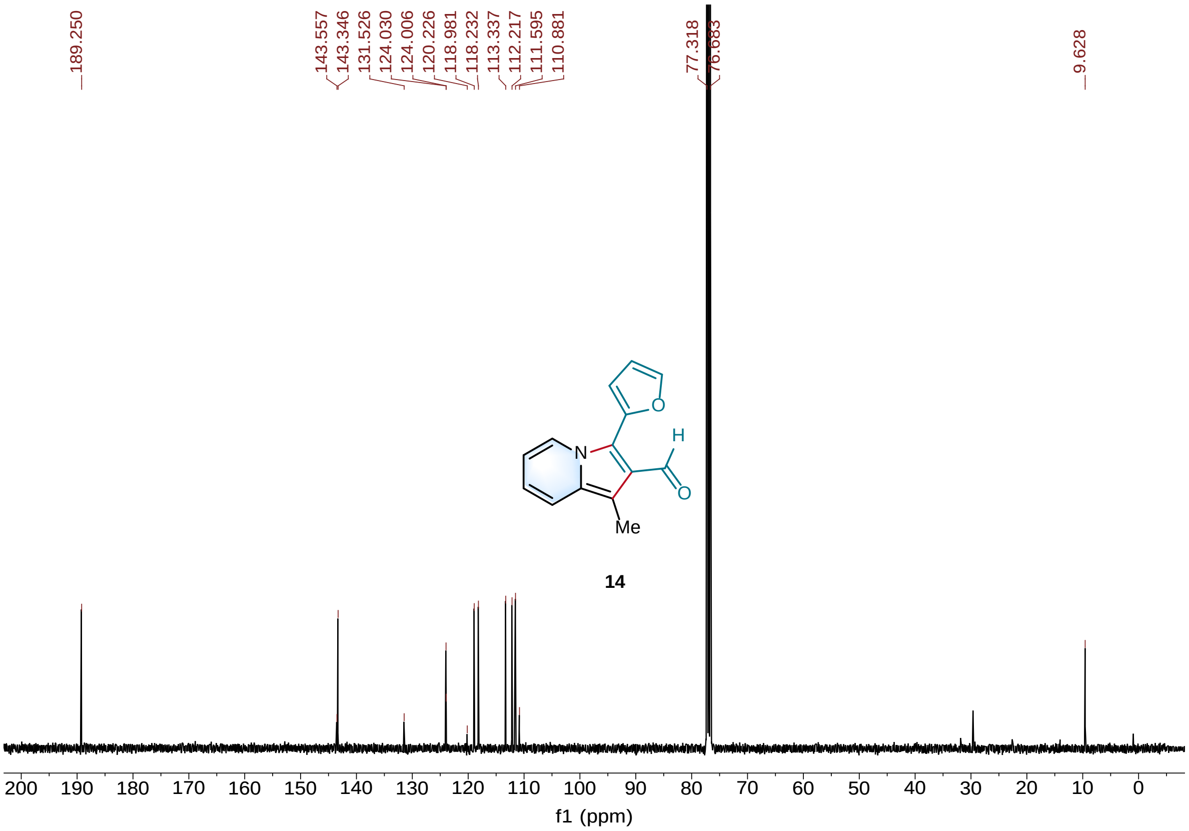

^{1}H NMR spectra of 15 (400 MHz, $CDCl_3$)

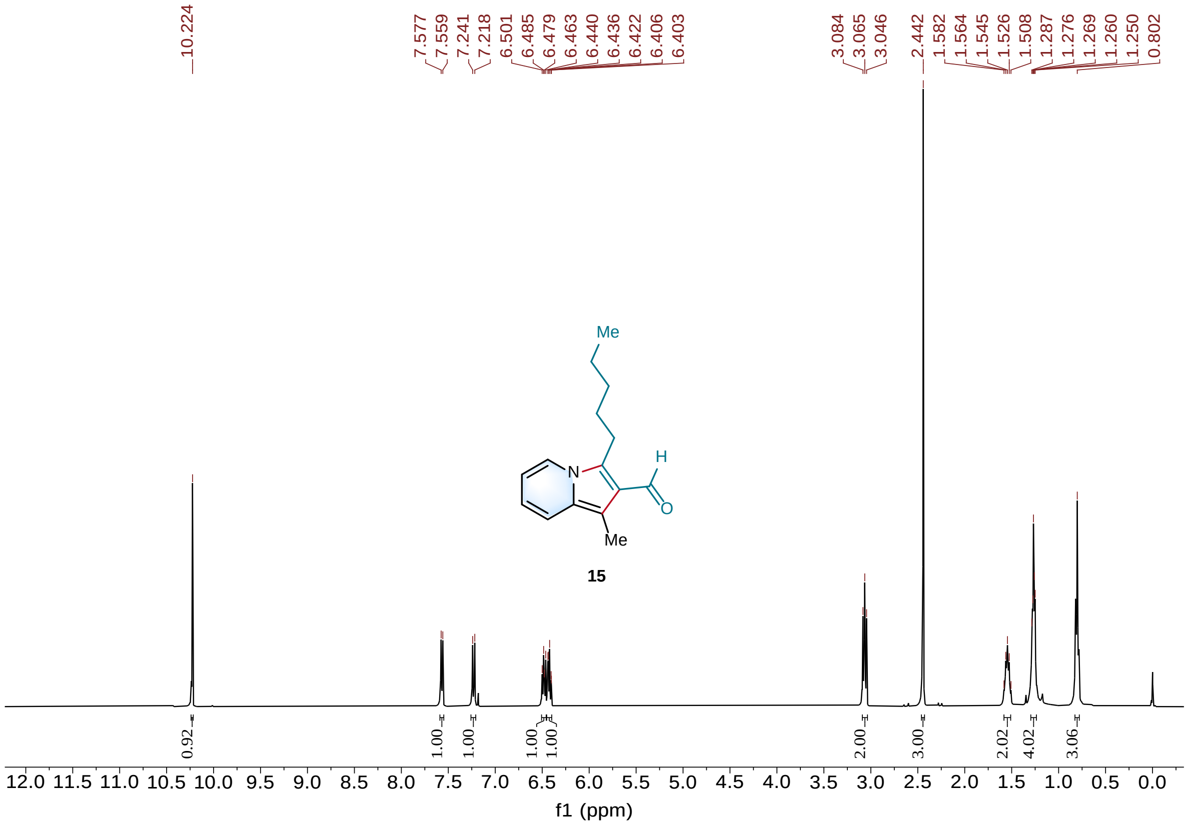

^{13}C NMR spectra of 15 (100 MHz, $CDCl_3$)

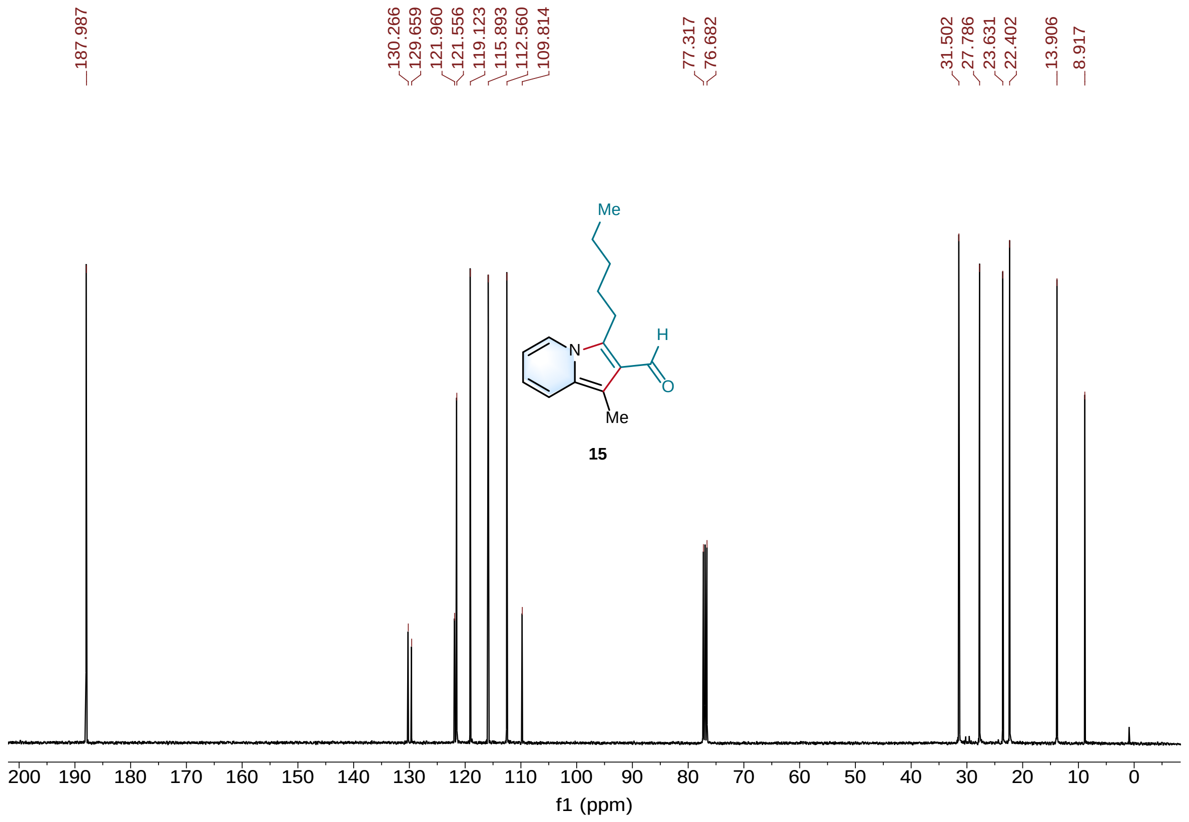

^{1}H NMR spectra of 16 (400 MHz, $CDCl_3$)

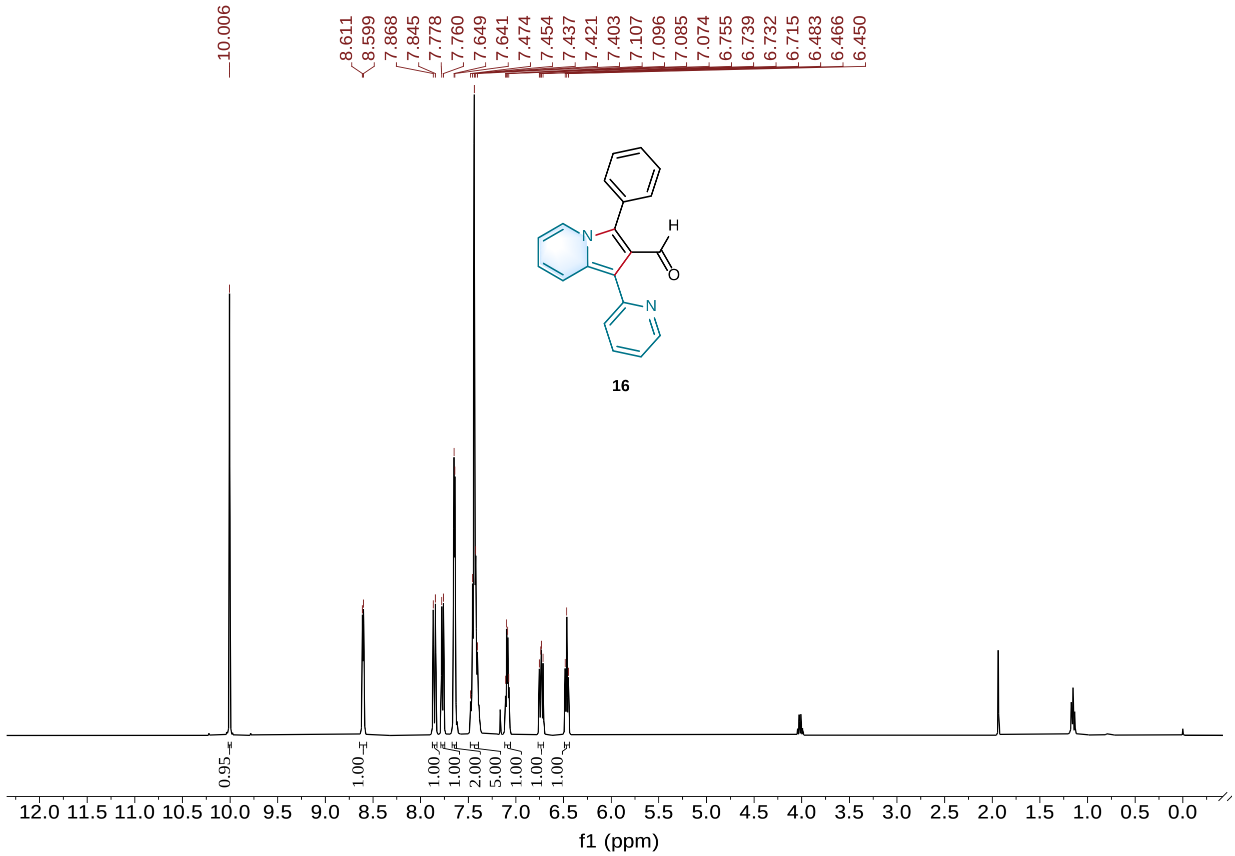

^{13}C NMR spectra of 16 (100 MHz, $CDCl_3$)

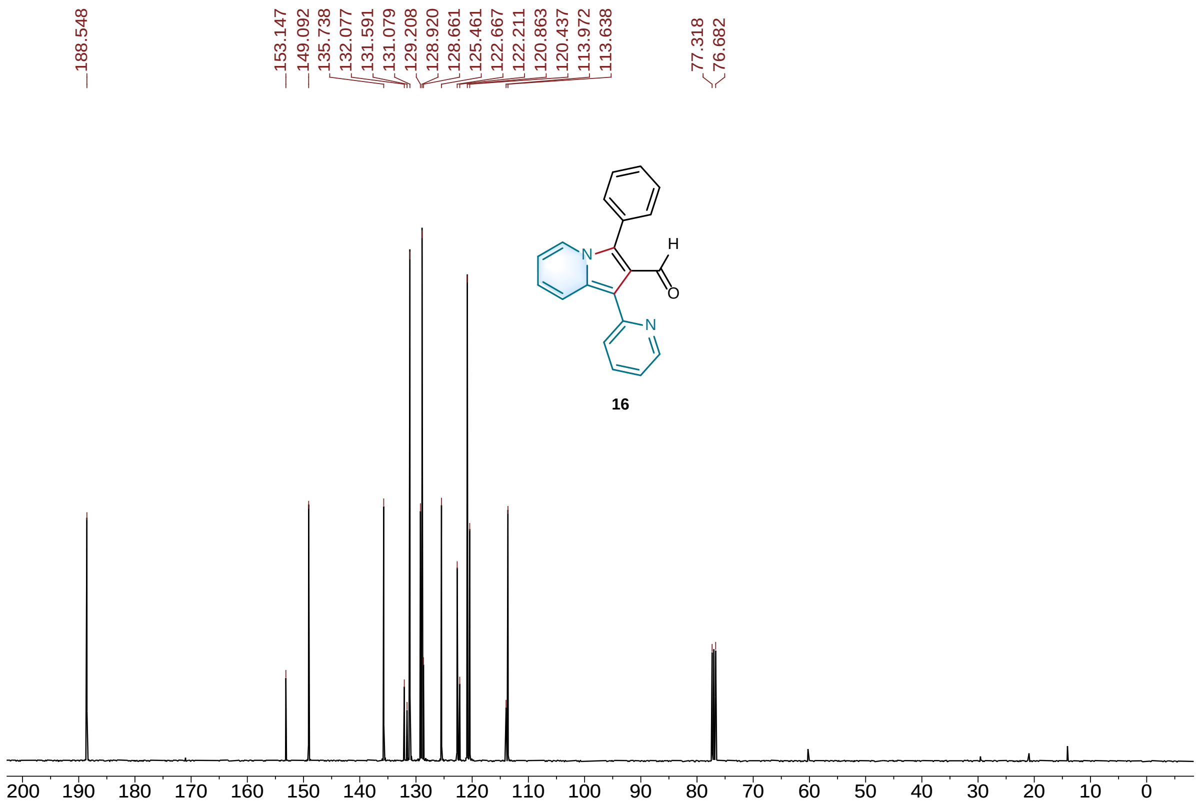

^{1}H NMR spectra of 17 (400 MHz, $CDCl_3$)

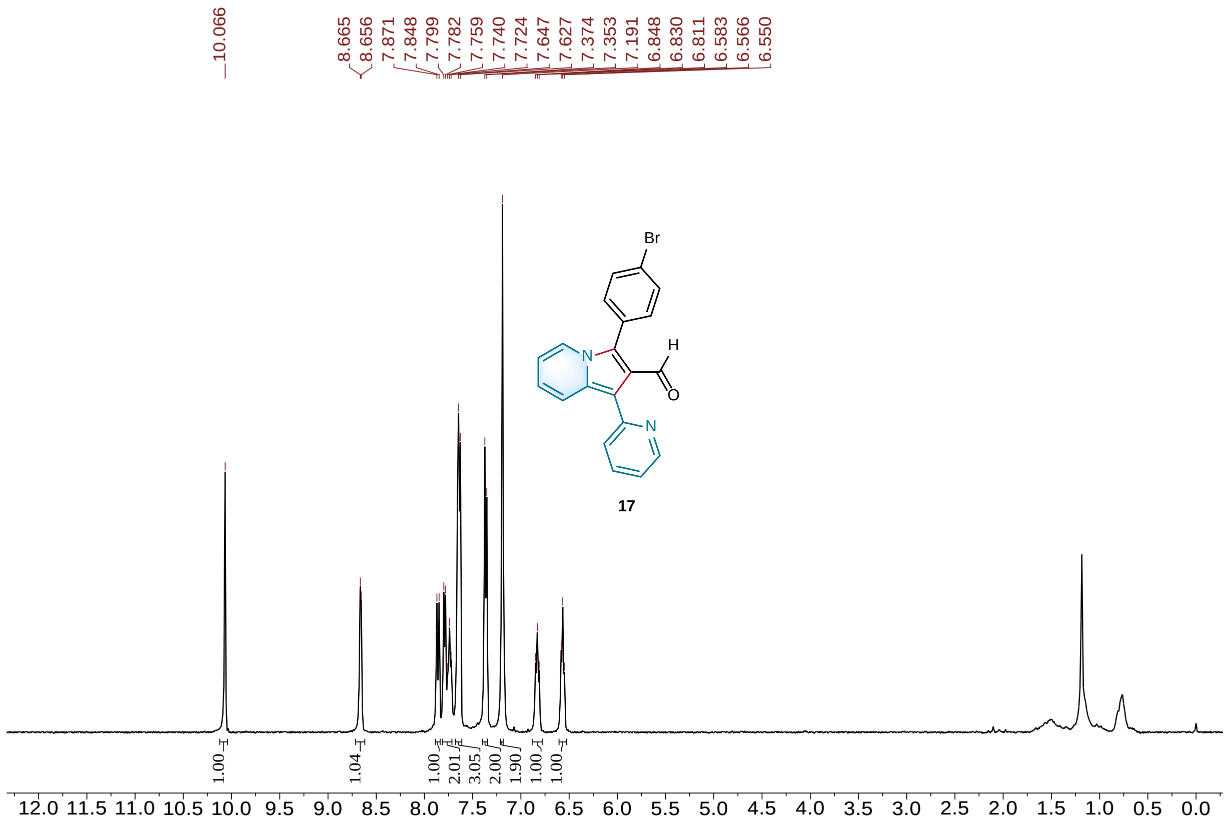

^{13}C NMR spectra of 17 (100 MHz, $CDCl_3$)

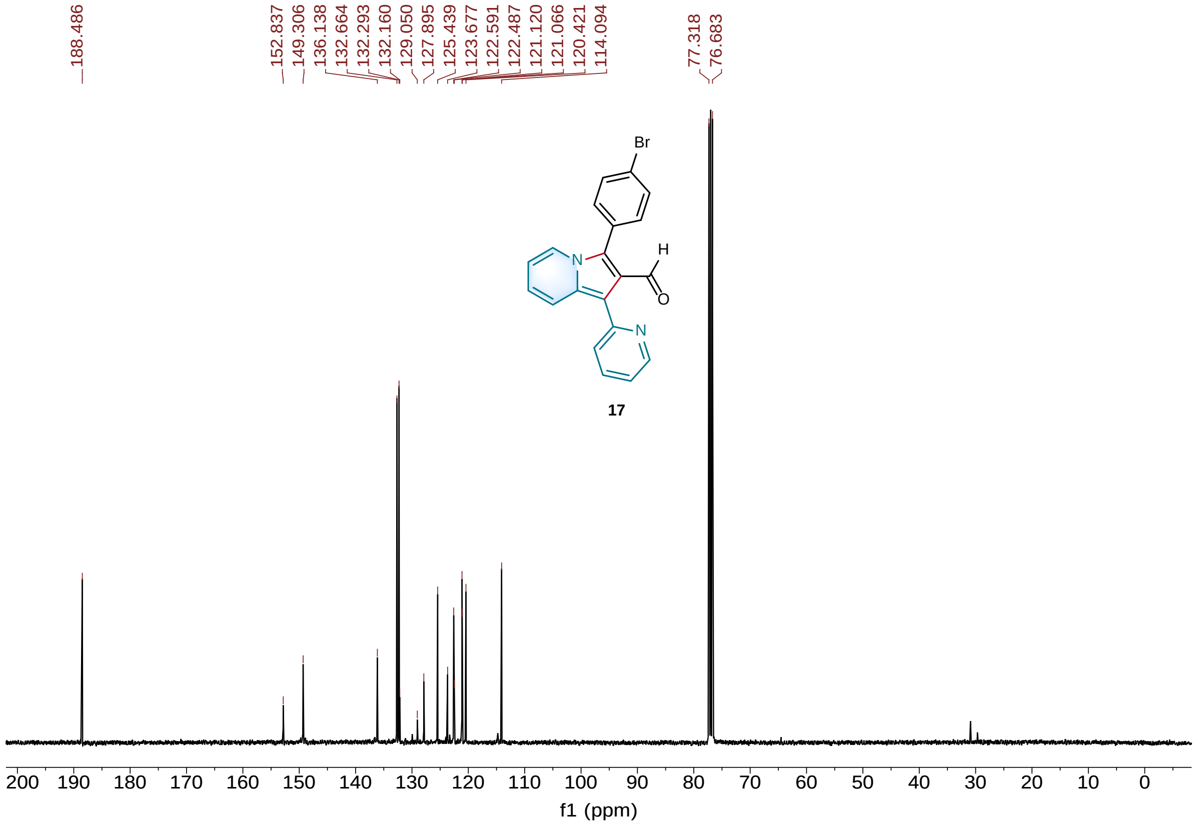

^{1}H NMR spectra of 18 (400 MHz, $CDCl_3$)

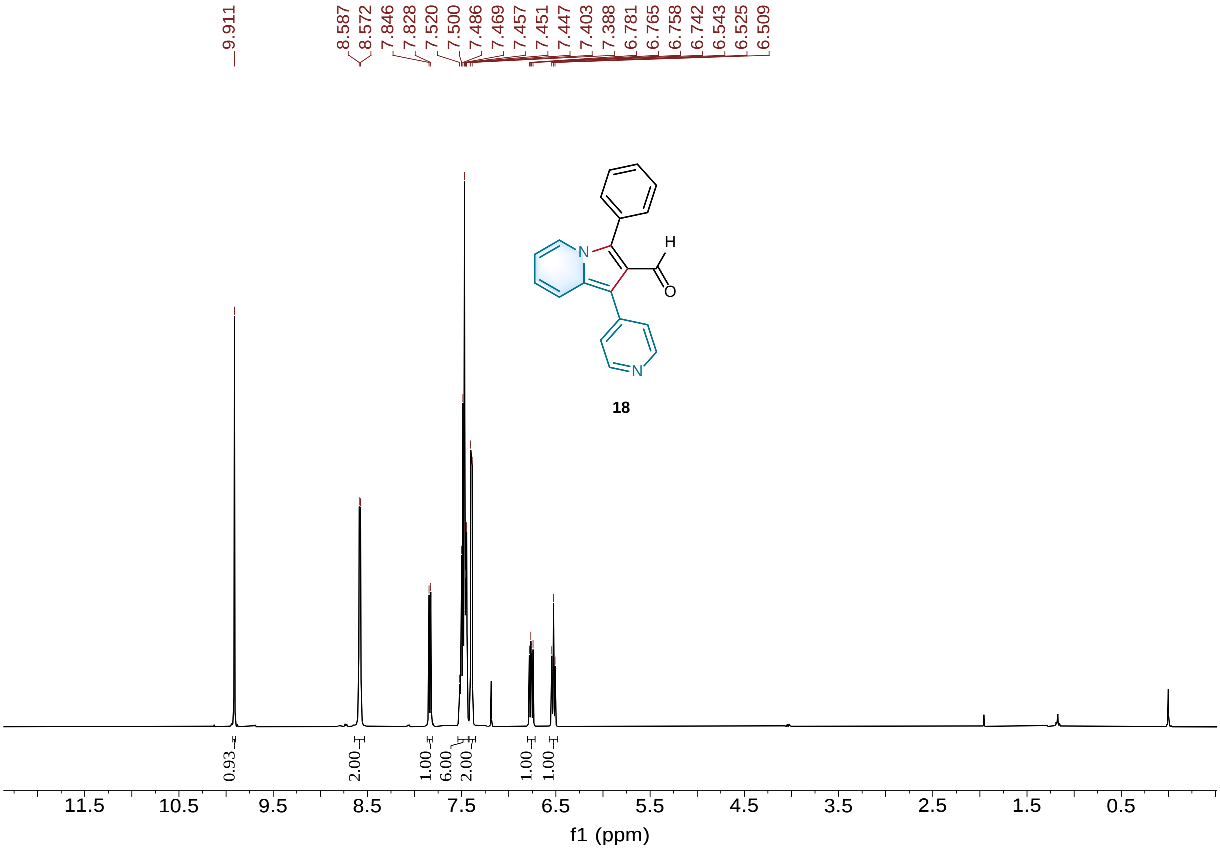

^{13}C NMR spectra of 18 (100 MHz, $CDCl_3$)

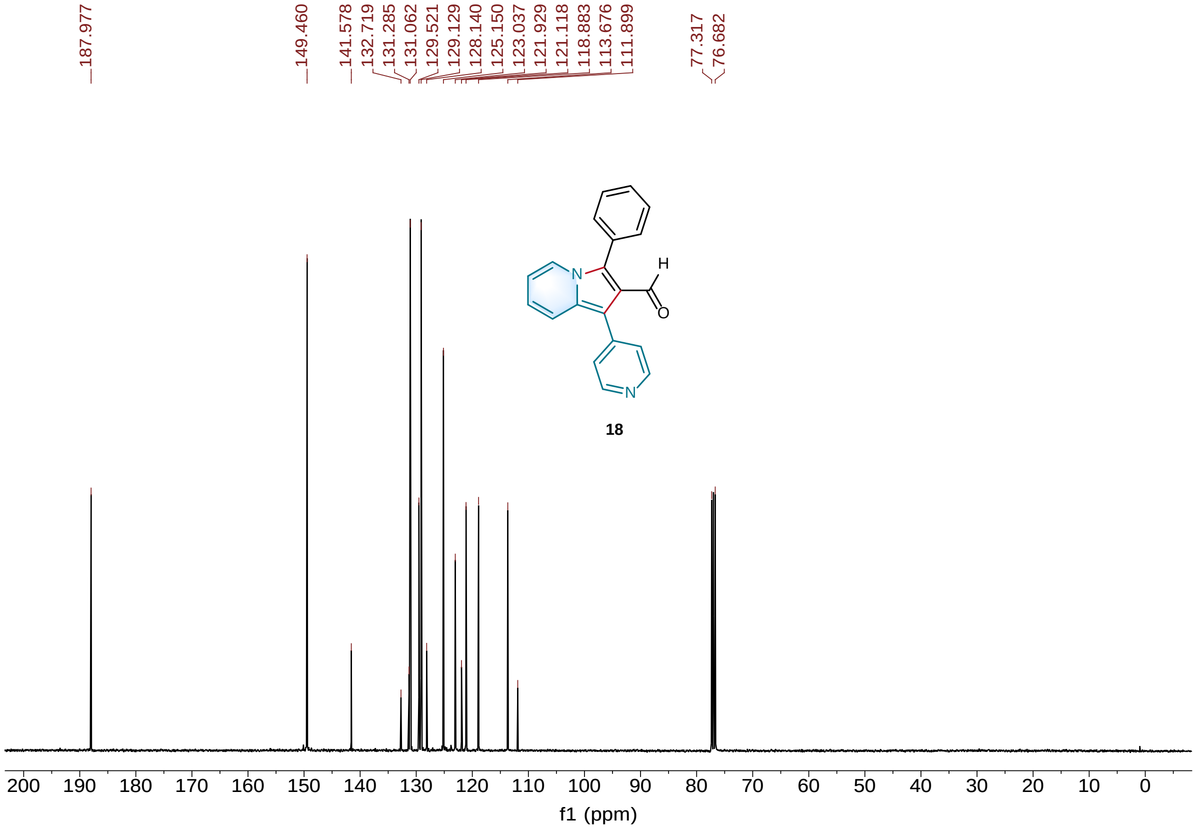

^{1}H NMR spectra of 19 (400 MHz, $CDCl_3$)

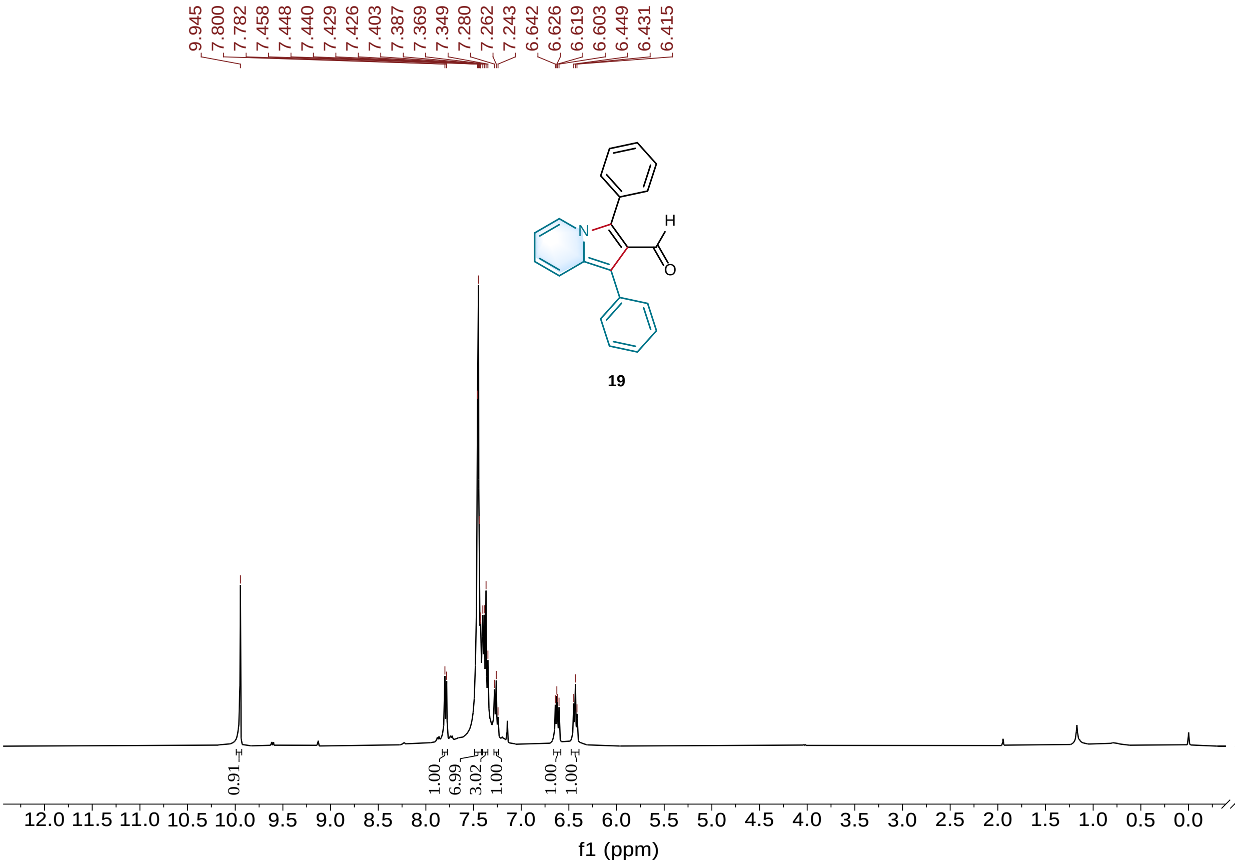

^{13}C NMR spectra of 19 (100 MHz, $CDCl_3$)

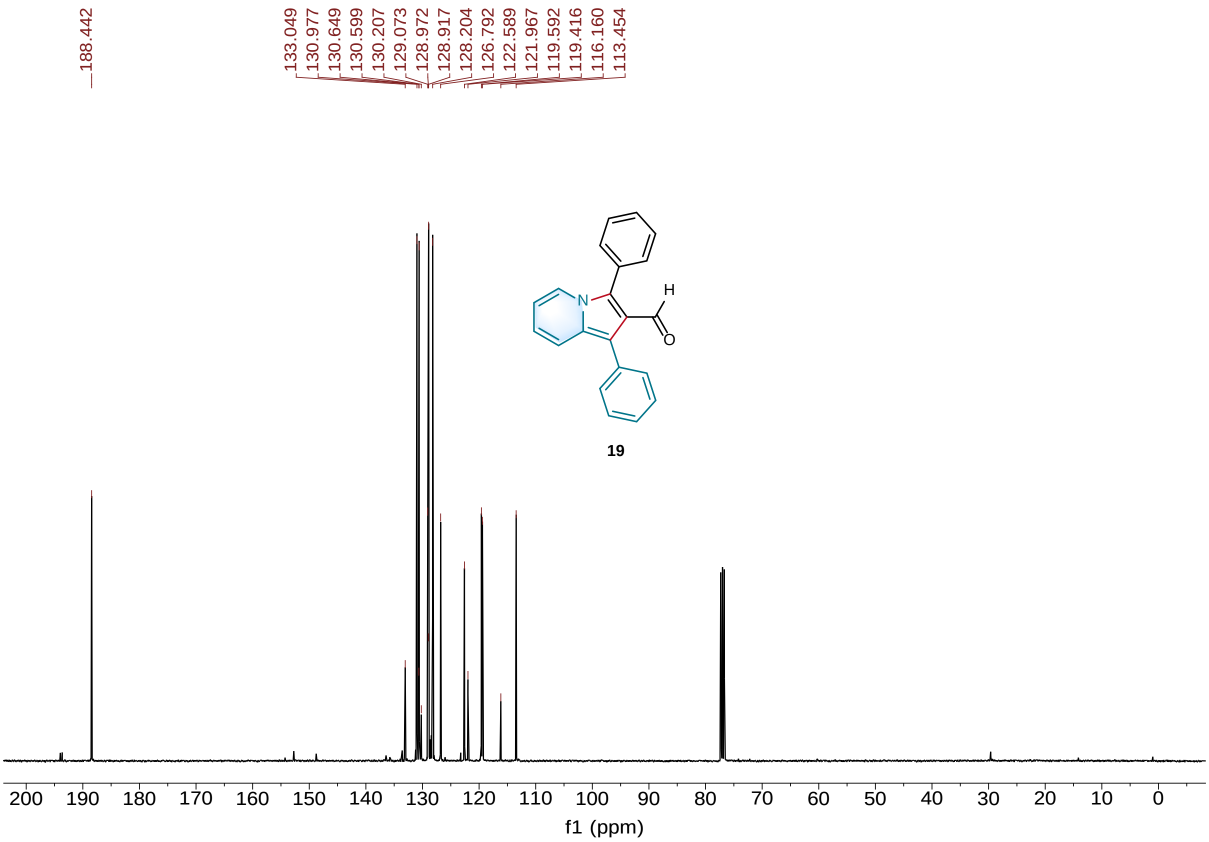

^{1}H NMR spectra of 20 (400 MHz, $CDCl_3$)

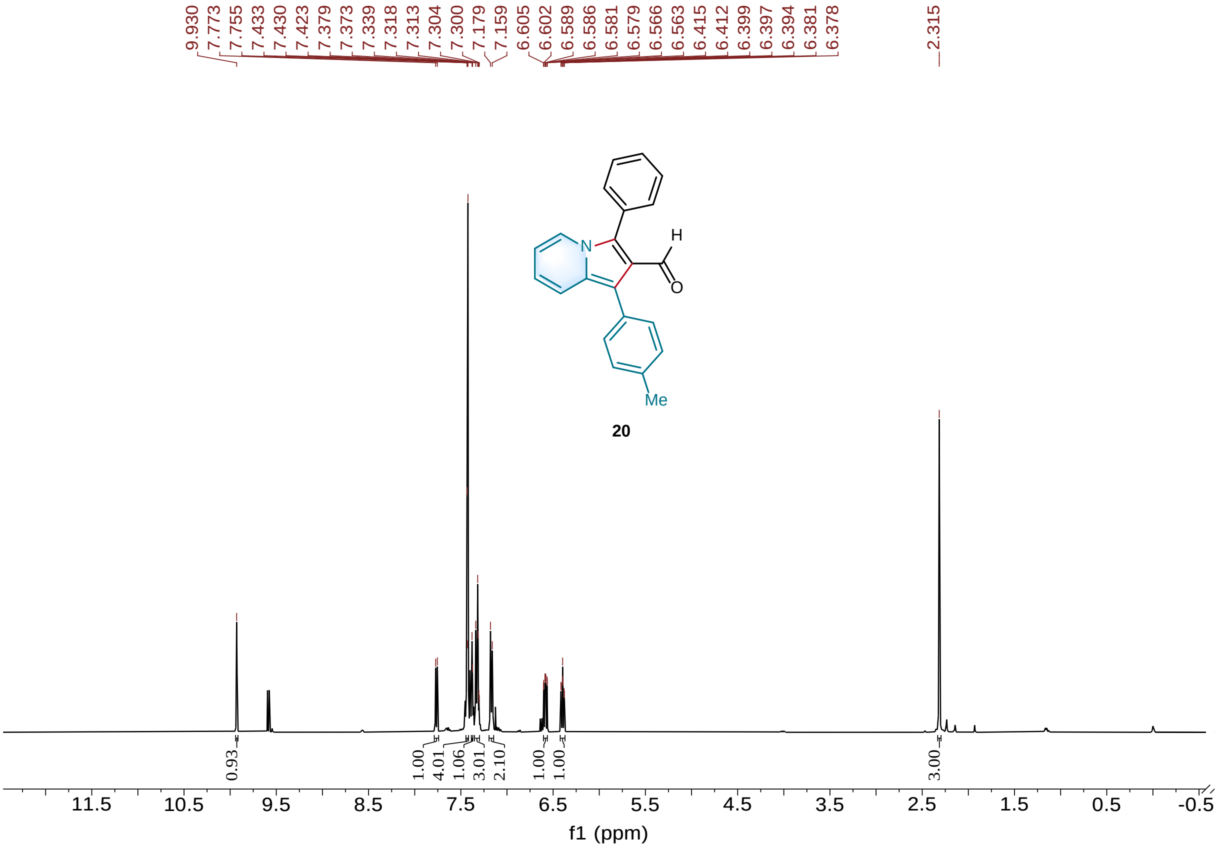

^{13}C NMR spectra of 20 (100 MHz, $CDCl_3$)

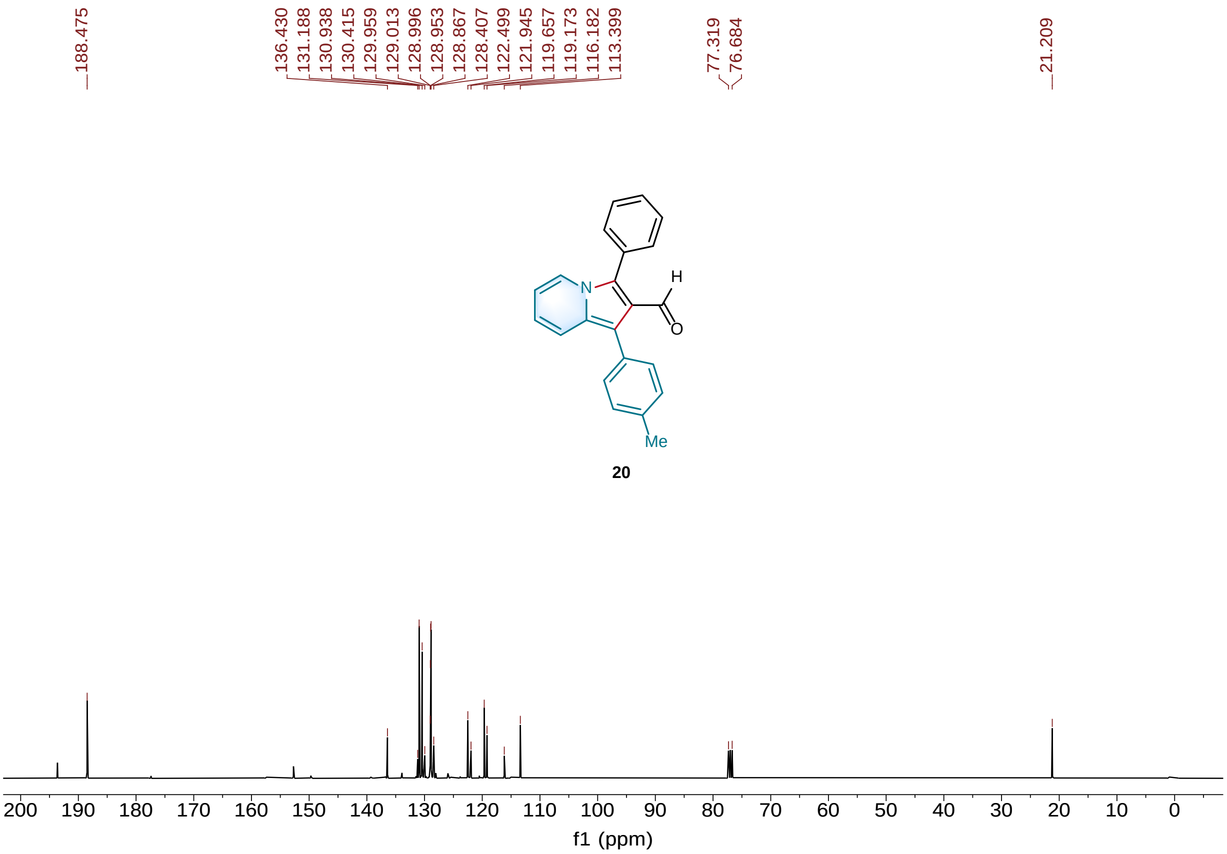

^{1}H NMR spectra of 21 (400 MHz, $CDCl_3$)

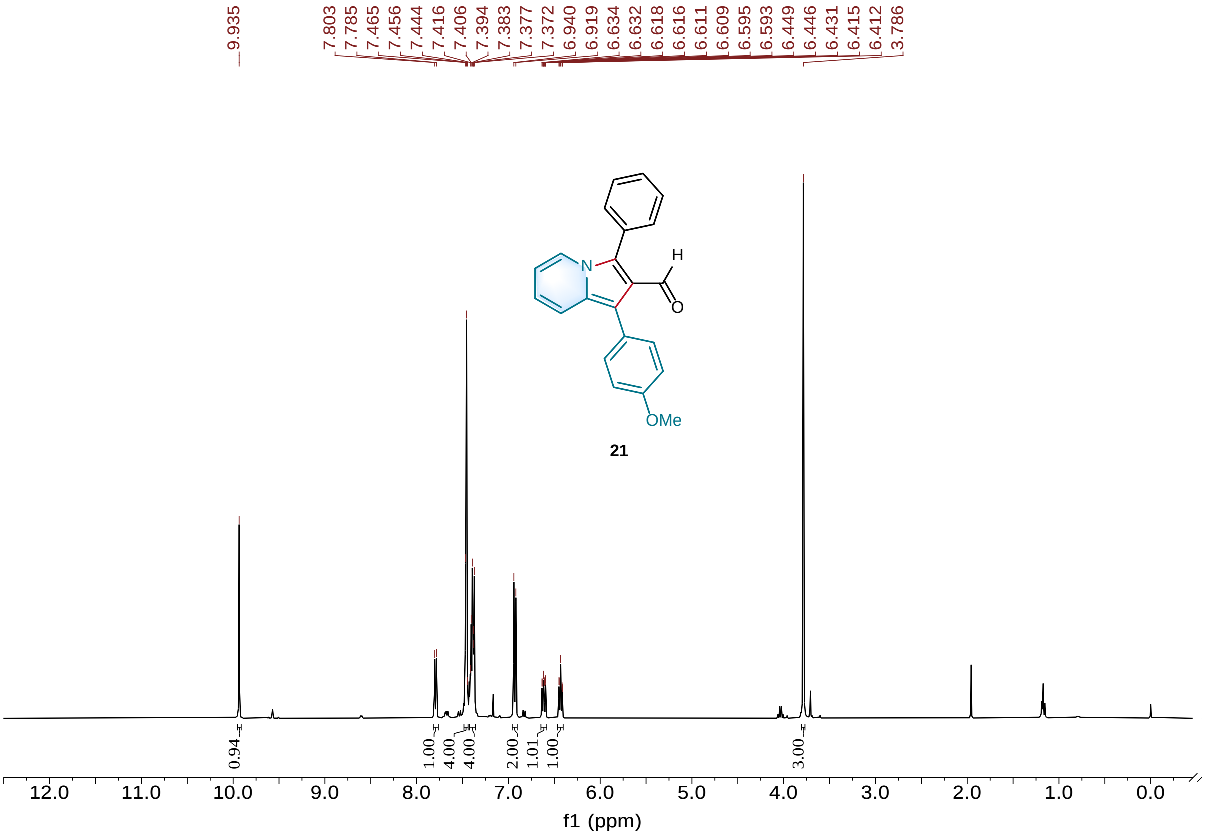

^{13}C NMR spectra of 21 (100 MHz, $CDCl_3$)

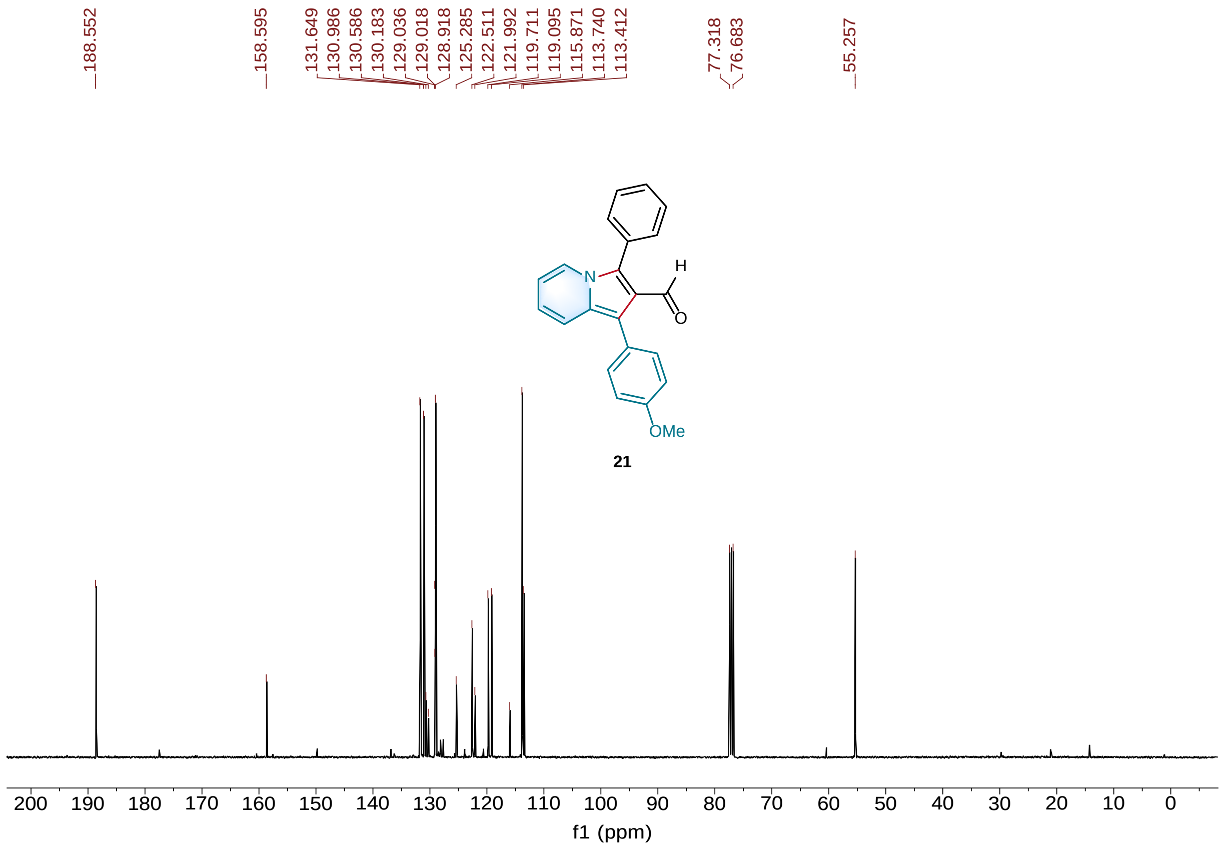

^{1}H NMR spectra of 22 (400 MHz, $CDCl_3$)

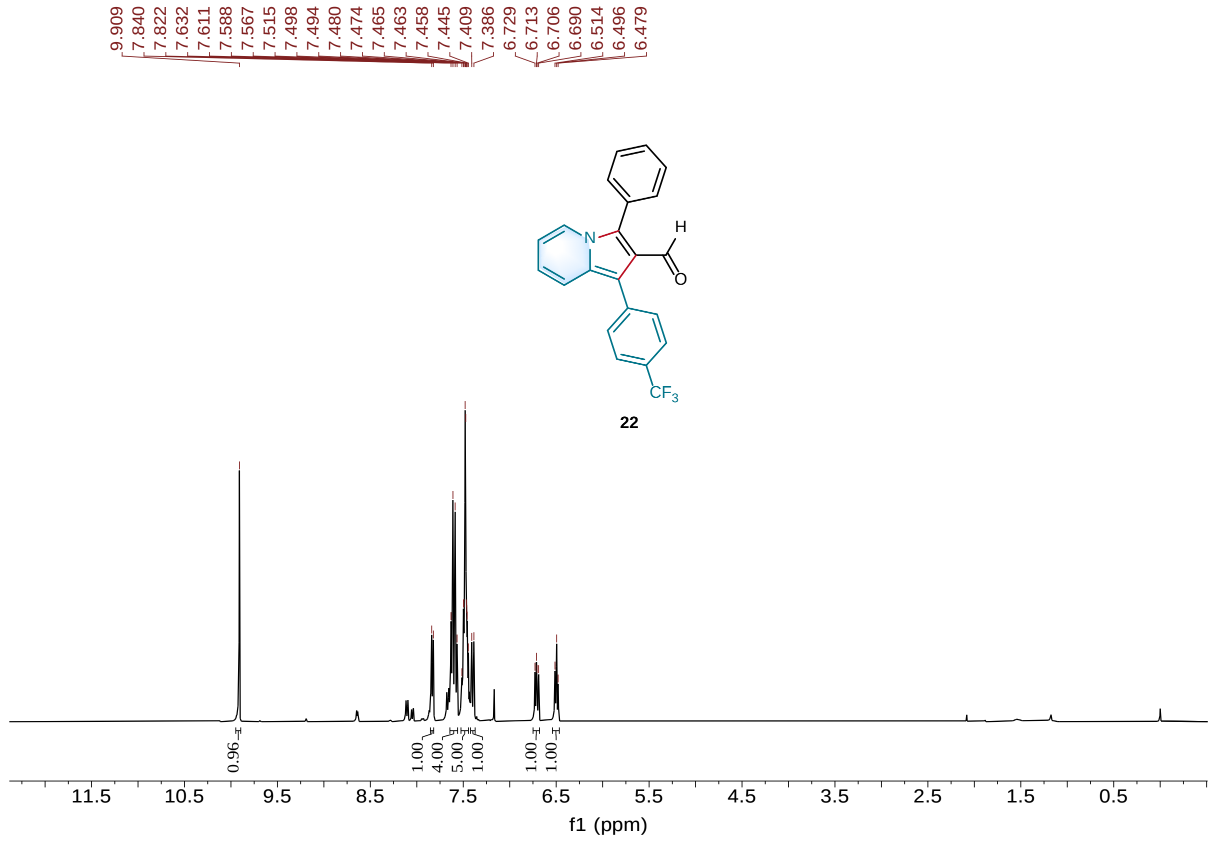

^{13}C NMR spectra of 22 (100 MHz, $CDCl_3$)

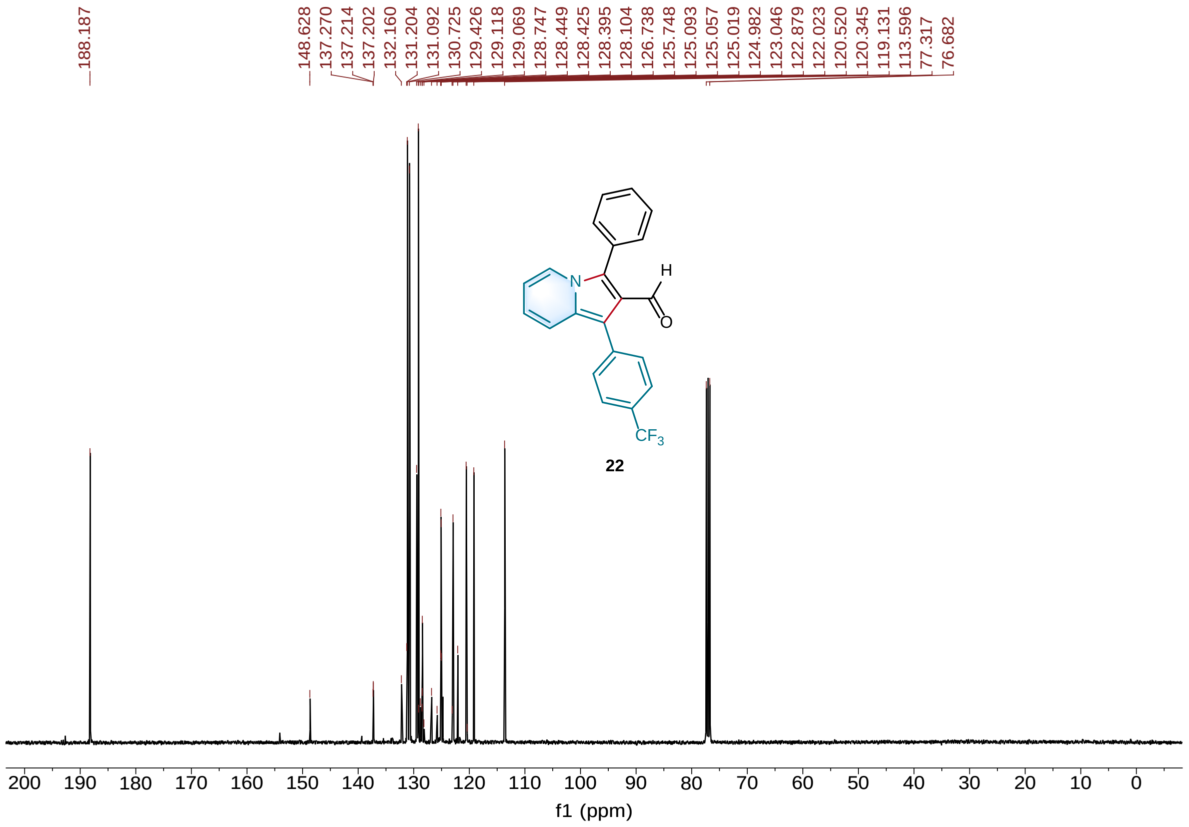

^{19}F NMR spectra of 22 (377 MHz, $CDCl_3$)

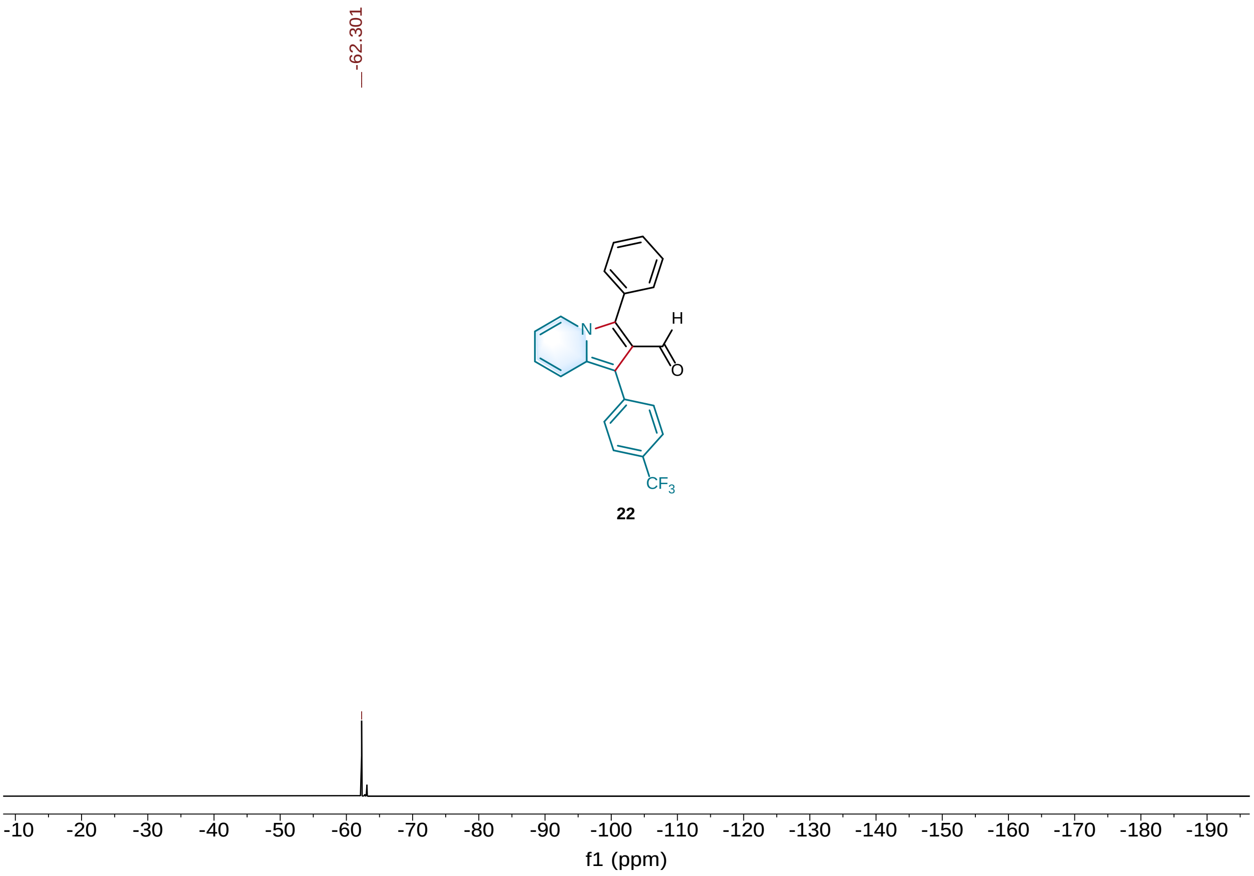

^{1}H NMR spectra of 23 (400 MHz, $CDCl_3$)

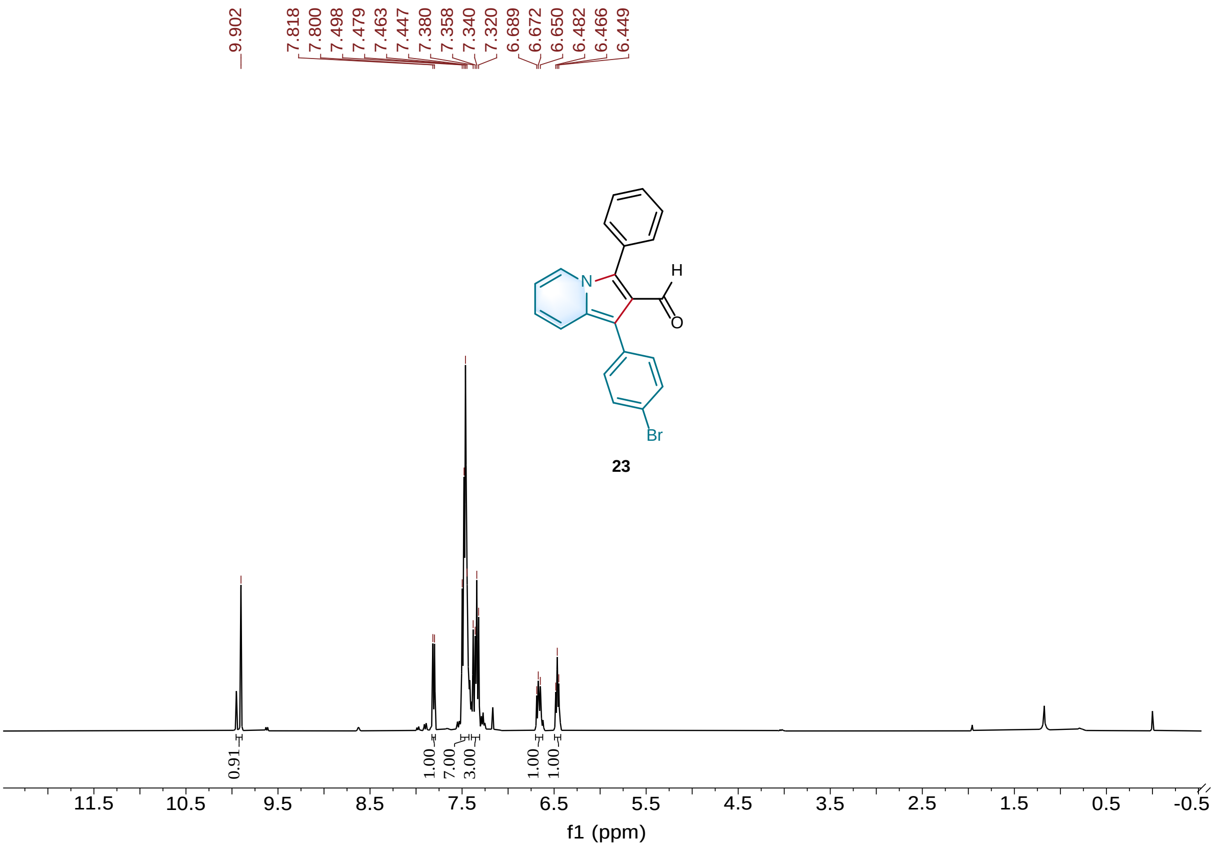

^{13}C NMR spectra of 23 (100 MHz, $CDCl_3$)

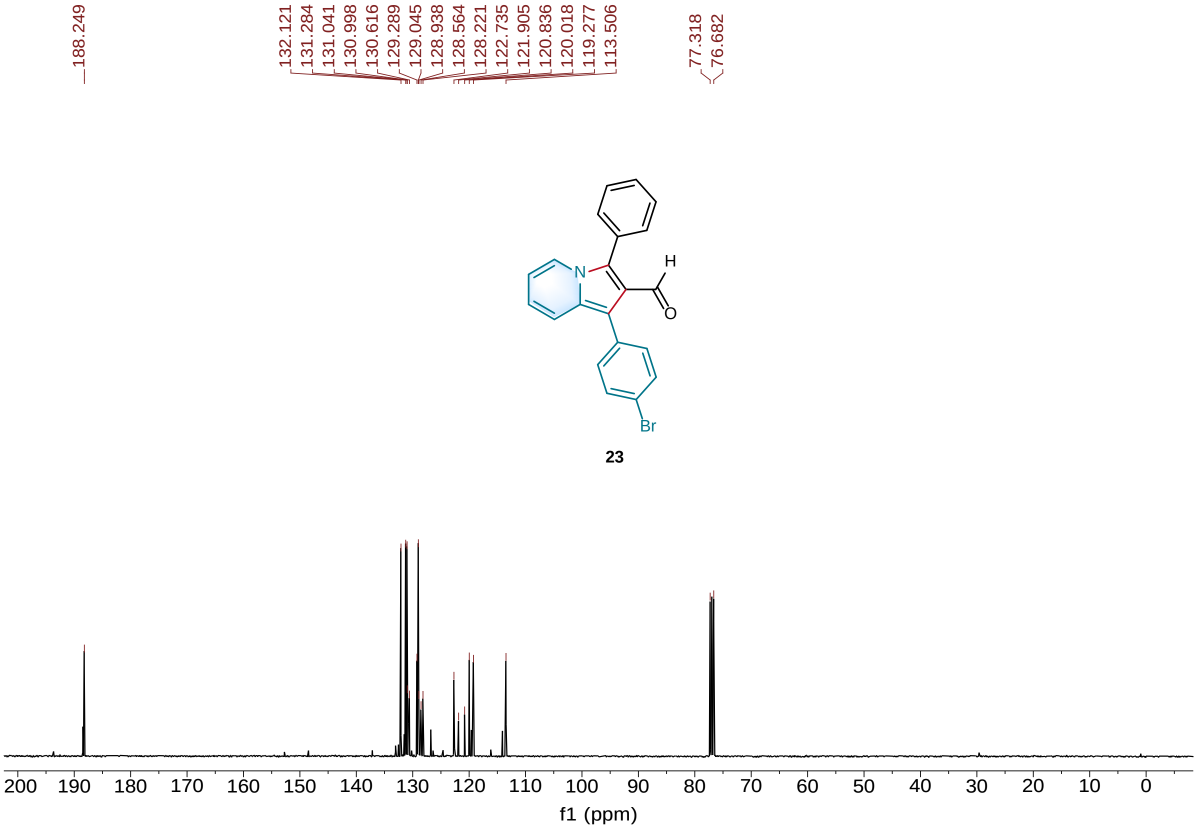

^{1}H NMR spectra of 24 (400 MHz, $CDCl_3$)

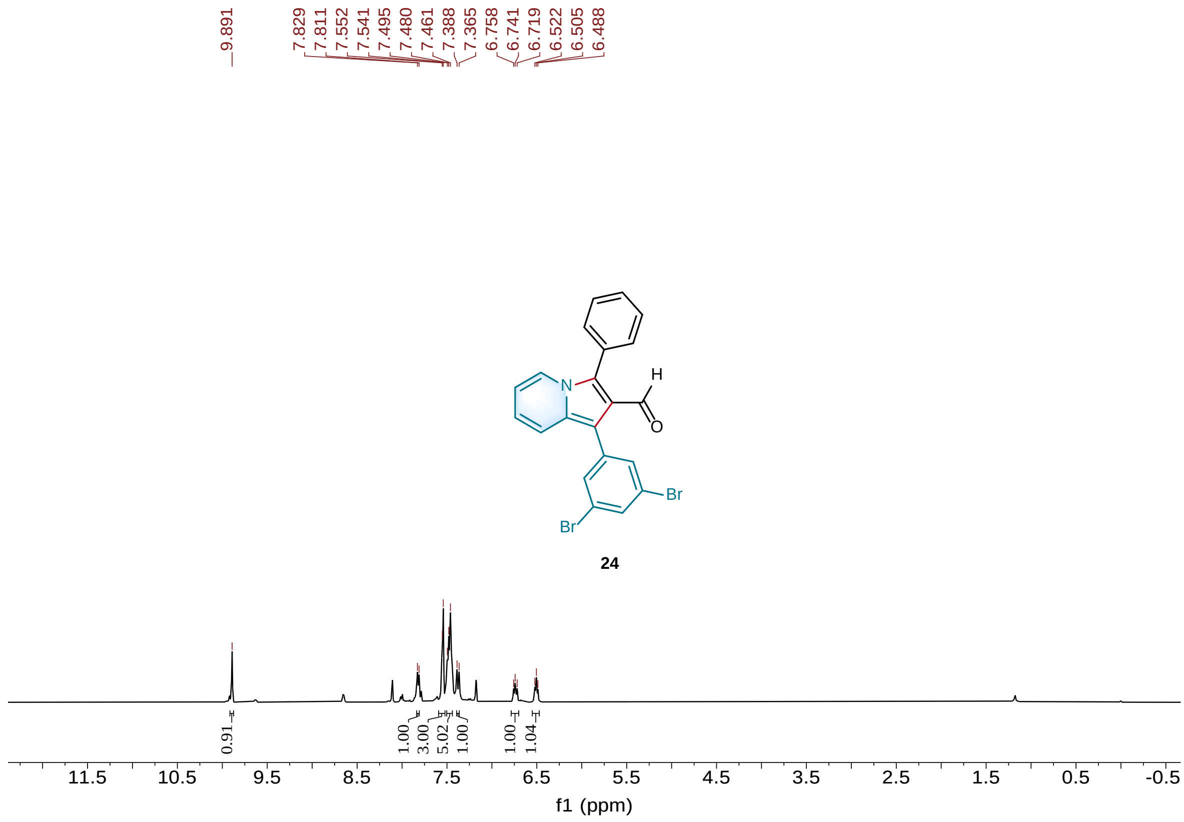

^{13}C NMR spectra of 24 (100 MHz, $CDCl_3$)

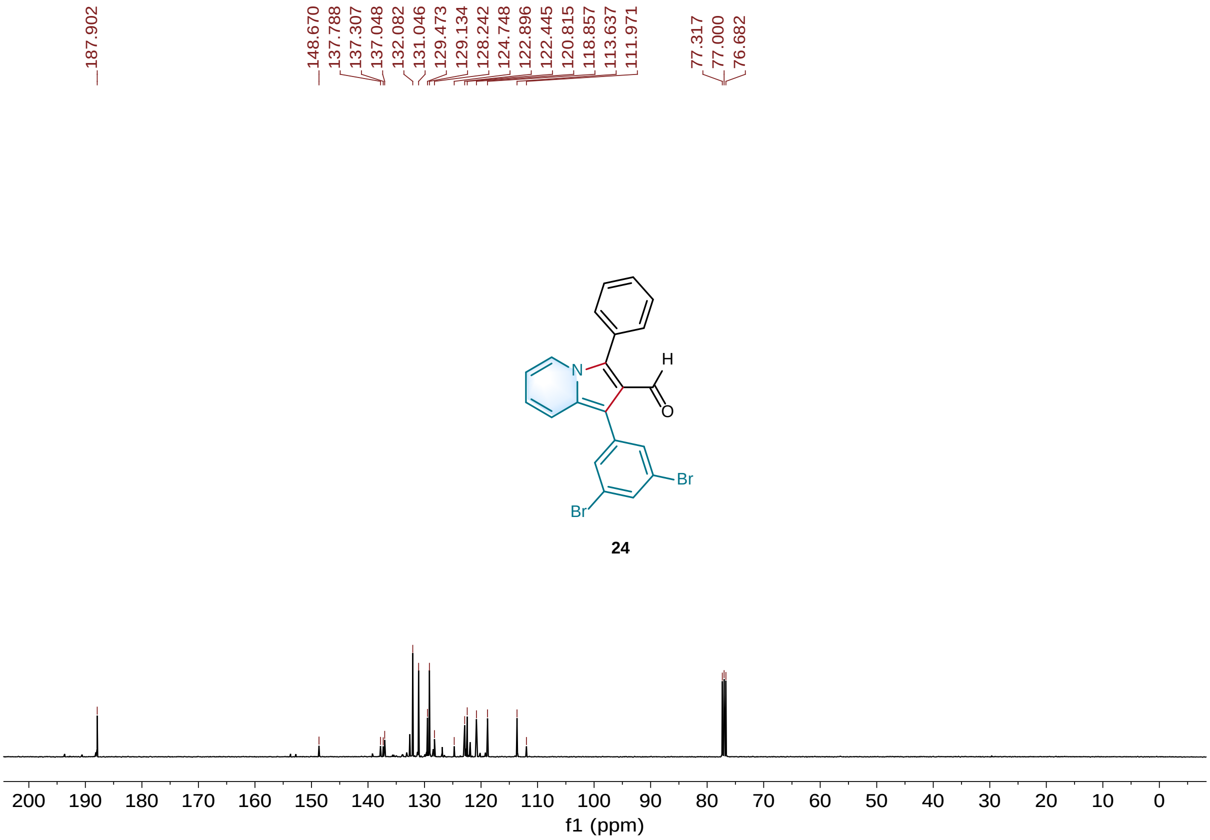

^{1}H NMR spectra of 25 (400 MHz, $CDCl_3$)

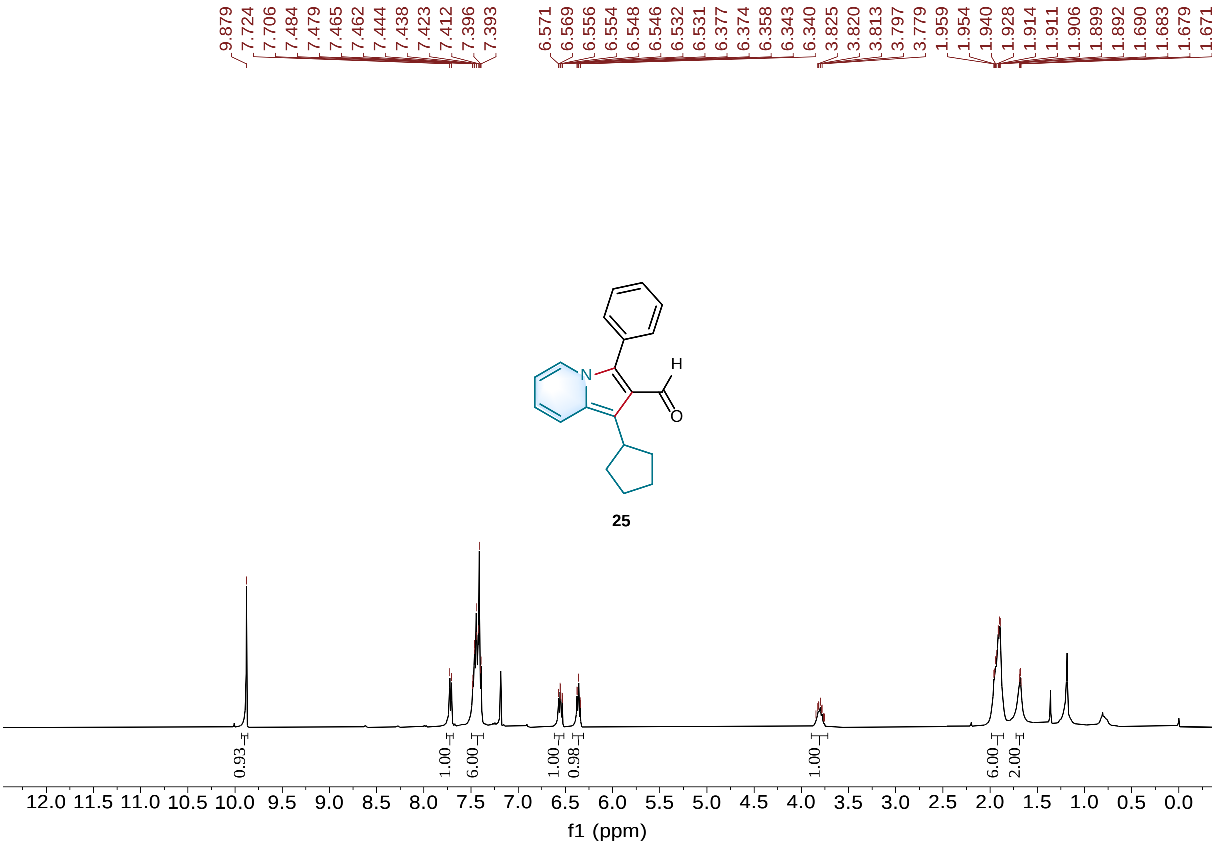

^{13}C NMR spectra of 25 (100 MHz, $CDCl_3$)

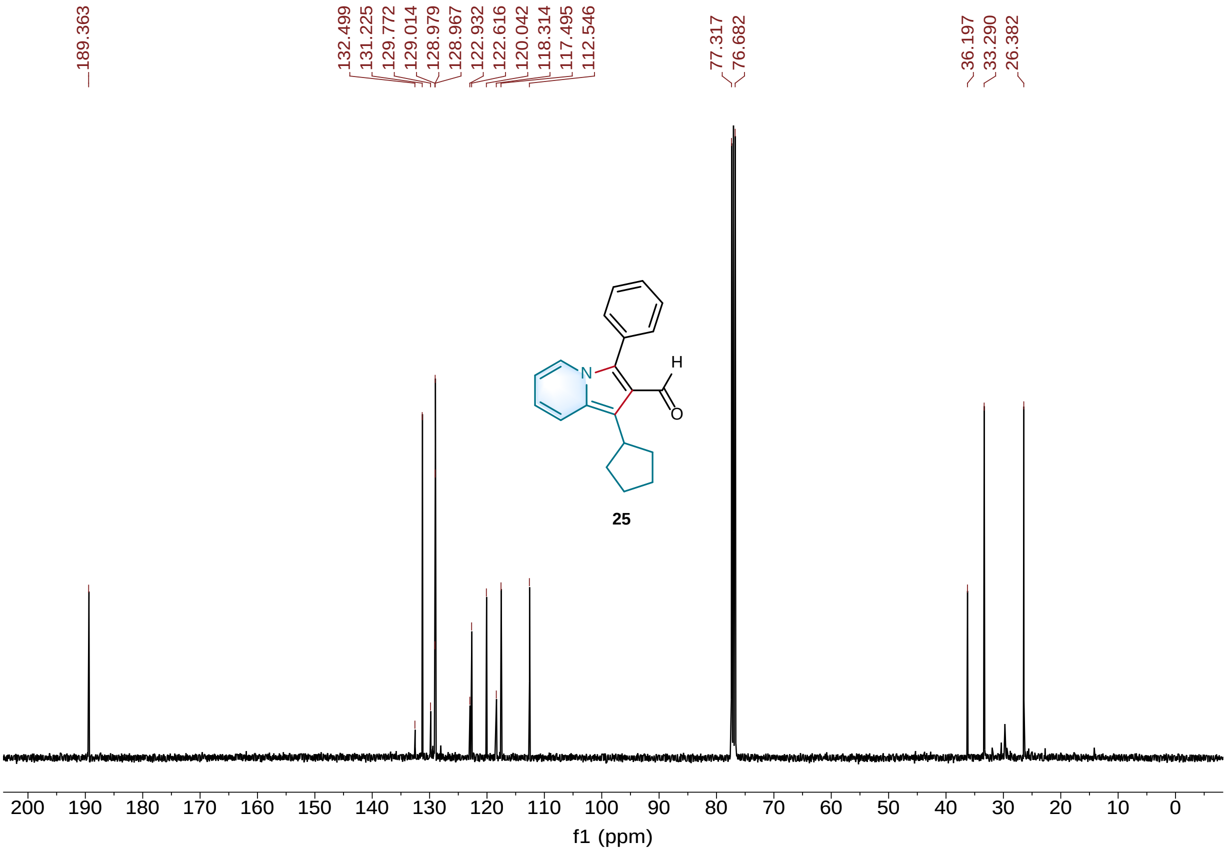

^{1}H NMR spectra of 26 (400 MHz, $CDCl_3$)

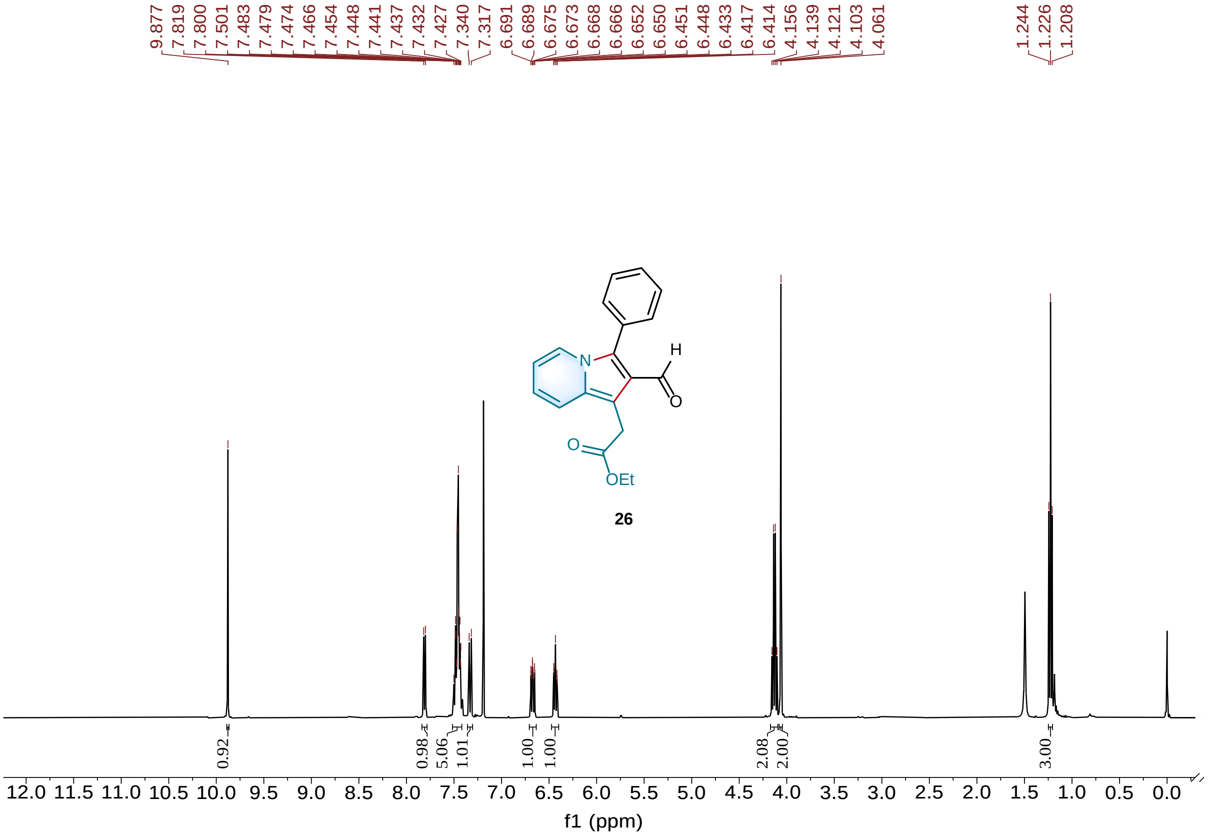

^{13}C NMR spectra of 26 (100 MHz, $CDCl_3$)

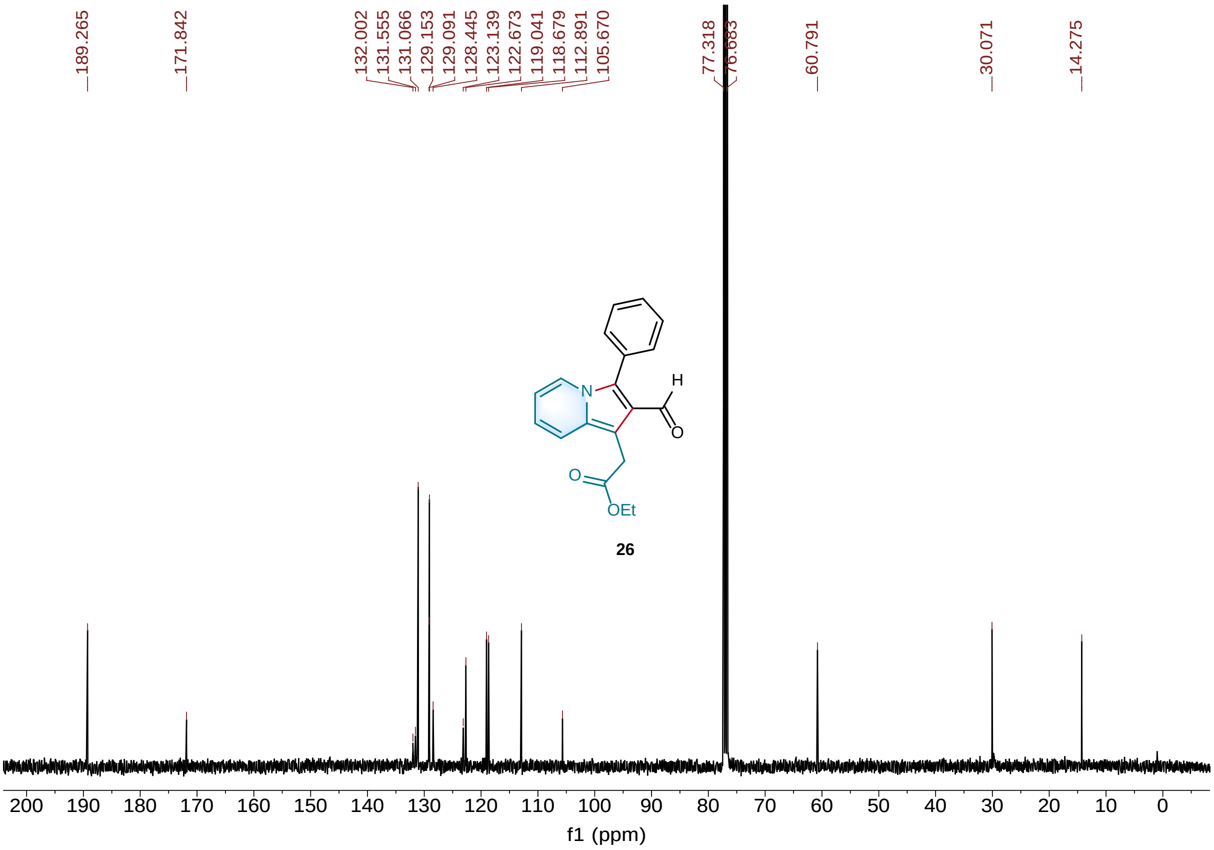

^{1}H NMR spectra of 27 (400 MHz, $CDCl_3$)

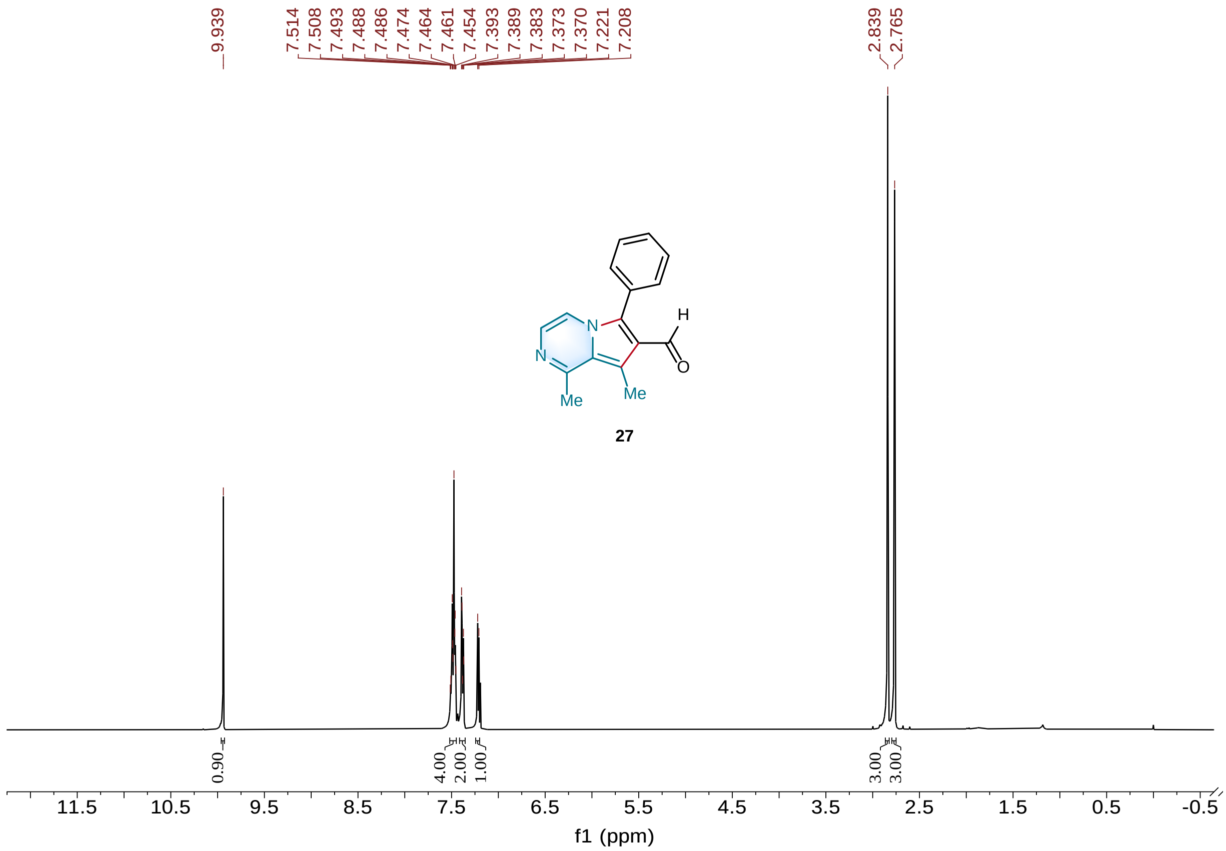

^{13}C NMR spectra of 27 (100 MHz, $CDCl_3$)

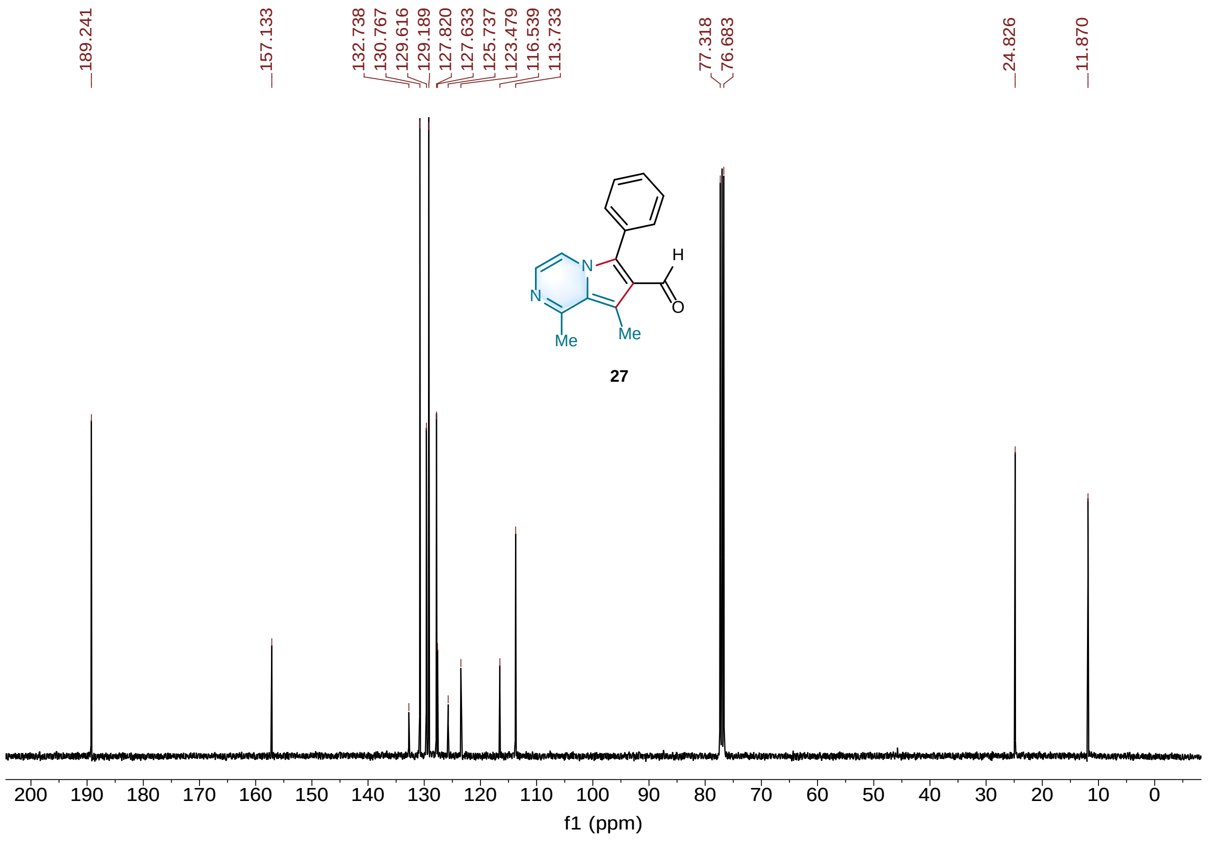

^{1}H NMR spectra of 28 (400 MHz, CDCl$_3$)

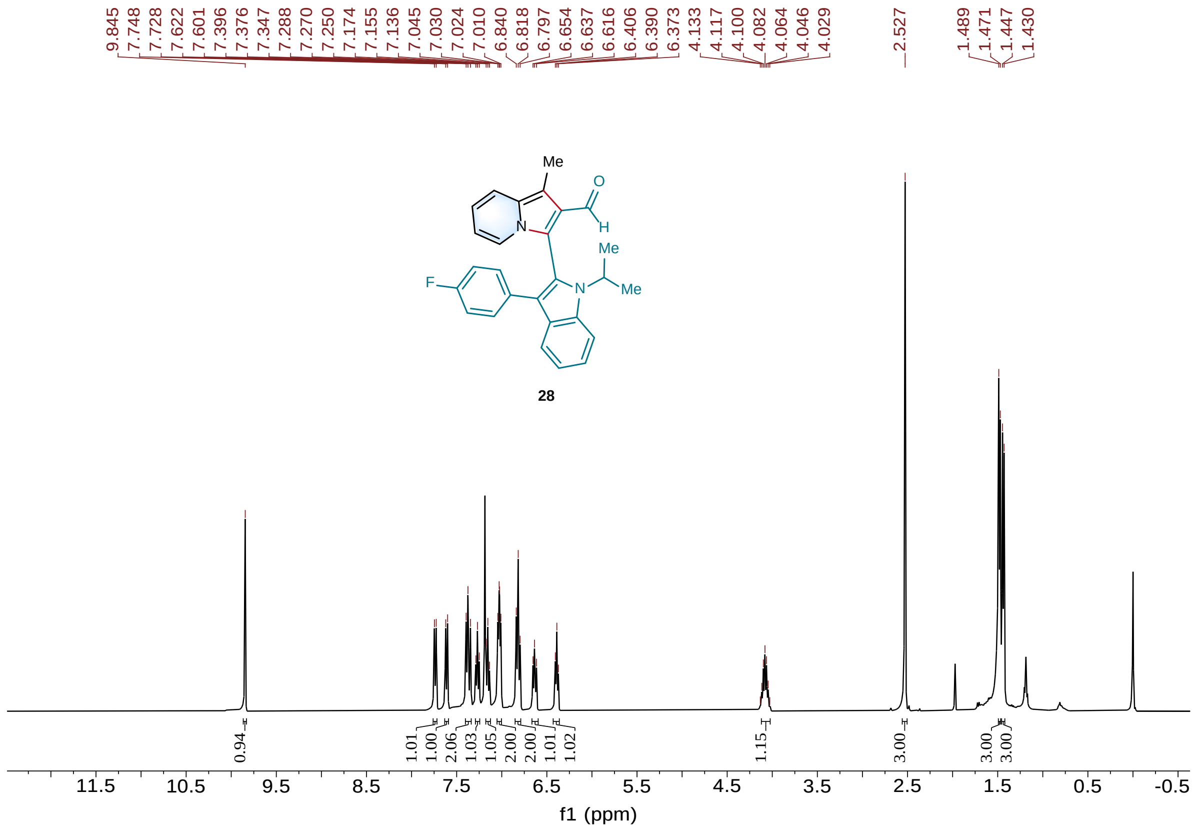

^{13}C NMR spectra of 28 (100 MHz, CDCl$_3$)

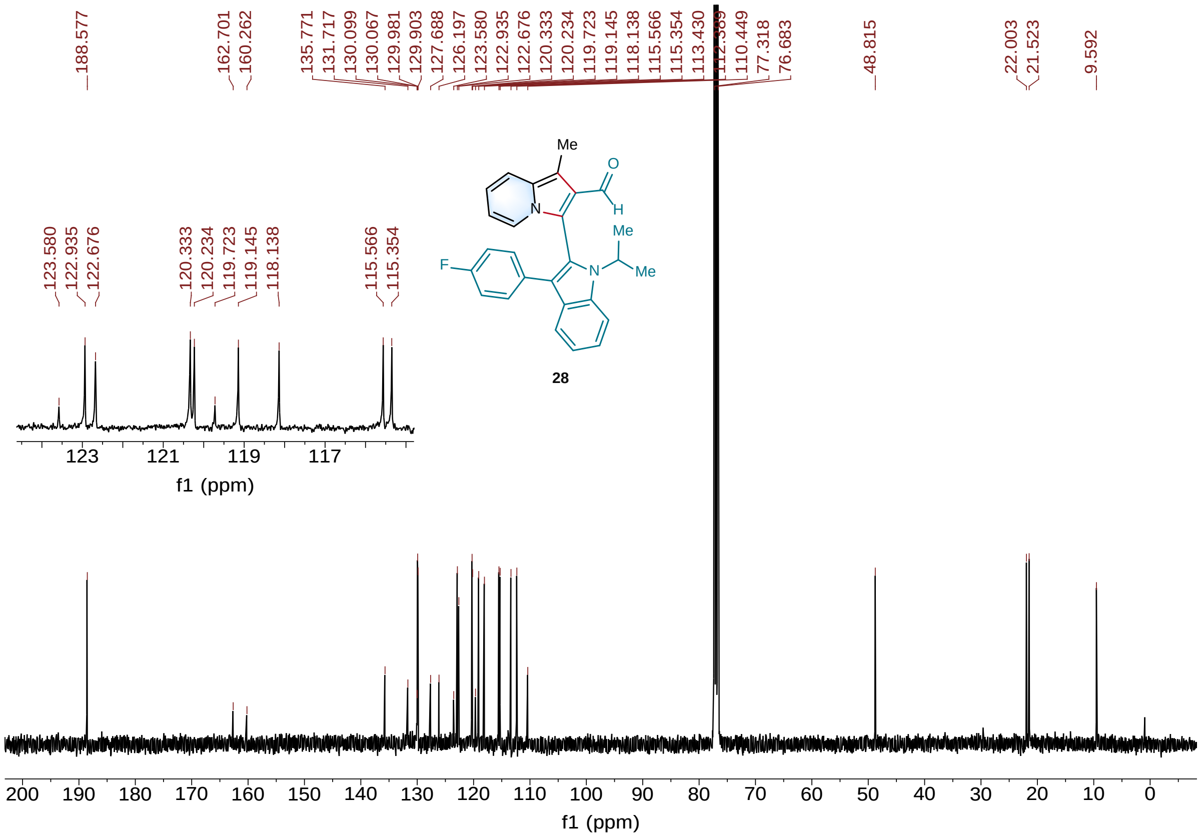

^{19}F NMR spectra of 28 (377 MHz, $CDCl_3$)

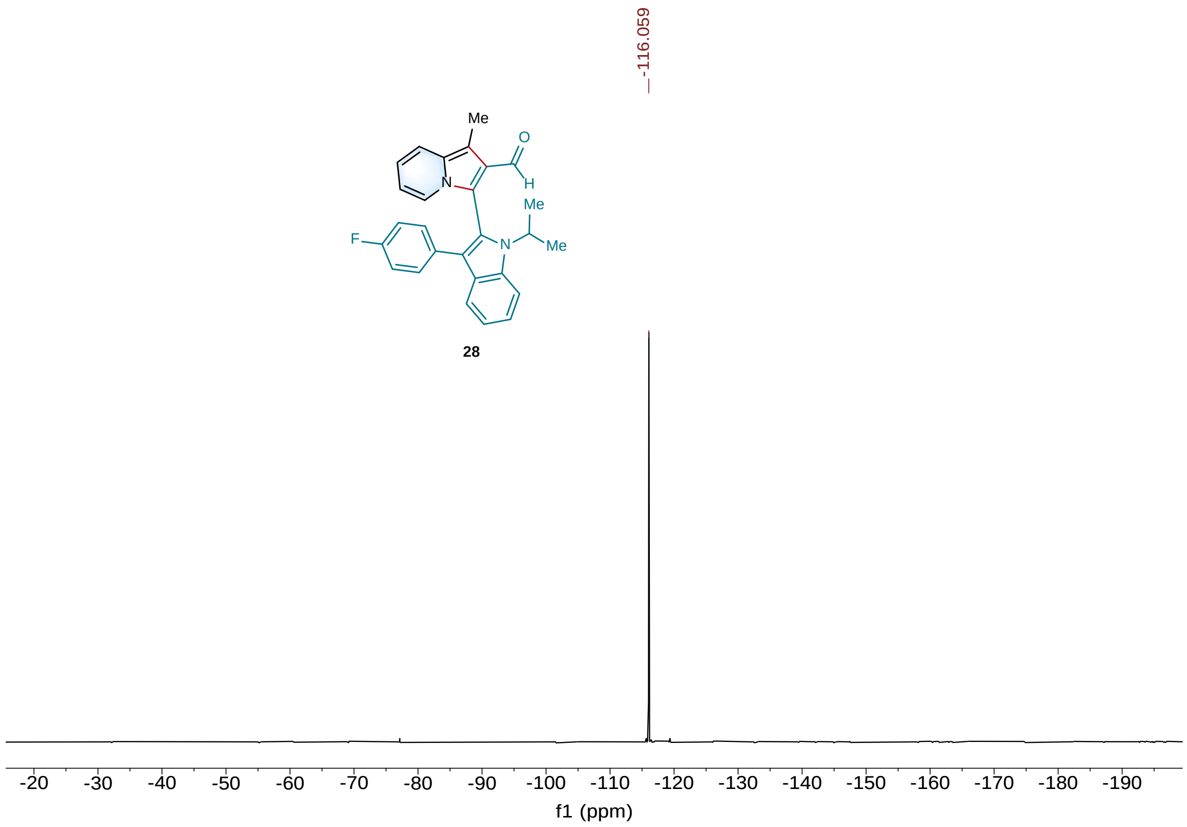

^{1}H NMR spectra of 29 (400 MHz, $CDCl_3$)

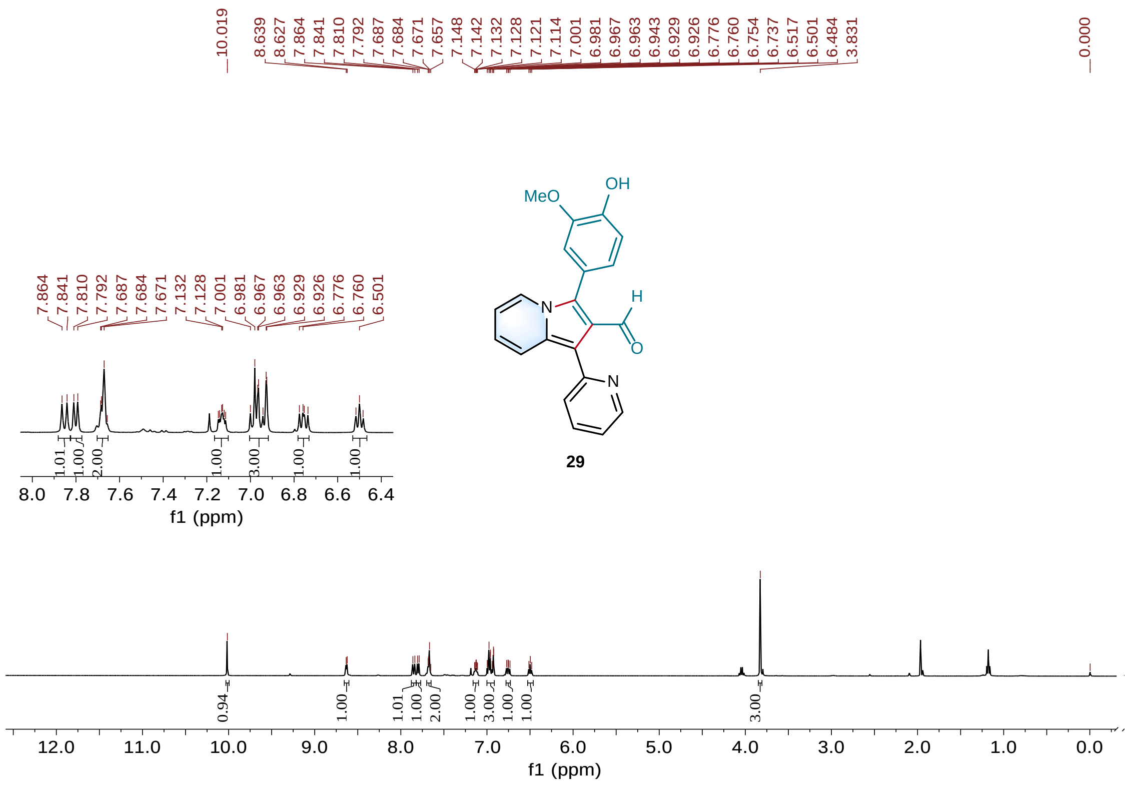

^{13}C NMR spectra of 29 (100 MHz, $CDCl_3$)

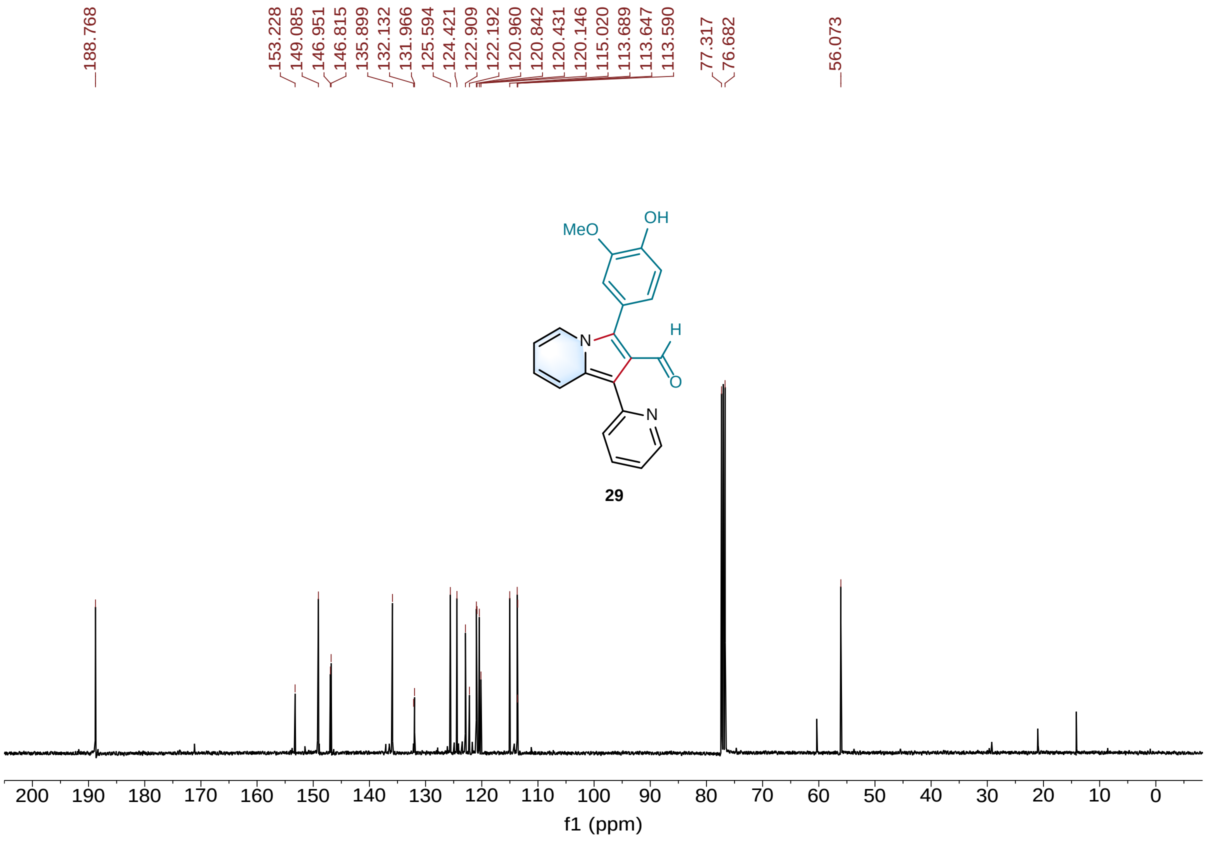

^{1}H NMR spectra of 30 (400 MHz, $CDCl_3$)

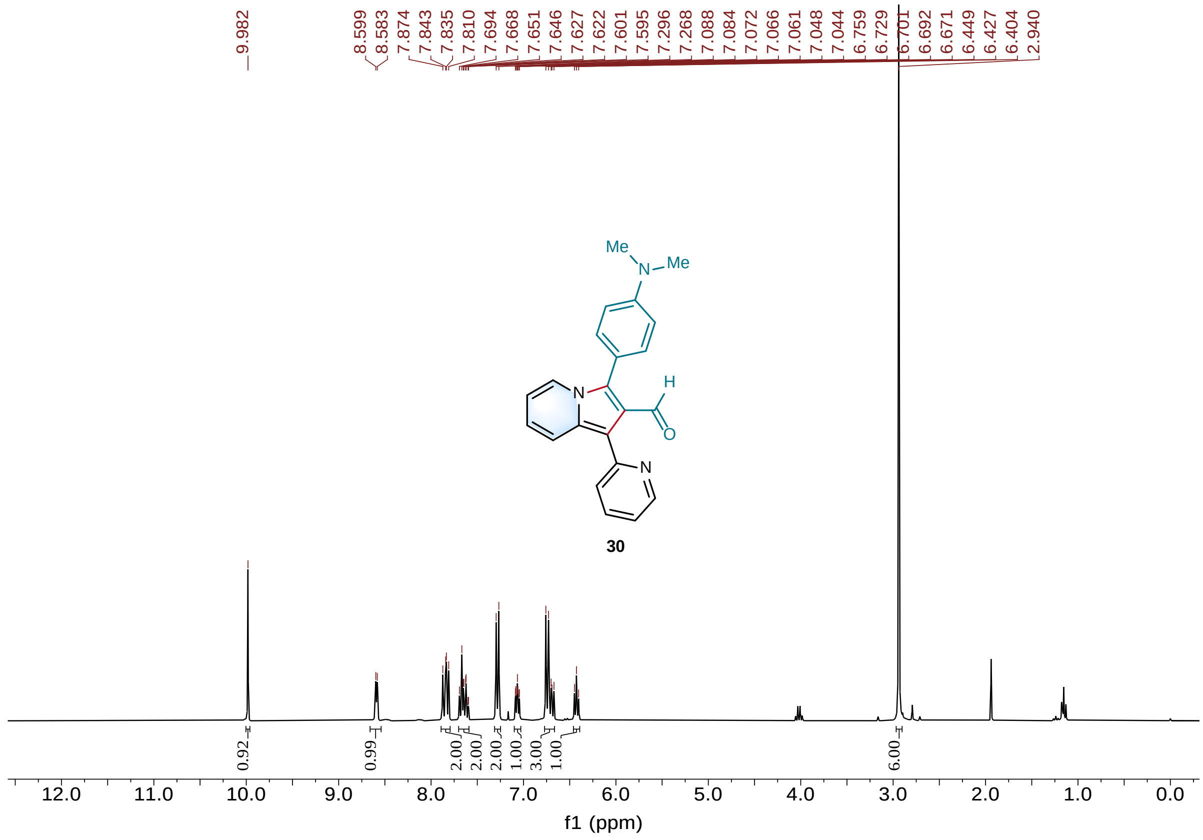

^{13}C NMR spectra of 30 (100 MHz, $CDCl_3$)

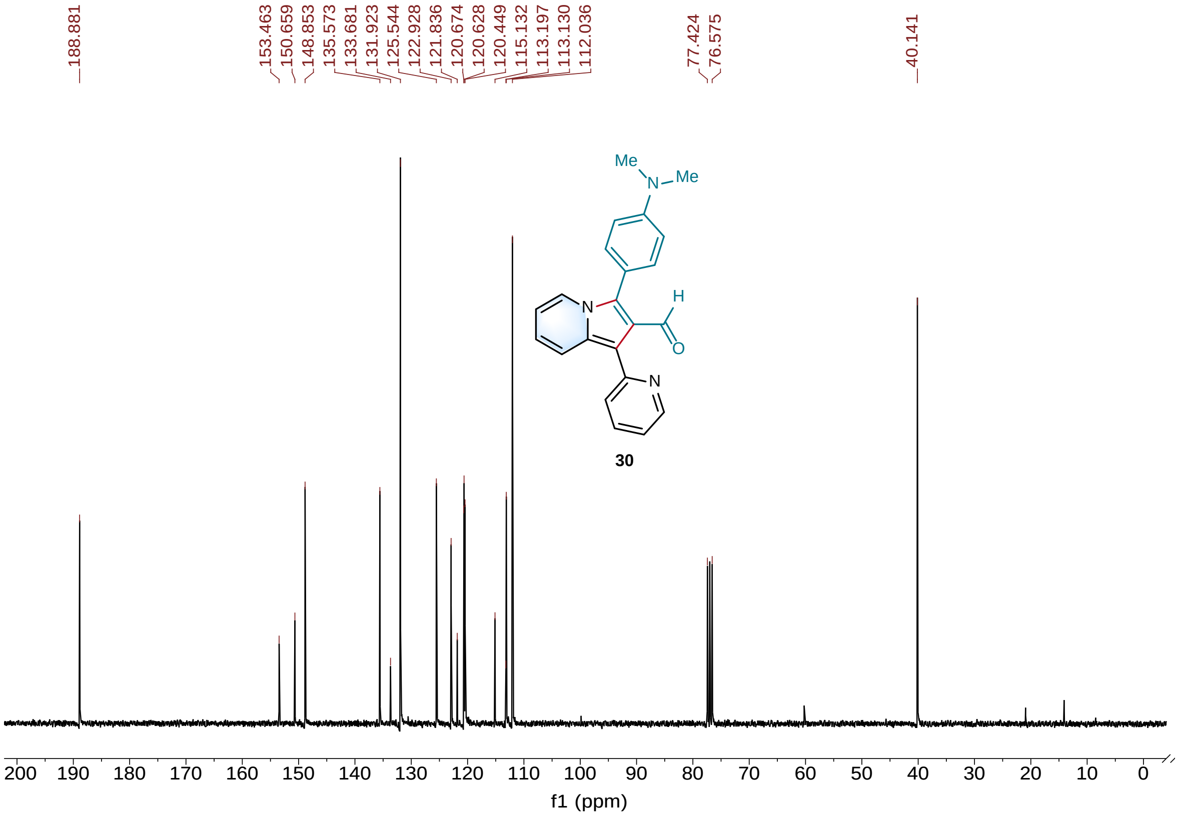

^{1}H NMR spectra of 31 (400 MHz, $CDCl_3$)

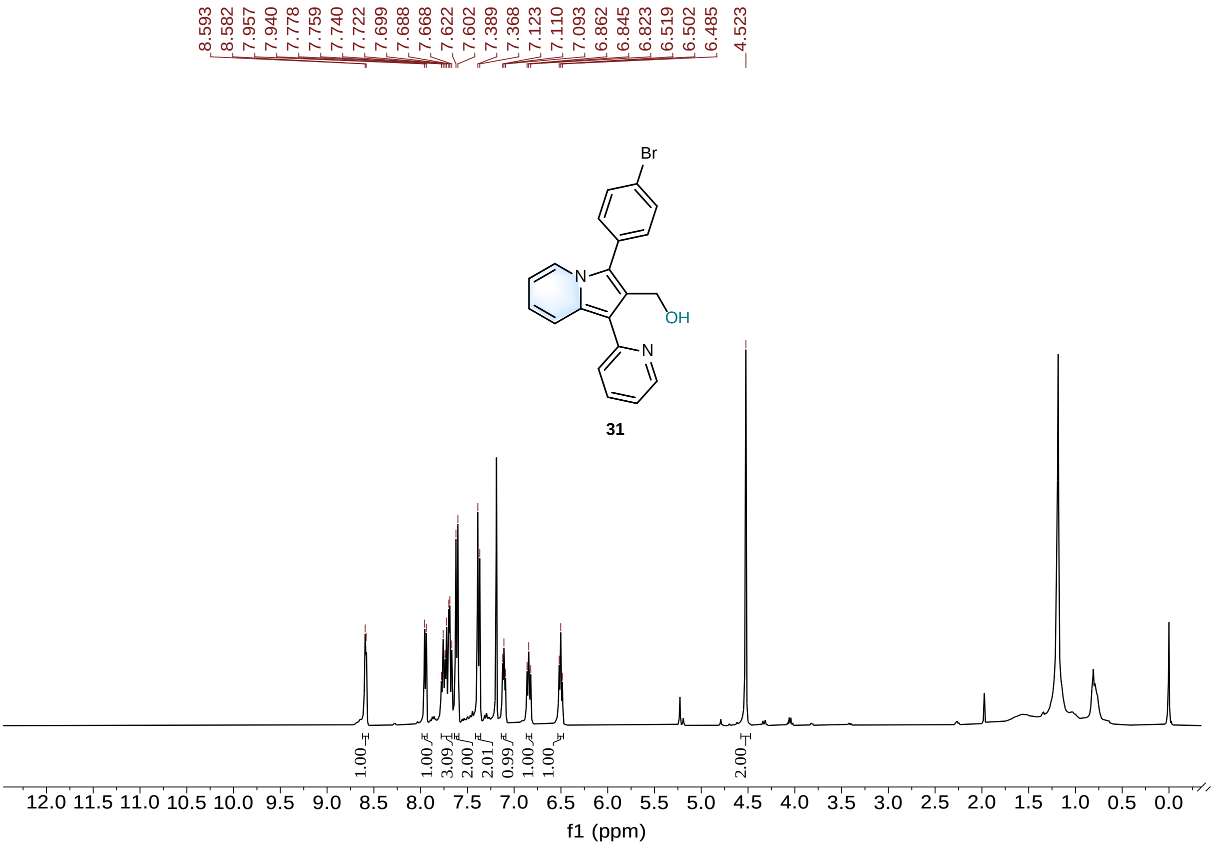

^{13}C NMR spectra of 31 (100 MHz, $CDCl_3$)

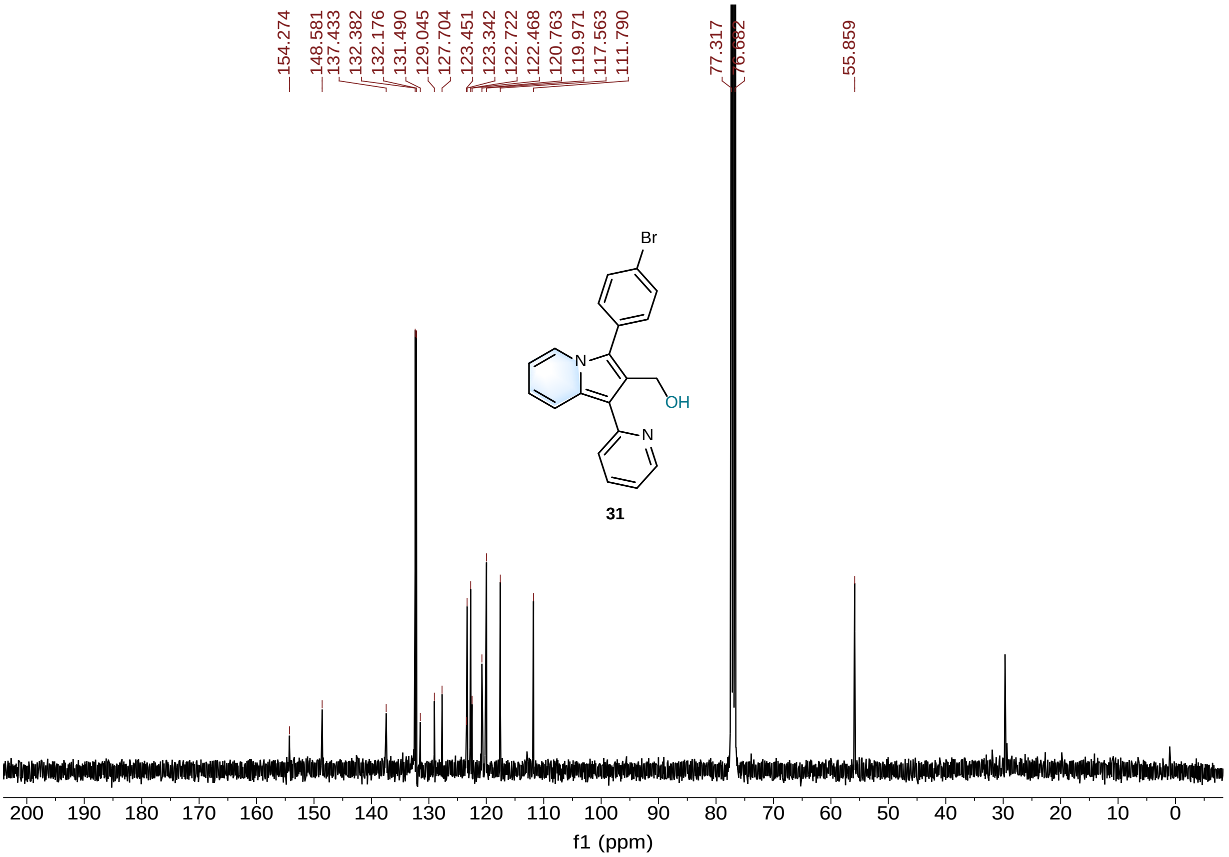

^{1}H NMR spectra of 32 (400 MHz, $CDCl_3$)

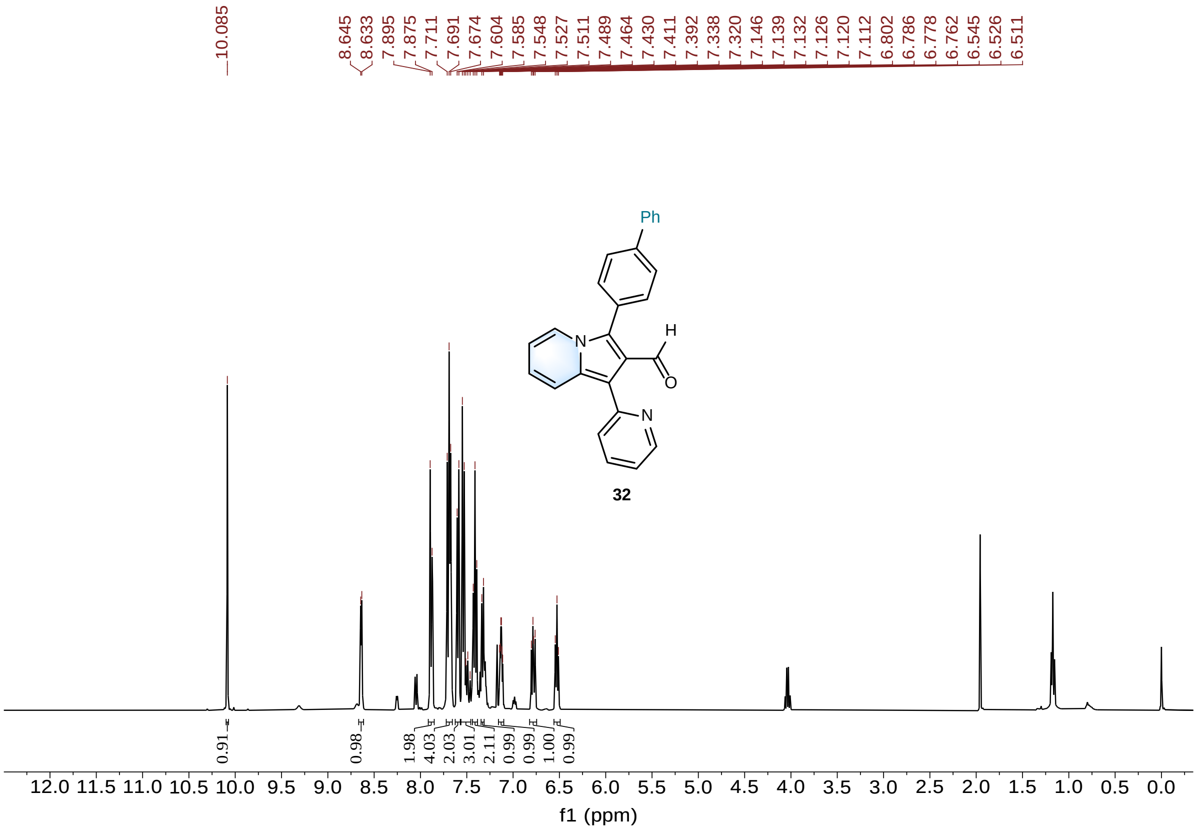

^{13}C NMR spectra of 32 (100 MHz, CDCl$_3$)

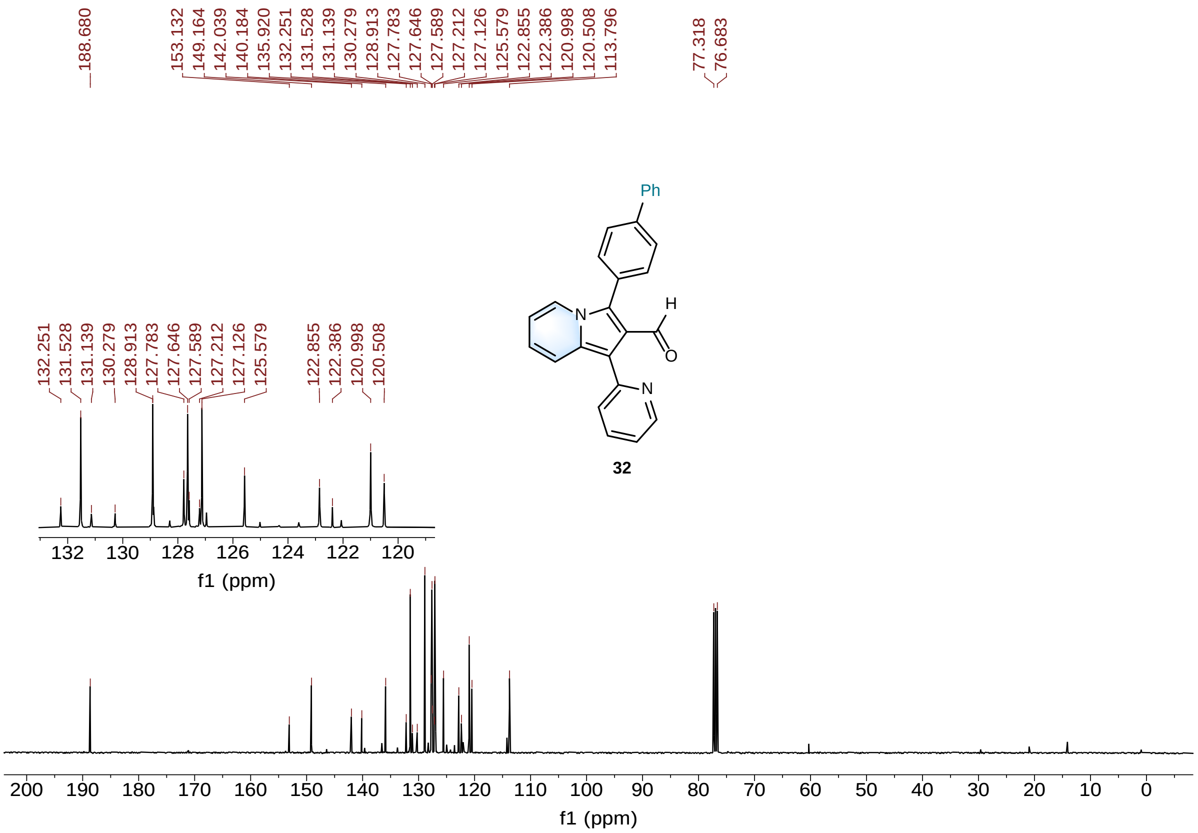

^{1}H NMR spectra of 33 (400 MHz, CDCl$_3$)

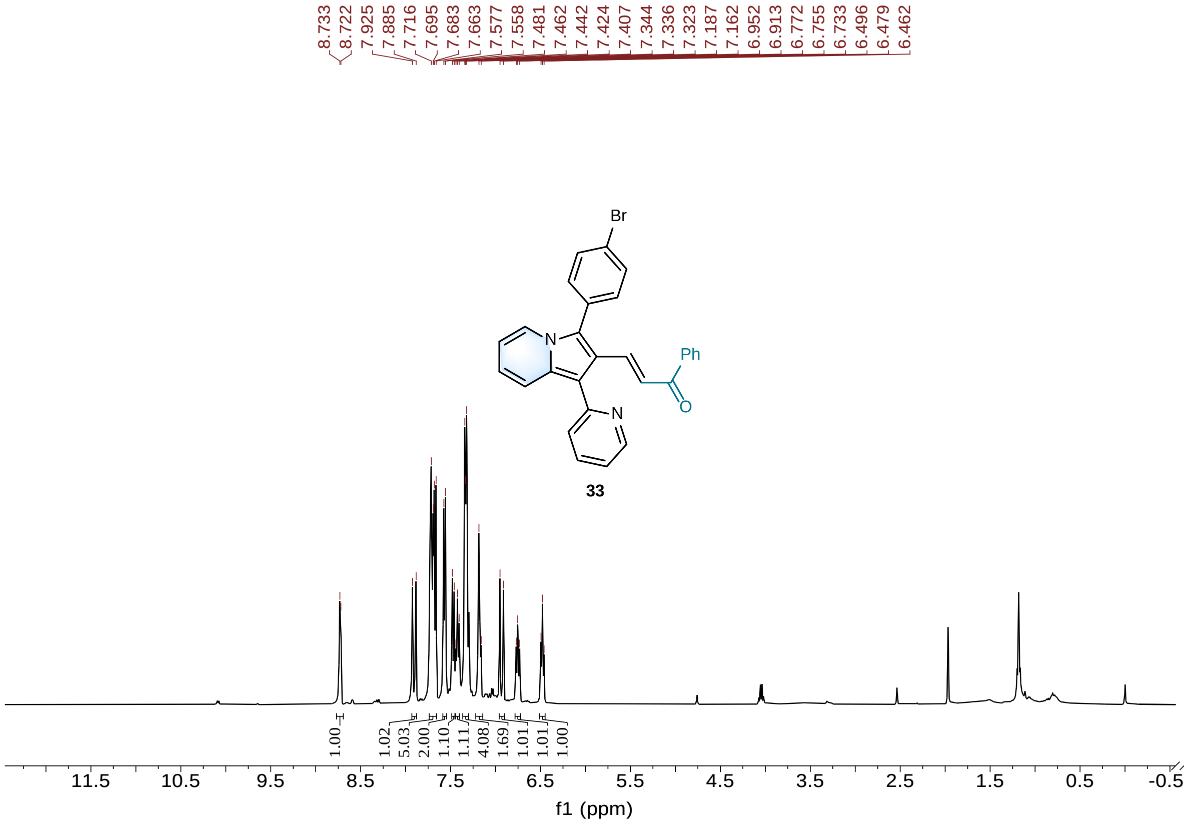

^{13}C NMR spectra of 33 (100 MHz, $CDCl_3$)

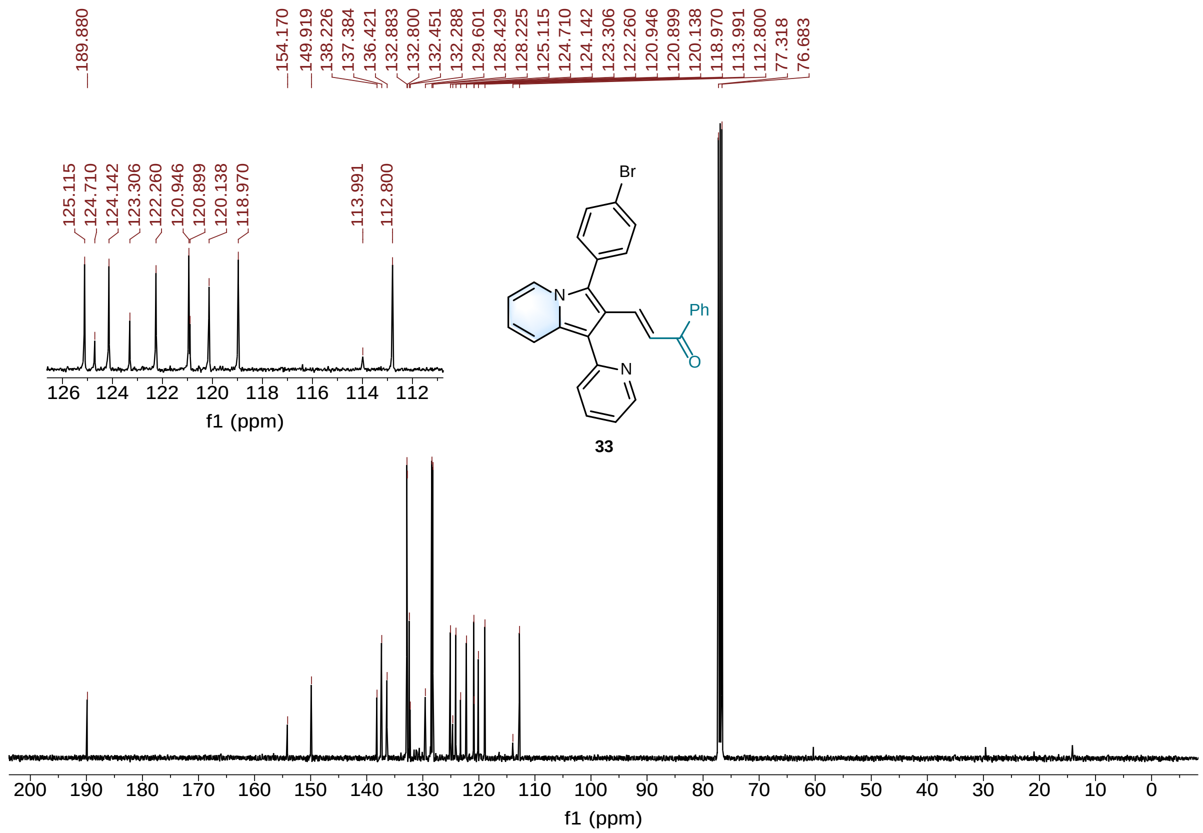

^{1}H NMR spectra of 34 (400 MHz, DMSO-d_6)

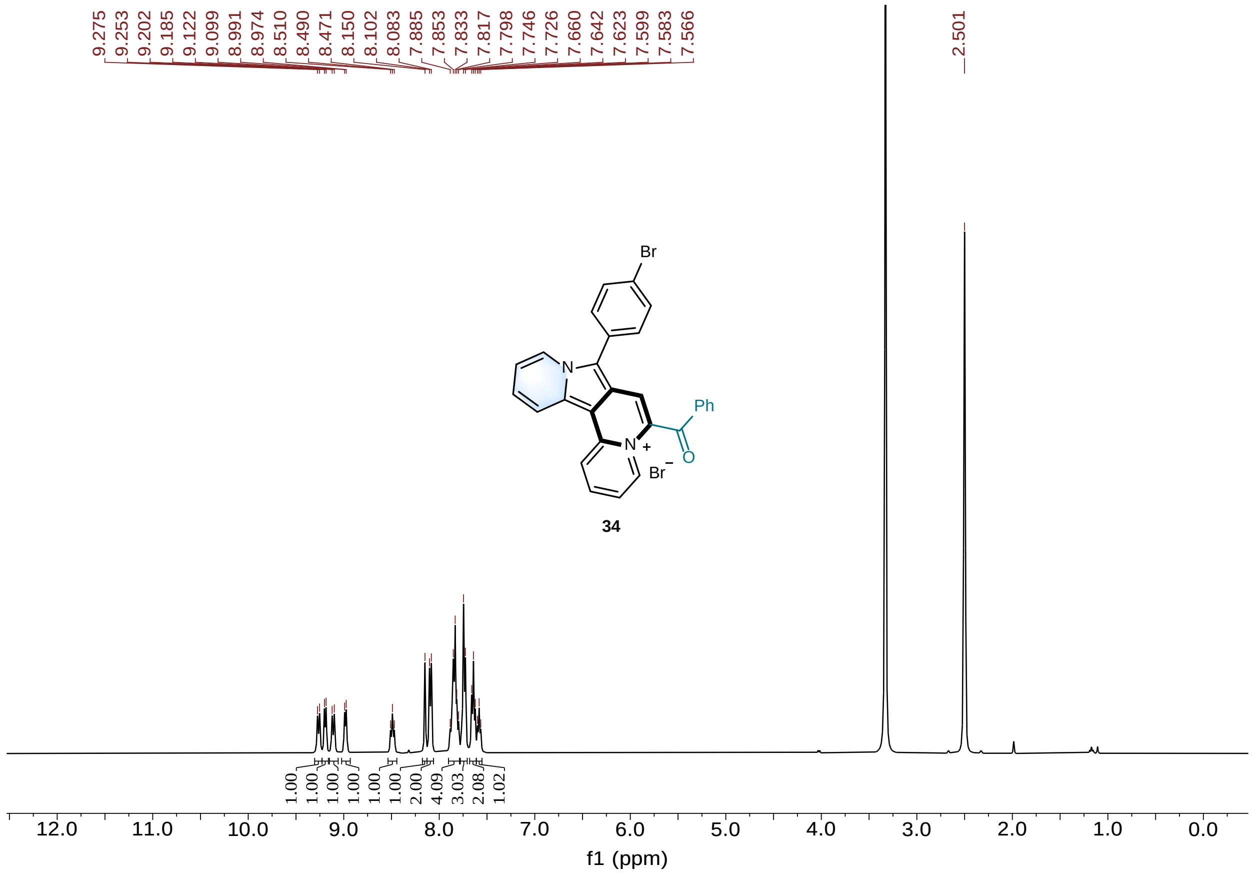

^{13}C NMR spectra of 34 (100 MHz, DMSO-d$_6$)

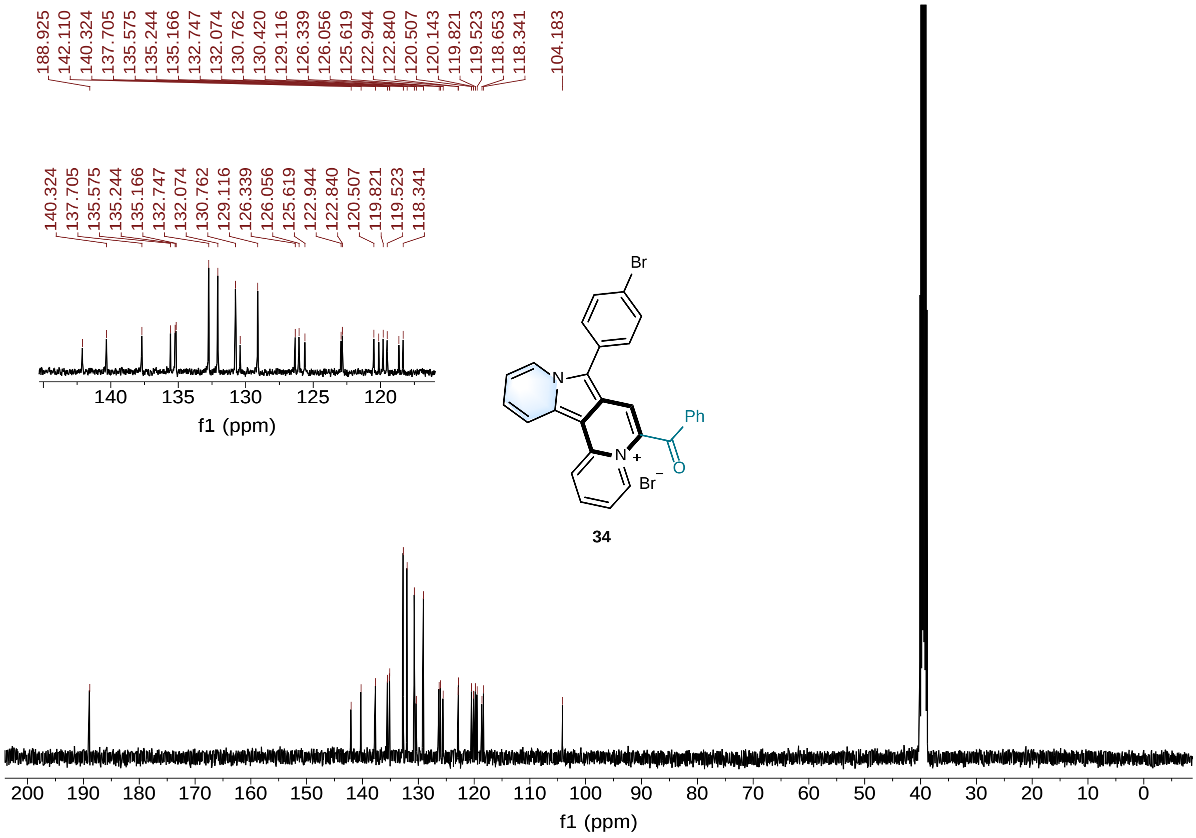

^{1}H NMR spectra of 35 (400 MHz, CDCl$_3$)

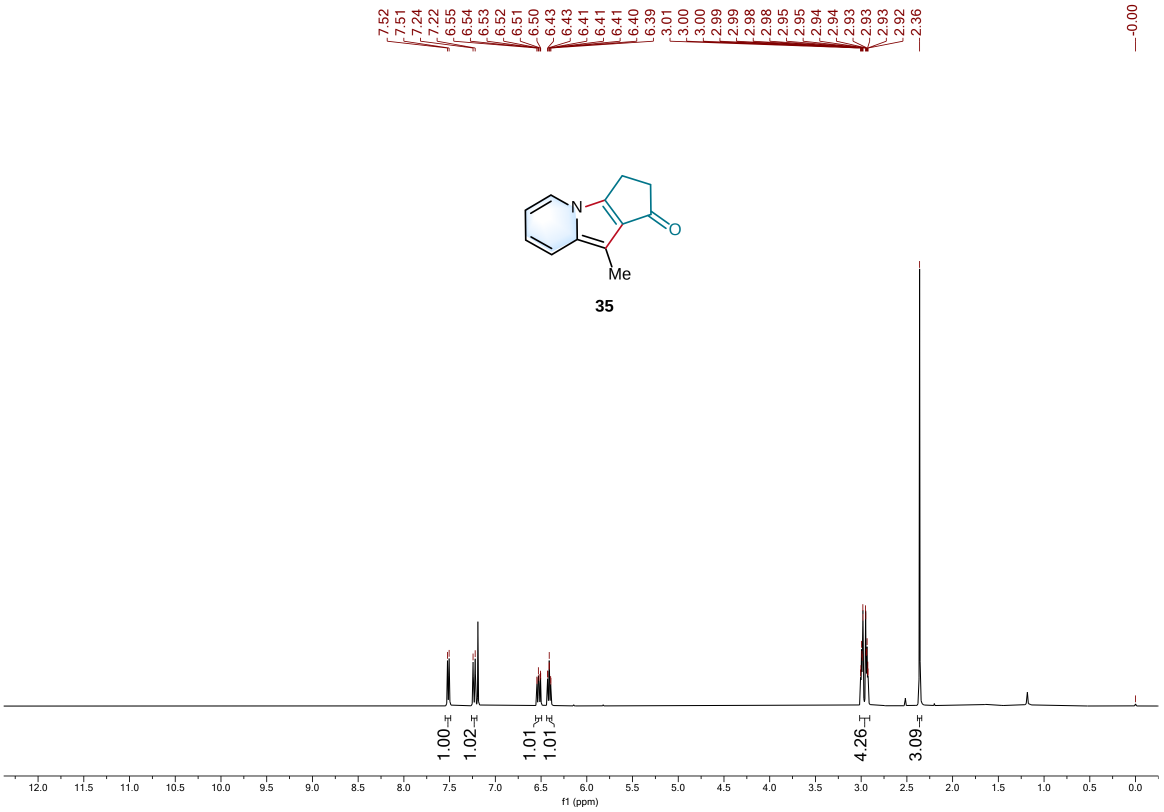

^{13}C NMR spectra of 35 (100 MHz, CDCl$_3$)

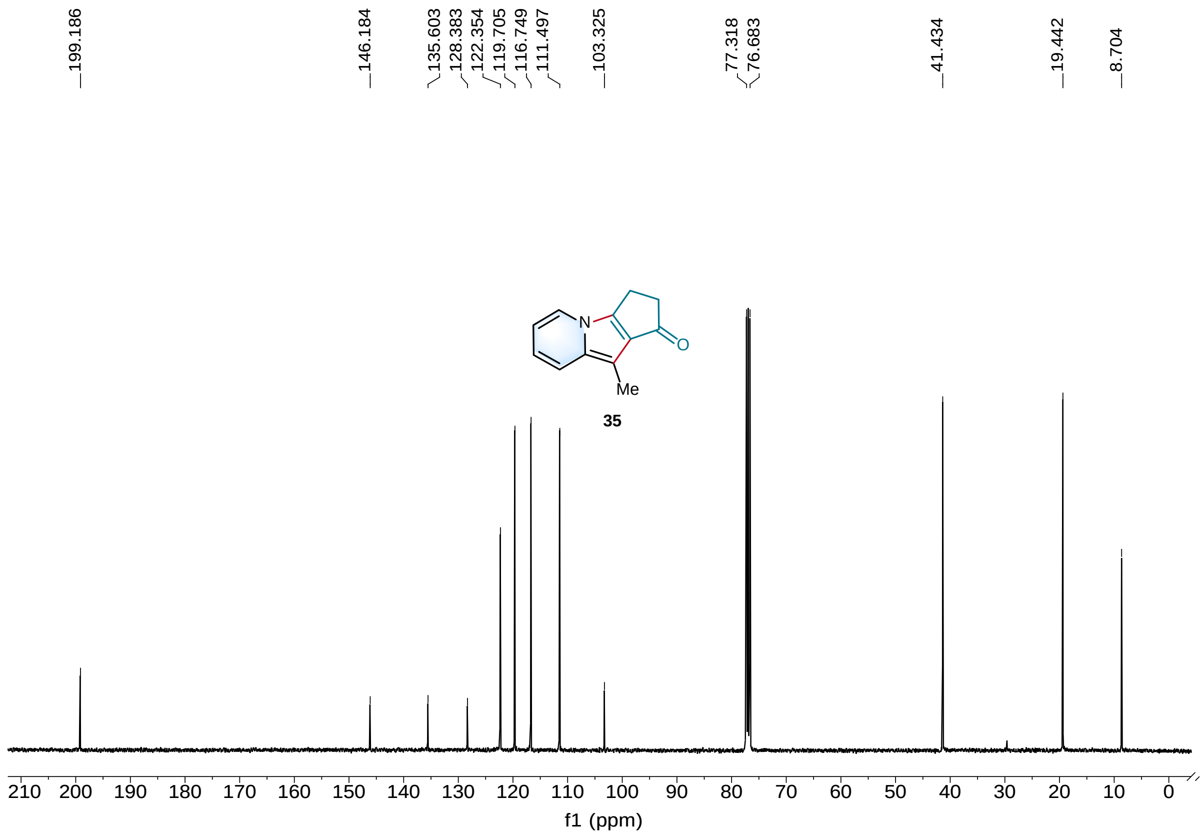

^{1}H NMR spectra of 36 (400 MHz, CDCl$_3$)

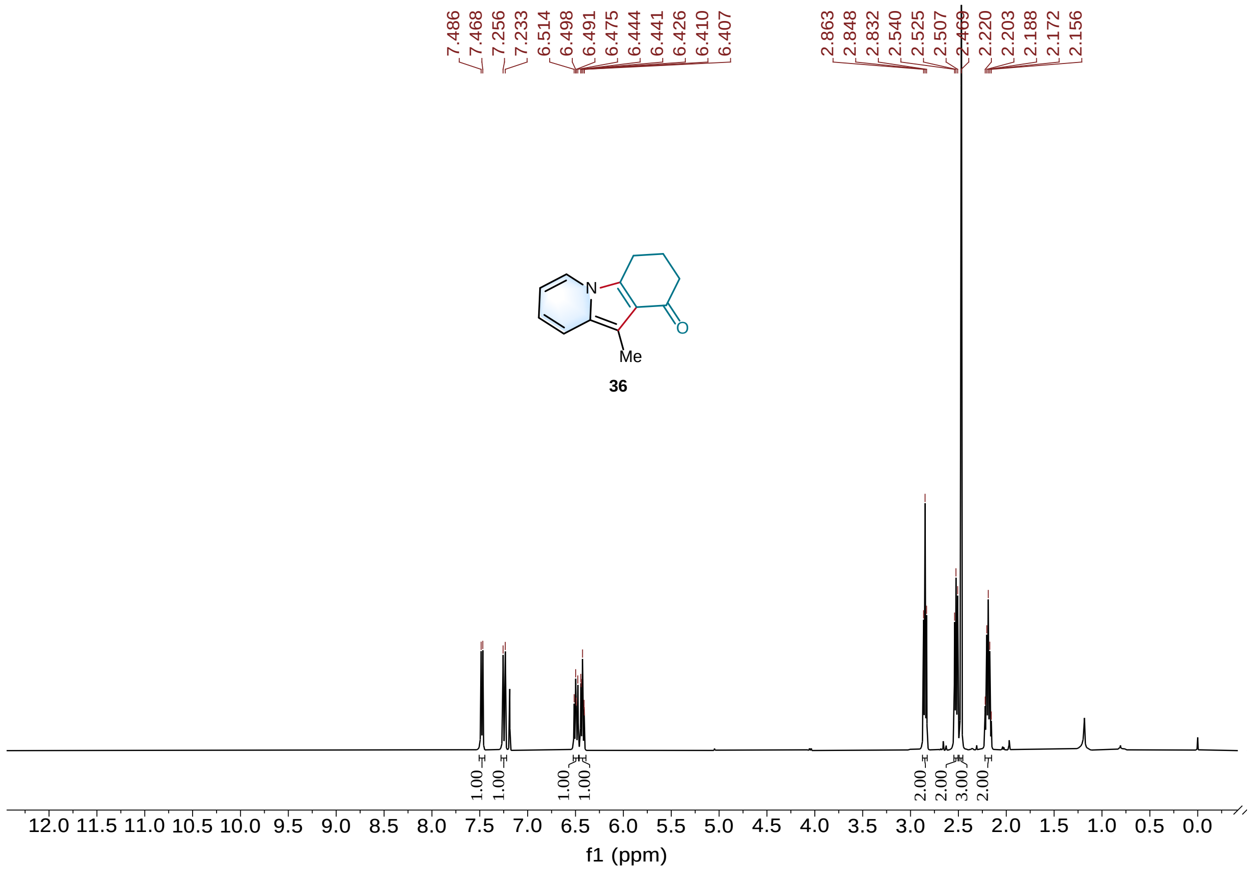

^{13}C NMR spectra of 36 (100 MHz, $CDCl_3$)

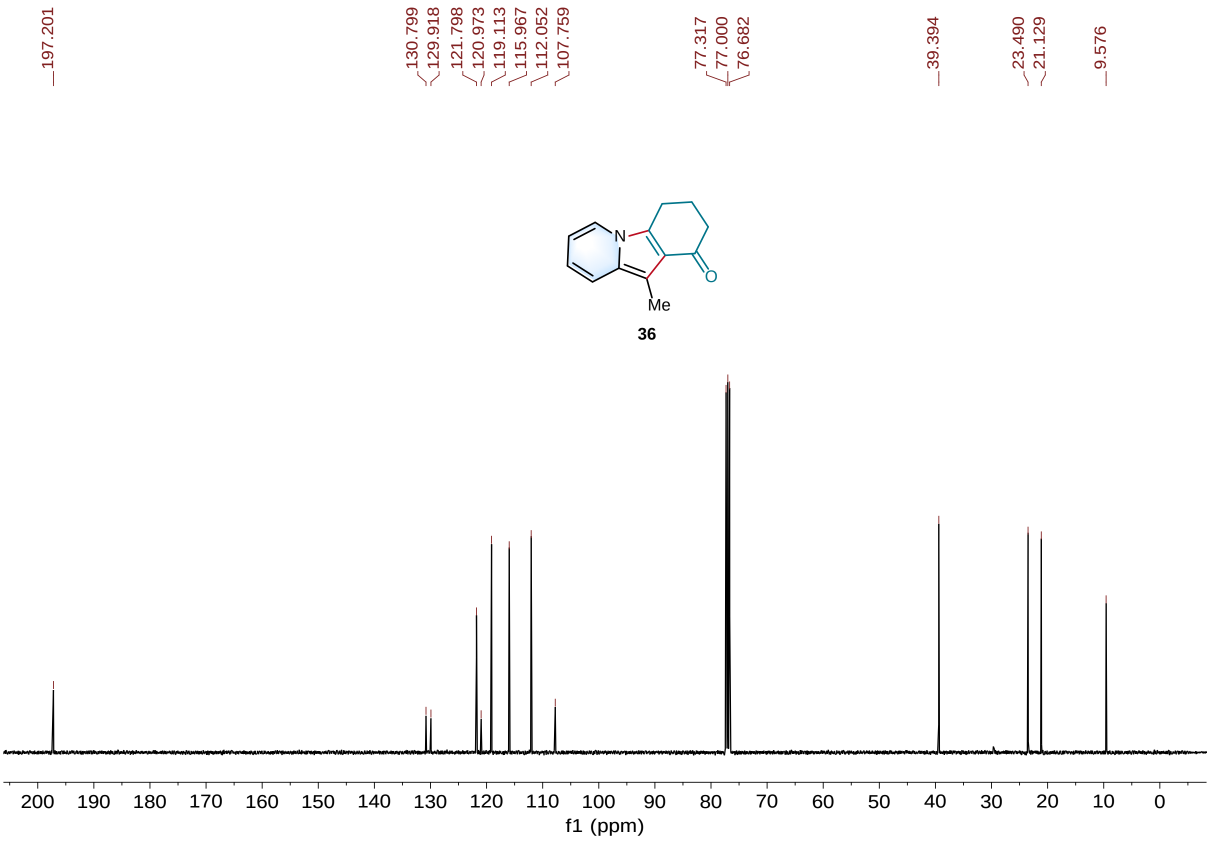

^{1}H NMR spectra of 37 (400 MHz, $CDCl_3$)

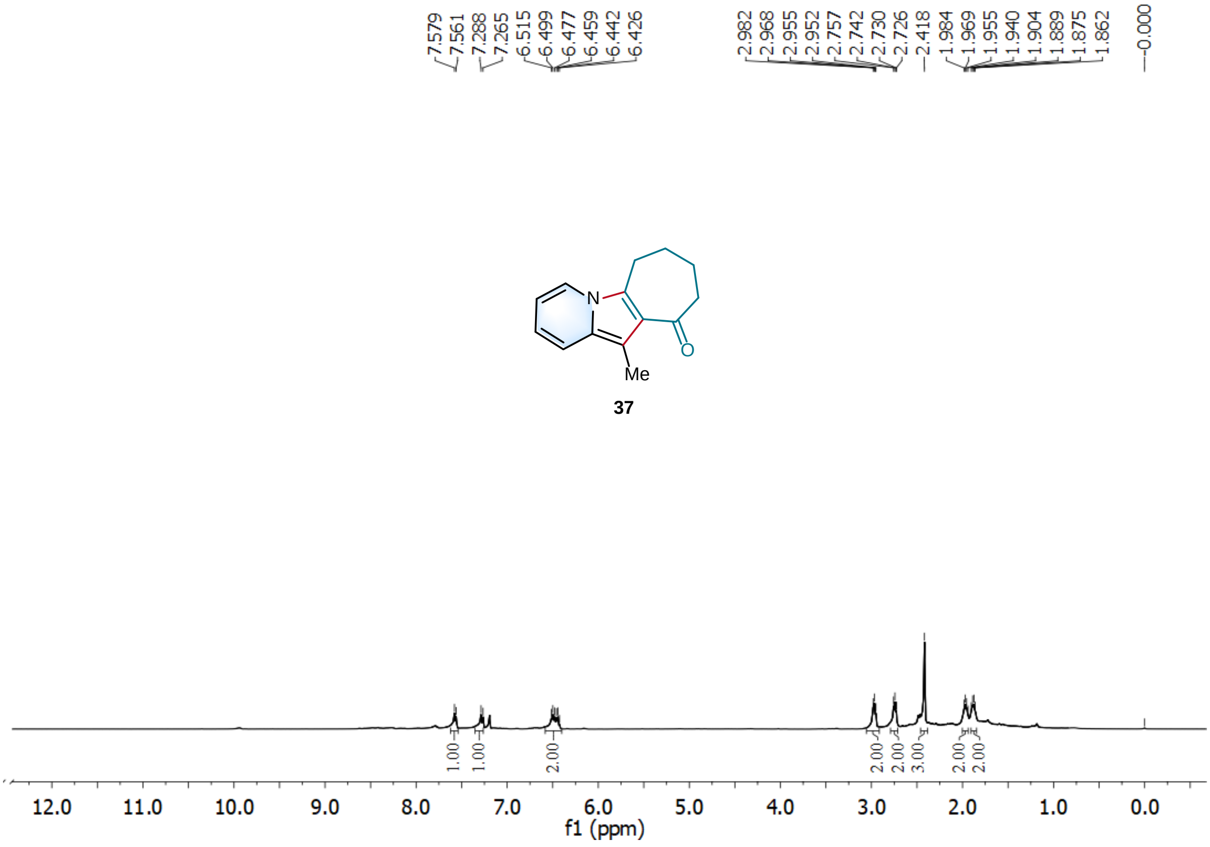

^{13}C NMR spectra of 37 (100 MHz, CDCl$_3$)

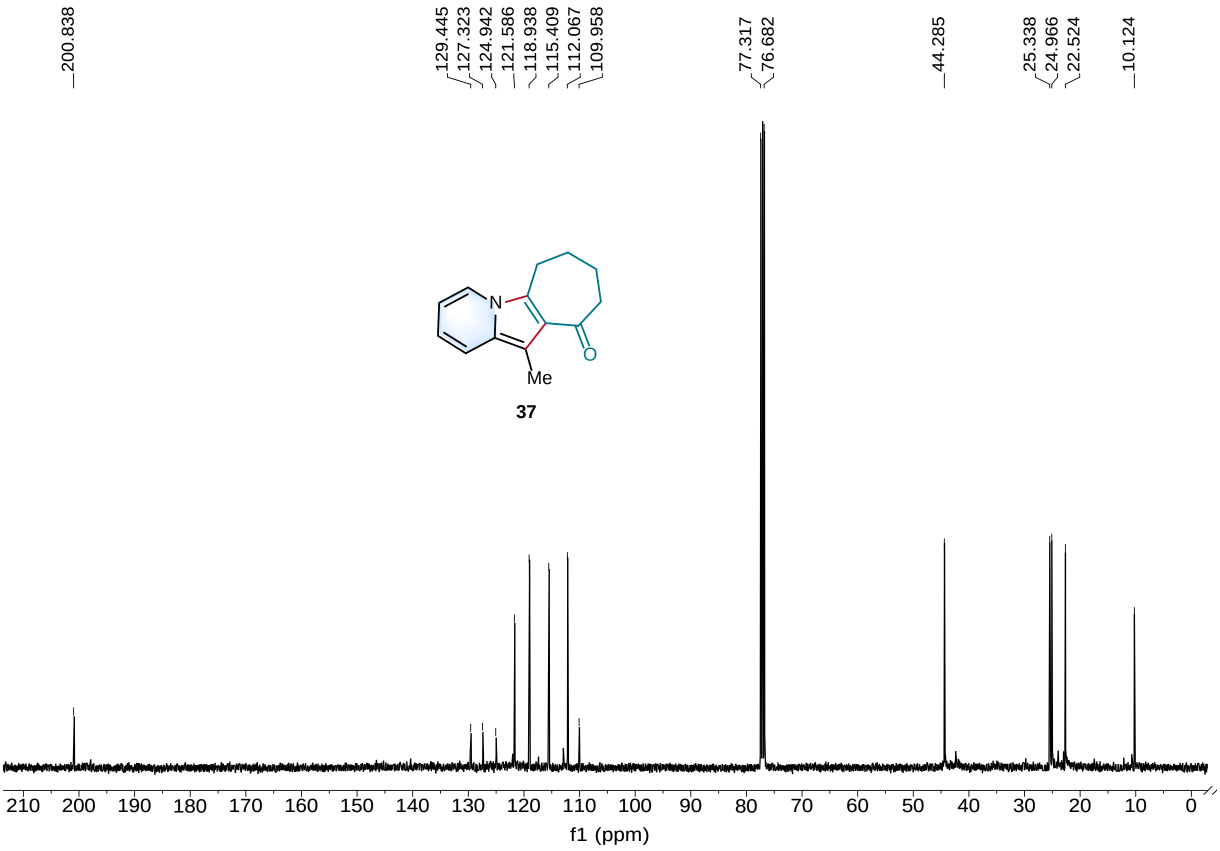

Supplement: Supplementary file 3 — Supplementary Data 1 [file 42004_2023_828_MOESM3_ESM.pdf]
